# New Polyketides from a Marine Sponge-Derived Fungus, *Neopestalotiopsis* sp., with Anti-Renal Fibrosis Activity

**DOI:** 10.3390/md23040148

**Published:** 2025-03-29

**Authors:** Xinlong Li, Jianglian She, Meiqun Cai, Xinqi Chen, Rongxiang Qiu, Xiaowei Luo, Yonghong Liu, Xuefeng Zhou, Lan Tang

**Affiliations:** 1 Guangxi Key Laboratory of Marine Drugs, Institute of Marine Drugs, Guangxi University of Chinese Medicine, Nanning 530200, China; 13409379936@163.com (X.L.); luoxiaowei1991@126.com (X.L.); yonghongliu@scsio.ac.cn (Y.L.); 2 CAS Key Laboratory of Tropical Marine Bio-Resources and Ecology, Guangdong Key Laboratory of Marine Materia Medica, South China Sea Institute of Oceanology, Chinese Academy of Sciences, Guangzhou 510301, China; shejianglian20@mails.ucas.ac.cn (J.S.); scsiochenxinqi@163.com (X.C.); 3 NMPA Key Laboratory for Research and Evaluation of Drug Metabolism, Guangdong Provincial Key Laboratory of New Drug Screening, School of Pharmaceutical Sciences, Southern Medical University, Guangzhou 510515, China; meiquncai@163.com (M.C.); 18707678946@163.com (R.Q.); 4 University of Chinese Academy of Sciences, Beijing 100049, China; 5 School of Chinese Materia Medica, Guangdong Pharmaceutical University, Guangzhou 510006, China

**Keywords:** sponge-associated fungi, polyketides, *Neopestalotiopsis*, anti-renal fibrosis

## Abstract

Sixteen polyketides, including six new compounds (**1**–**2**, and **5**–**8**), were isolated from the culture of the marine sponge-associated fungus *Neopestalotiopsis* sp. SCSIO 41422. Their structures were elucidated through NMR, MS spectroscopic analyses, calculated electronic circular dichroism, quantum chemical NMR calculations, and X-ray single-crystal diffraction. To screen and evaluate the inhibitory activity of these polyketides in renal fibrosis, a TGF-*β*1-stimulated HK-2 cell model was used. All tested compounds (**1**, **5**–**8**, and **11**–**12**) at 10 µM showed obvious anti-fibrotic activity by inhibiting TGF-*β*1-induced *α*-SMA expression and extracellular matrix production (collagen I and fibronectin). Among them, gamahorin A (**1**) was shown to be the most potent and the most promising inhibitor against renal fibrosis.

## 1. Introduction

Chronic kidney disease (CKD) has become a significant global health concern, affecting roughly 700 million individuals [1,2]. Chronic inflammation and renal fibrosis drive the disease, with conditions like chronic glomerulonephritis, diabetic nephropathy, and hypertensive nephropathy sharing common pathological features of fibrosis, leading to end-stage renal failure [3,4]. Kidney damage causes nephron shrinkage, fibroblast transformation into myofibroblasts, and excessive extracellular matrix (ECM) accumulation [5]. The ECM consists of a fibrous network with gel-like components, including collagen, elastin, fibronectin, and laminin. Collagen, mainly produced by myofibroblasts, plays a central role in fibrosis. Fibroblast-to-myofibroblast conversion is key in renal fibrosis and a therapeutic target [6]. Current clinical trials of anti-fibrosis drugs show limited efficacy and significant side effects [7]. Pirfenidone (PFD) showed only a transient improvement in Phase II trials, with some patients discontinuing due to adverse effects, such as rash and gastrointestinal issues [8]. PFD’s pharmacokinetics depend on renal function, limiting its clinical applicability [9]. Therefore, developing novel, effective, and safe anti-fibrosis drugs is crucial.

**Figure 1:**
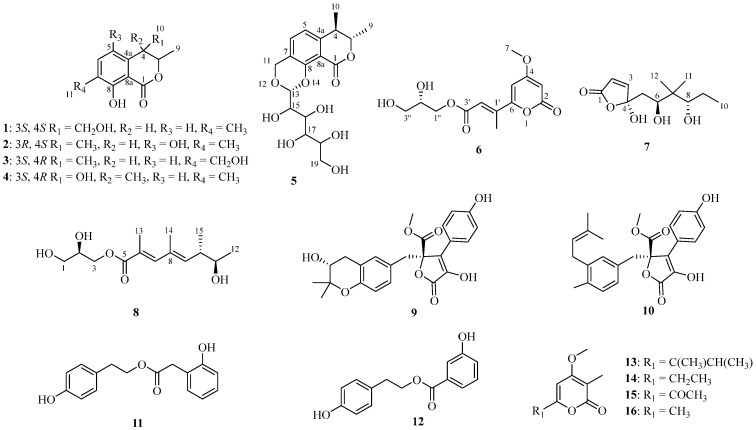
Structures of compounds **1**–**16**.

Marine natural products are a new source for drug discovery, and studies show that many of them have potent anti-inflammatory and antioxidant properties, which are crucial in mitigating renal fibrosis. Penicilliumin B, a methyl cyclopentadienone from a deep-sea *Penicillium* strain, inhibits high-glucose-induced fibrosis in renal tubular cells. It also reduces oxidative stress and has strong anti-fibrotic and anti-proliferative properties [10]. Extreme marine environments have driven the evolution of marine fungi with exceptional adaptability, facilitating the production of diverse secondary metabolites with notable biological activity [11,12,13,14]. More than 3166 new compounds have been derived from sponges and their associated microorganisms, making them the second-largest source of marine natural products [15,16]. In the pursuit of anti-renal fibrosis lead compounds, six new and ten reported polyketides (Figure 1) were isolated from the sponge-associated fungus *Neopestalotiopsis* sp. SCSIO 41422. This study details the isolation, structural elucidation, and evaluation of the anti-renal fibrosis activity of the compounds, providing new insights into potential treatments for renal fibrosis.

## 2. Results and Discussion

### 2.1. Structural Determination

**Figure 2:**
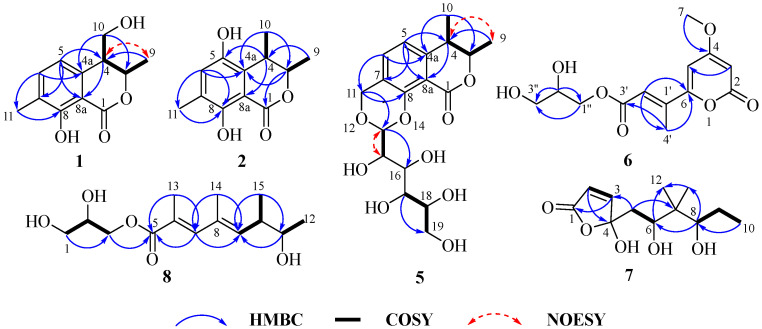
Key HMBC, COSY, and NOESY correlations of compounds **1**–**2** and **5**–**8**.

**Figure 3:**
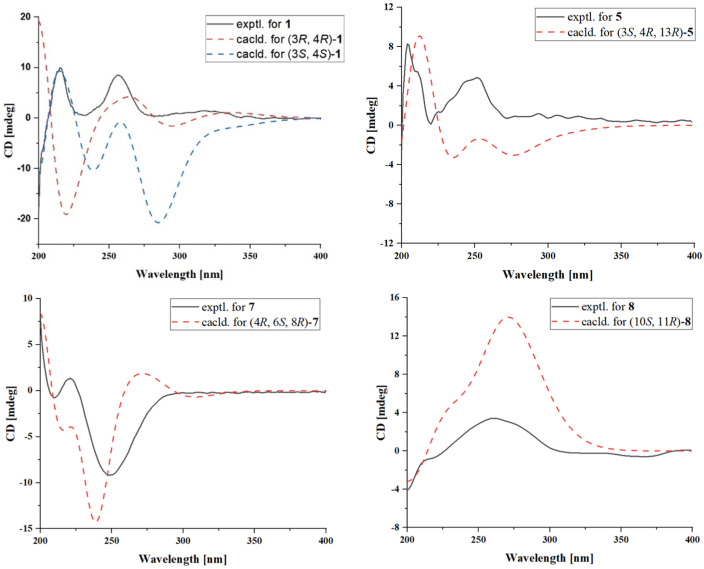
Experimental and calculated ECD spectra of compounds **1**, **5**, and **7**–**8**.

Compound **1** was isolated as a brown oil, and its molecular formula was established as C_12_H_14_O_4_ based on HRESIMS data, indicating six degrees of unsaturation. The 1D NMR and HSQC spectra (Table 1) revealed the following characteristic signals: two methyl groups [*δ*_C/H_ 19.9/1.37 (CH_3_-9), 15.5/2.22 (CH_3_-11)], two olefinic methine carbons [*δ*_C/H_ 120.1/6.79 (CH-5), 138.4/7.39 (CH-6)], one methylene carbon [*δ*_C/H_ 64.7/3.66 (CH_2_-10)], and two methine carbons [*δ*_C/H_ 77.6/5.05 (CH-3), 46.7/2.89 (CH-4)], along with five quaternary carbons [*δ*_C_ 170.5 (C-1), 137.9 (C-4a), 126.6 (C-7), 161.3 (C-8), 108.2 (C-8a)]. The ^1^H and ^13^C NMR data of **1** closely resembled those of gamahorin [17], with the primary difference being the conversion of the methyl group at C-10 into a hydroxymethyl group and the hydroxymethyl group at C-11 into a methyl group, respectively. The HMBC (Figure 2) correlations of H-11/C-6, C-8 and H-10/C-3, C-4a further supported this deduction. Furthermore, the ^1^H-^1^H COSY spectrum revealed a correlation between H-10 and H-4, which provided additional confirmation of the proposed structure. The NOESY spectrum showed a correlation between H-4 and H-9, indicating that H-4 and CH_3_-9 were positioned on the same face of the molecule. The vicinal coupling constant (*J* = 5.6 Hz) between the two methine protons (H-3 and H-4) at position 3 supports the *threo* stereochemistry [17]. Based on this, the relative configuration of **1** was determined as 3S*, 4S*-**1**, 3R*, 4R*-**1**. The absolute configurations were subsequently established through computational electronic circular dichroism (ECD) analysis. The calculated ECD spectrum of 3*S*, 4*S*-**1** closely matched the experimental ECD spectrum (Figure 3), confirming the absolute configuration as 3*S*, 4*S*. In conclusion, the structure of **1** was elucidated as depicted in Figure 1, and it was named gamahorin A (**1**).

**Figure 4:**
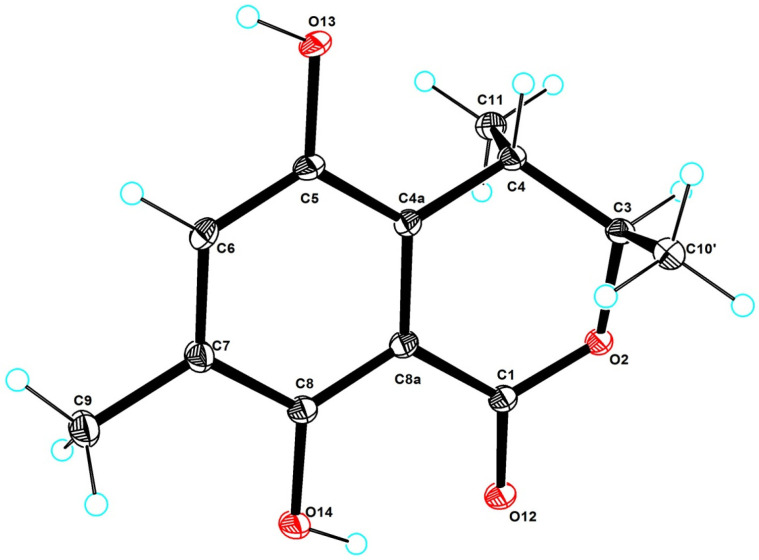
X-ray single-crystal diffraction of **2**.

Compound **2** was isolated as a yellow solid, with its molecular formula established as C_12_H_14_O_4_ based on HRESIMS data. The one-dimensional NMR and HSQC spectra (Table 1) displayed the following characteristic signals: three methyl groups [*δ*_C/H_ 20.3/1.30 (CH_3_-9), 19.9/1.27 (CH_3_-10), 15.5/2.17 (CH_3_-11)], one olefinic methine carbon [*δ*_C/H_ 126.6/6.95 (CH-6)], two methine carbons [*δ*_C/H_ 83.0/4.73 (CH-3), 32.7/3.19 (CH-4)], and six quaternary carbons [*δ*_C_ 170.7 (C-1), 126.5 (C-4a), 147.0 (C-5), 126.0 (C-7), 154.7 (C-8), 107.0 (C-8a)]. The structure of **2** closely resembled that of **1**, with the primary difference being the location of the hydroxyl group. In the structure of **1**, the hydroxyl group is located at C-10, whereas, in **2**, it is attached to C-5. The proposed structure of **2** was further substantiated by ¹H-¹H COSY and HMBC correlations (Figure 2). The ¹H-¹H COSY correlations observed between H-3/H-9 and H-4/H-10, together with the HMBC correlations of H-10/C-3, C-4a, H-4/C-5, C-8a and H-3/C-10, C-4a, and C-1, provided strong support for the structural assignment. To determine the absolute configuration of the compound, X-ray diffraction analysis was performed, confirming both the planar structure and the absolute configuration as (3*R*, 4*S*-**2**) (Figure 4). Consequently, **2** was identified as gamahorin B (**2**).

**Figure 5:**
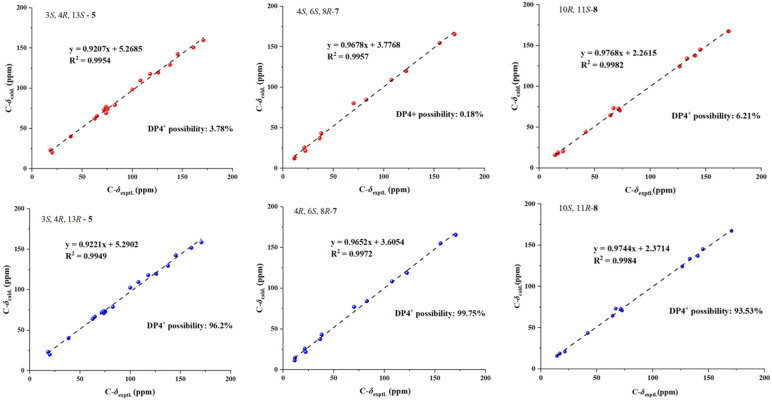
Linear regression and DP4 possibility analysis between the experimental and calculated ^1^H and ^13^C chemical shifts of the diastereomers of (3*S*,4*R*,13*R*)-**5**/(3*S*,4*R*,13*S*)-**5**, (4*S*,6*S*,8*R*)-**7**/(4*R*,6*S*,8*R*)-**7**, and (10*R*,11*S*)-**8**/ (10*S*,11*R*)-**8**.

Compound **5** was isolated as a yellow solid, with its molecular formula established as C_18_H_24_O_9_ based on HRESIMS data, suggesting seven degrees of unsaturation. The one-dimensional NMR (Table 1) and HSQC spectra displayed characteristic signals, including two methyl groups [*δ*_C/H_ 19.9/1.43 (CH_3_-9), 18.2/1.36 (CH_3_-10)], two olefinic methines [*δ*_C/H_ 117.9/6.90 (CH-5), 137.6/7.74 (CH-6)], two methylene groups [*δ*_C/H_ 64.7/4.89, 4.61 (CH_2_-11); 62.5/3.77, 3.70 (CH_2_-19)], seven methine groups [*δ*_C/H_ 82.6/4.57 (CH-3), 38.5/2.96 (CH-4); 100.0/4.95 (CH-13), 73.7/3.44 (CH-15), 75.1/3.70 (CH-16), 71.7/3.34 (CH-17), 73.8/3.66 (CH-18)], and five quaternary carbons [*δ*_C_ 170.9 (C-1), 145.3 (C-4a), 125.9 (C-7), 160.9 (C-8), 108.2 (C-8a)]. The ¹H and ¹³C NMR spectral data of **5** revealed structural similarities to gamahorin [17]. The NOESY spectrum revealed a correlation between H-4 and H-9, indicating that H-4 and CH_3_-9 are positioned on the same face of the molecule. The vicinal coupling constant (*J* = 7.0 Hz) between the two methine protons (H-3 and H-4) at position 4 supports the *threo* stereochemistry. To determine the absolute configurations at the C-3, C-4, and C-13 rigid-ring positions of **5**, we employed DP4^+^ probability analysis combined with NMR calculations utilizing the GIAO method. Through the prediction and analysis of NMR chemical shifts for four potential isomers (3S*,4R*,13R*-**5**, 3R*,4S*,13R*-**5**, 3S*,4R*,13S*-**5**, 3R*,4S*,13S*-**5**), the results identified 3S*,4R*,13R*-**5** (Figure 5) as the most probable configuration, with a DP4^+^ probability of 96.22%. Furthermore, the experimental CD curve of **5** exhibited a strong match with the calculated ECD curve, thereby providing additional confirmation of its absolute configuration. Consequently, the absolute configuration of compound 5 was conclusively determined to be 3*S*,4*R*,13*R*-**5** (Figure 3). The HMBC spectrum (Figure 2) exhibited key correlations between H-6/C-11, H-11/C-8, C-13, confirming the formation of a six-membered ring through two ether bonds at C-7 and C-8 in 5. Additionally, a polyhydroxy side chain was identified as being substituted at C-13 of the six-membered ring. This substitution was supported by HMBC correlations between H-13/C-16, H-17/C-19, together with ¹H-¹H COSY correlations between H-13/H-15, H-17/H-18. The polyhydroxylated side chain in **5** suggests that it may be a glycosylated natural product, where the stereochemistry of its chiral centers is governed by the glycosylation process, typically consistent with the D-glucose configuration. The observed specific rotation ([*α*]25 D +11.05) and the presence of multiple chiral centers may influence the optical activity. A literature analysis revealed that coretinphenol (structurally analogous to **5**) releases its aglycone and D-glucose upon 2M HCl hydrolysis [18]. The hydrolysate was derivatized into trimethylsilyl (TMS) ethers and analyzed by GC-MS, with retention time matching against authentic references confirming the *β*-D-glucopyranosyl configuration. Attempts to isolate the side chain via acid hydrolysis were unsuccessful due to insufficient compound quantities. Collectively, while the core stereochemistry (3*S*,4*R,*13*R*) has been rigorously supported, the side-chain configuration remains unassigned, necessitating further structural characterization. Consequently, **5** was identified and named as gamahorin C (**5**).

Compound **6** was isolated as a yellow crystalline solid, with its molecular formula established as C_13_H_16_O_7_ based on HRESIMS data. The one-dimensional NMR (Table 2) and HSQC spectra displayed characteristic signals, including one methyl group [*δ*_C/H_ 13.6/2.37 (CH_3_-4′)], one methoxy group [*δ*_C/H_ 57.3/3.90 (7-OCH_3_)], two oxygenated methylene groups [*δ*_C/H_ 64.2/3.59 (CH_2_-1″), *δ*_C/H_ 64.2/3.51 (CH_2_-3″)], three olefinic methine groups [*δ*_C/H_ 91.3/5.74 (CH-3), *δ*_C/H_ 104.0/6.55 (CH-5), *δ*_C/H_ 120.8/6.65 (CH-2′)], one oxygenated secondary methine group [*δ*_C/H_ 73.9/3.65 (CH-2″)], and five sp^2^ carbon signals [*δ*_C_ 165.6 (C-2), 172.9 (C-4), 160.1 (C-6), 144.0 (C-1′), 167.9 (C-3′)]. The NMR spectra of **6** showed a strong resemblance to those of pestalotiopyrone I [19], suggesting that both compounds share the same planar structure. This hypothesis was further supported by key HMBC (Figure 2) correlations in **6**, including H-3/C-5, H_3_-7/C-4, H-2′/C-6 and C-4′, H_3_-4′/C-3′ and C-6, H-1″/C-3″, and H-2″/C-1″ and C-2″. A closer examination of the 1D NMR spectra of the two compounds revealed a significant difference at the C-1″, 2″, and 3″ positions. As C-2″ is the only chiral center on the side chain and is conformationally flexible, it was inferred that the absolute configuration of C-2″ in **6** is the opposite to that in pestalotiopyrone I. To verify the previous assumption and determine the absolute configuration of **6**, we first attempted to use the Mosher reaction to determine its configuration at the 2” position. However, the experiment was unsuccessful, resulting in an insufficient compound quantity to proceed with further chemical verification. To address this issue, we successfully determined the absolute configuration of **6** by combining specific rotation measurements with literature comparisons. We found that **6** shared the same planar structure with the known compound pestalotiopyrone I, with the opposite specific rotation signs. Specifically, the specific rotation of **6** was measured as [*α*${]}_{D}^{25}$ +1.6 (c 0.1, MeOH), while that of pestalotiopyrone I was [*α*${]}_{D}^{20}$ –13.1 (c 0.2, MeOH). Based on this observation and the literature support, we concluded that the absolute configuration of compound **6** at the 2″ position was *S*. Consequently, **6** was identified as epi-pestalotiopyrone I (**6**).

The new compound **7** was isolated as a yellow crystalline solid, with its molecular formula established as C_12_H_20_O_5_ based on HRESIMS data. The ¹H NMR spectrum (Table 3) displayed characteristic signals, including three methyl groups [*δ*_C/H_ 11.4/0.83 (CH_3_-10), *δ*_C/H_ 11.7/0.77 (CH_3_-11), *δ*_C/H_ 22.2/0.90 (CH_3_-12)], two olefinic methine groups [*δ*_C/H_ 122.4/6.29 (CH-2), *δ*_C/H_ 155.5/7.55 (CH-3)], two methylene groups [*δ*_C/H_ 36.4/1.91, 1.65 (CH_2_-5), *δ*_C/H_ 21.3/1.55, 1.26 (CH_2_-9)], two methine groups [*δ*_C/H_ 70.2/3.54 (CH-6), *δ*_C/H_ 82.8/3.36 (CH-8)], and three quaternary carbons [*δ*_C_ 170.5 (C-1), 107.5 (C-4), 38.1 (C-7)]. The ¹H and ¹³C NMR spectral data of **7** revealed the presence of a five-membered lactone group and a long-chain structure. The planar structure was established through HMBC and COSY spectra (Figure 2). Key HMBC correlations included H-3/C-1, H-2/C-4, H-5/C-3, C-7, H-6/C-12, H-8/C-6, C-11, and H3-10/C-8. Additionally, ¹H-¹H COSY correlations between H-2/H-3, H-5/H-6, and H-9/H-8, H-10 provided further support for the proposed structure. To determine the absolute configurations at C-4, C-6, and C-8, a DP4^+^ probability analysis was performed in combination with NMR calculations using the GIAO method. The NMR chemical shifts of eight possible isomers (4R*,6R*,8R*-**7**, 4R*,6R*,8S*-**7**, 4R*,6S*,8S*-**7**, 4R*,6S*,8R*-**7**, 4S*,6R*,8R*-**7**, 4S*,6S*,8R*-**7**, 4S*,6R*,8S*-**7**, 4S*,6S*,8S*-**7**) were predicted and analyzed via DP4^+^ probability analysis. The results identified 4R*, 6S*, 8S*-**7** (Figure 5) as the most likely configuration, with a DP4^+^ probability of 99%. The experimental CD curve of **7** closely matched the calculated ECD curve, further confirming its absolute configuration. In conclusion, the absolute configuration of **7** was determined to be 4*R*, 6*S*, 8*S*-**7** (Figure 3). Consequently, **7** was identified as epiclactone C (**7**).

Compound **8** was isolated as a brown oily substance, with its molecular formula established as C_14_H_24_O_5_ based on HRESIMS data, indicating three degrees of unsaturation. The ¹H NMR spectrum (Table 3) displayed characteristic signals, including four methyl groups [*δ*_C/H_ 21.4/1.17 (CH_3_-12), *δ*_C/H_ 14.3/2.04 (CH_3_-13), *δ*_C/H_ 16.8/1.91 (CH_3_-14), *δ*_C/H_ 16.8/1.07 (CH_3_-15)], two olefinic methine groups [*δ*_C/H_ 144.8/7.21 (CH-7), *δ*_C/H_ 140.3/5.51 (CH-9)], two methylene groups [*δ*_C/H_ 64.1/3.61 (CH_2_-1), *δ*_C/H_ 66.9/4.25, 4.16 (CH_2_-3)], three methine groups [*δ*_C/H_ 71.3/3.90 (CH-2), *δ*_C/H_ 41.9/2.53 (CH-10), *δ*_C/H_ 72.5/3.58 (CH-11)], and three quaternary carbons [*δ*_C_ 170.5 (C-5), 126.4 (C-6), 133.1 (C-8)]. The ¹H and ¹³C NMR spectral data of **8** revealed the presence of an *α*, *β*-dihydroxy ester unit, similar to pestalotiopyrone I [19], suggesting that it is a long-chain compound with conjugated dienes and an ester group. The planar structure was established through HMBC and COSY spectra (Figure 2). Key HMBC correlations included H-1/C-3, H-3/C-5, H-12/C-5, C-7, H-13/C-7, C-9, H-15/C-9, C-11, and H-7/C-5, C-9. Additionally, ¹H-¹H COSY correlations between H-2/H-1, H-3, H-10/H-9, H-15 further supported the proposed structure. A comparison of the NMR data for the *α*, *β*-dihydroxy ester unit indicated strong similarity to pestalotiopyrone I, suggesting the same R-configuration at C-2. To determine the absolute configurations at C-10 and C-11, a DP4^+^ probability analysis was conducted using NMR calculations via the GIAO method. Among the four possible isomers (10R*,11R*-**8**, 10R*,11S*-**8**, 10S*,11R*-**8**, 10S*,11S*-**8**), the analysis identified 10S*, 11R*-**8** (Figure 5) as the most likely configuration, with a DP4^+^ probability of 93.53%. The experimental CD curve of **8** closely matched the calculated ECD curve, providing further confirmation of the absolute configuration. In summary, the absolute configuration of **8** was determined to be 10*S*, 11*R*-**8** (Figure 3). Consequently, **8** was identified as pestalotiopyrone M (**8**).

By comparing the 1D NMR data in detail with previously reported literature values, the identified known compounds were confirmed as gamahorin (**3**) [17], pestalotiopisorin B (**4**) [20], pestalotiopyrones J (**13**) [21], pestalotiopyrones C (**14**) [21], vermopyrone (**15**) [22], 4-methoxy-3,6-dimethylpyran-2-one (**16**) [23], butyrolactone-V (**9**) [24], butyrolactone I (**10**) [25], aspergillol A (**11**) [26], and 4-hydroxyphenethyl 3-hydroxybenzoate (**12**) [27].

### 2.2. Bioactivity Assay

**Figure 6:**
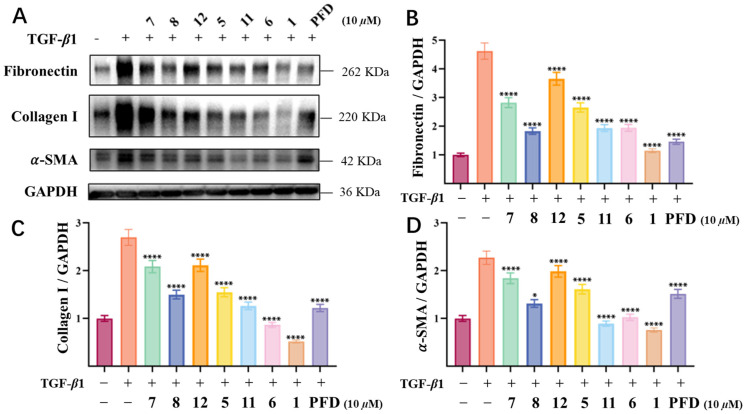
Evaluation of the renal-fibrosis-inhibitory activity of polyketides (**1**, **5**–**8**, and **11**–**12**) in a TGF-*β*1-stimulated HK-2 cell model. (**A**) The effects of polyketides (10 µM) on fibronectin, collagen I, and *α*-SMA in the cell model. (**B**–**D**) Quantification of the immunoblot data of (**A**). Data were analyzed using one-way ANOVA followed by Bonferroni’s multiple-comparisons test. * *p* < 0.05, **** *p* < 0.0001.

To investigate the therapeutic potential of the compounds in renal fibrosis, we established a TGF-*β*1-induced HK-2 cell model to simulate epithelial–mesenchymal transition (EMT) and extracellular matrix (ECM) deposition. As a result, seven tested compounds (**1**, **5**–**8**, and **11**−**12**) at 10 µM revealed obvious anti-fibrotic activity through the suppression of TGF-*β*1-induced α-smooth muscle actin (*α*-SMA) expression and ECM component production (collagen I and fibronectin) (Figure 6). Among these compounds, compound **1** was shown to be the most potent and the most promising inhibitor against renal fibrosis, being even better than the positive control pirfenidone (PFD), which is the current clinical standard for fibrosis treatment. This lead compound also demonstrated the strongest ECM-modulatory effects, significantly suppressing fibronectin and collagen I accumulation to a greater extent than all other derivatives.

Compounds **1**–**16** were also evaluated for their antibacterial and antioxidant activity. The results showed that all compounds were inactive against *Escherichia coli*, *Staphylococcus aureus*, *Streptococcus* spp., and *Salmonella* spp. Compounds **2** and **9**–**10** exhibited weak antioxidant activity, with EC_50_ values of 45.24, 37.96, and 52.53 µg/mL against DPPH radicals, respectively (positive control: ascorbic acid, 3.5 µg/mL).

## 3. Materials and Methods

### 3.1. General Experimental Procedures

The infrared spectra were recorded using a Shimadzu IR Affinity-1 Fourier transform infrared spectrophotometer (the FT-IR analysis was performed using the ATR method, with 25 scans, a resolution of 4 cm^−^¹, and a scanning range of 400–4000 cm^−^¹). Optical rotation measurements were performed with an Anton Paar MPC 500 polarimeter (Anton, Graz, Austria). UV and circular dichroism (CD) spectra were analyzed using a Chirascan circular dichroism spectrometer (Applied Photophysics, Leatherhead, U.K.). High-resolution electrospray ionization mass spectrometry (HRESIMS) data were acquired with a Bruker maxis Q-TOF mass spectrometer. Nuclear magnetic resonance (NMR) spectra were obtained on Bruker Avance-500 and Avance-700 MHz spectrometers, using tetramethylsilane (TMS) as an internal standard (Bruker BioSpin International AG, Fällanden, Switzerland); solvent peaks of methanol-*d_4_* (*δ*_H_ 3.31 and *δ*_C_ 49.0) and DMSO-*d_6_* (*δ*_H_ 2.50 and *δ*_C_ 39.52) were used as references. Semi-preparative high-performance liquid chromatography (HPLC) was carried out on a Hitachi Primaide system equipped with a diode-array detector (DAD) and an octadecylsilane (ODS) column (YMC-pack ODS-A, YMC Co. Ltd., Kyoto, Japan, 10 mm × 250 mm, 5 *μ*m). For column chromatography (CC), silica gel (200−300 mesh) (Qingdao Ocean Chemical Plant, Qingdao, China) and ODS (50 *μ*m) (Merck, Darmstadt, Germany) were utilized. Thin-layer chromatography (TLC) spots were visualized under 254 nm UV light on plates from the Qingdao Ocean Chemical Factory. Additionally, semi-preparative HPLC employed an ODS column (YMC-pack ODS-A, YMC Co. Ltd., Kyoto, Japan, 10 mm × 250 mm, 5 μm) operating at a flow rate of 2.5 mL/min.

### 3.2. Fungal Material

Strain SCSIO 41422 was obtained from an endophytic fungus associated with a sponge sample collected in the waters surrounding Weizhou Island, in the Beibu Gulf of the South China Sea. The sequence analysis of the internal transcribed spacer (ITS) region of its rDNA (GenBank accession number: NR_OQ120420) confirmed that this fungal strain belonged to the genus *Neopestalotiopsis*. The strain has been preserved at the Key Laboratory of Tropical Marine Biological Resources and Ecology, South China Sea Institute of Oceanology, Chinese Academy of Sciences, located in Guangzhou, China.

### 3.3. Fermentation and Extraction

The fungal strain *Neopestalotiopsis* sp. SCSIO 41422 was initially cultivated under static conditions on MB solid medium before being transferred to seed medium (250 mL; 1.5% malt extract, 2.4% sea salt) in 1 L Erlenmeyer flasks. The seed cultures were incubated at 28 °C on a rotary shaker set to 180 rpm for three days. For large-scale fermentation, the fungus was grown for 30 days at 26 °C under static conditions using a rice-based medium, which consisted of 200 g of rice, 2.4% sea salt, and 250 mL of water per flask. A total of 300 Erlenmeyer flasks (1 L each) were utilized for this process. The entire fermentation culture was extracted three times with ethyl acetate, producing 288 g of a brown, oily crude extract.

### 3.4. Isolation and Purification

The ethyl acetate extract was fractionated through silica gel medium-pressure liquid chromatography (MPLC) using a stepwise gradient elution. Initially, petroleum ether (PE) and dichloromethane (DCM) mixtures were applied as eluents in varying volume ratios of 1:0, 3:1, 2:1, 1:1, and 0:1. Subsequently, gradient elution with dichloromethane and methanol (DCM-CH_3_OH) was conducted, incorporating methanol concentrations of 1%, 3%, 5%, 10%, 50%, and 70%. Thin-layer chromatography (TLC) analysis guided the combination of the eluates, resulting in 16 fractions (designated as Frs. 1–16). Frs. 3–7 were subjected to fractionation using an ODS silica gel column with methanol–water (CH_3_OH/H_2_O) gradient elution ranging from 5% to 100%, resulting in 16 subfractions (Frs. A-1–A-16). Fr. A-3 was purified by semi-preparative HPLC (50% CH_3_OH/H_2_O, flow rate of 3 mL/min), yielding **1** (1.9 mg, *t_R_* = 26 min). Similarly, Fr. A-4 underwent semi-preparative HPLC purification (35% CH_3_OH/H_2_O, flow rate of 3 mL/min) to produce **13** (4.4 mg, *t*_R_ = 12 min). Fr. A-4-1 was further purified via HPLC (35% CH_3_OH/H_2_O, flow rate of 3 mL/min), yielding **3** (5.4 mg, *t*_R_ = 5 min), while Fr. A-4-4 was processed by HPLC (26% CH_3_CN/H_2_O, flow rate of 3 mL/min), isolating **14** (93.1 mg, *t*_R_ = 10 min). The further purification of Fr. A-5 by HPLC (23% CH_3_CN/H_2_O, flow rate of 3 mL/min) yielded **5** (1.48 mg, *t*_R_ = 21 min). Fr. A-7 was processed using HPLC (69% CH_3_OH/H_2_O, flow rate of 3 mL/min), yielding **8** (6.7 mg, *t*_R_ = 11 min), while Fr. A-7-2 was purified by semi-preparative HPLC (70% CH_3_CN/H_2_O, flow rate of 3 mL/min), producing **16** (89 mg, *t*_R_ = 9 min). Fr. A-8-1 underwent HPLC separation (40% CH_3_CN/H_2_O, flow rate of 3 mL/min), yielding compound **2** (36 mg, *t*_R_ = 8 min). Finally, Fr. A-10-3 was separated by HPLC (46% CH_3_CN/H_2_O, flow rate of 3 mL/min), resulting in **4** and **15** (8.4 mg and 119.8 mg, *t*_R_ = 8 and 12 min, respectively).

Frs. 8–10 were fractionated using an ODS silica gel column with methanol–water gradient elution (5–100%), producing 14 subfractions (Frs. B-1–B-14). Fr. B-6-6 was purified via HPLC (45% CH_3_CN/H_2_O, flow rate of 3 mL/min), isolating **7** (11 mg, *t*_R_ = 12 min). Similarly, Fr. B-7 was processed using HPLC (69% CH_3_OH/H_2_O, flow rate of 3 mL/min), yielding **9** (6.72 mg, *t*_R_ = 11 min), and Fr. B-9 was purified by HPLC (65% CH_3_OH/H_2_O, flow rate of 3 mL/min), producing **10** (43.6 mg, *t*_R_ = 18 min). Lastly, Frs. 11–13 were separated using an ODS silica gel column with methanol–water gradient elution (5–100%), yielding 12 subfractions (Frs. C-1–C-12). Fr. C-2 underwent HPLC purification (30% CH_3_CN/H_2_O, flow rate of 3 mL/min), producing **11** (6 mg, *t*_R_ = 35 min). Fr. C-5 was processed by HPLC (35% CH_3_CN/H_2_O, flow rate of 3 mL/min), yielding **12** (6 mg, *t*_R_ = 7 min), while Fr. C-6-3 was purified by HPLC (15% CH_3_CN/H_2_O, flow rate of 3 mL/min), producing **6** (6 mg, *t*_R_ = 7 min).

### 3.5. Spectroscopic Data of Compounds

Gamahorin A (**1**): Brown oily substance; [*α*${]}_{D}^{25}$ –5.3 (*c* 0.1, MeOH); UV (MeOH) *λ*_max_ (log *ε*) 210 (6.09), 237 (2.33), 250 (2.46), 276 (1.93), 320 (2.26) nm; ECD (0.3 mg/mL, MeOH) *λ*max (Δ*ε*) 215 (+9.96), 233 (+0.53), 256 (+8.50) nm; IR (film) *ν*_max_ 3356, 2943, 2835, 2974, 1663, 1423, 1136, 1018, 286 cm^−1^; ^1^H and ^13^C NMR data available in Table 1; HRESIMS *m*/*z* 223.0969 [M+H]^+^ (calculated for C_12_H_15_O_4_^+^, 223.0965).

Gamahorin B (**2**): Yellow crystalline solid; [*α*${]}_{D}^{25}$ +35.5 (*c* 0.1, MeOH); UV (MeOH) *λ*_max_ (log *ε*) 204 (2.95), 220 (2.76), 256 (2.43), 349 (2.25) nm; ECD (0.3 mg/mL, MeOH) *λ*max (Δ*ε*) 215 (–5.41), 238 (+3.87), 259 (+14.98) nm; IR (film) *ν*_max_ 3612, 3566, 2362, 1921, 1867, 1649, 1556, 1458, 682 cm^−1^; ^1^H and ^13^C NMR data available in Table 1; HRESIMS *m*/*z* 223.0968 [M+H]^+^ (calculated for C_12_H_15_O_4_^+^, 223.0965).

Gamahorin C (**5**): Yellow solid; [*α*${]}_{D}^{25}$ +11.05 (*c* 0.1, MeOH); UV (MeOH) *λ*_max_ (log *ε*) 211 (3.30), 236 (2.53), 249 (2.64), 275 (2.00) nm; ECD (0.3 mg/mL, MeOH) *λ*max (Δ*ε*) 203 (+9.90), 210 (+4.73), 212 (+6.06), 252 (+4.99) nm; IR (film) *ν*_max_ 3342, 2949, 2837, 1658, 1408, 1111, 1020, 590 cm^−1^; ^1^H and ^13^C NMR data available in Table 1; HRESIMS *m*/*z* 385.1496 [M+H]^+^ (calculated for C_18_H_25_O_9_^+^, 385.1493).

*epi*-Pestalotiopyrone I (**6**): Yellow crystalline solid; [*α*${]}_{D}^{25}$ +1.6 (*c* 0.1, MeOH); UV (MeOH) *λ*_max_ (log *ε*) 212 (2.94), 232 (3.29), 259 (2.62), 215 (2.79) nm; ECD (0.3 mg/mL, MeOH) *λ*max (Δ*ε*) 204 (–2.75), 212 (–2.22), 225 (–1.44), 240 (–0.96) nm; IR (film) *ν*_max_ 3587, 3564, 2924, 2358, 1716, 1598, 1417, 1039, 667 cm^−1^; ^1^H and ^13^C NMR data available in Table 2; HRESIMS *m*/*z* 285.0964 [M+H]^+^ (calculated for C_13_H_17_O_7_^+^, 285.0969).

Epiclactone C (**7**): Yellow crystalline solid; [*α*${]}_{D}^{25}$ –52.4 (*c* 0.1, MeOH); UV (MeOH) *λ*_max_ (log *ε*) 204 (2.93), 224 (2.33), 253 (1.90) nm; ECD (0.3 mg/mL, MeOH) *λ*max (Δ*ε*) 210 (–0.78), 221 (+1.36), 248 (–9.18), 284 (–0.91) nm; IR (film) *ν*_max_ 3589, 3564, 2372, 2320, 1867, 1716, 1556, 1031, 592 cm^−1^; ^1^H and ^13^C NMR data available in Table 3; HRESIMS *m*/*z* 243.1244 [M–H]^–^ (calculated for C_12_H_19_O_5_^–^, 243.1238).

Pestalotiopyrone M (**8**): Yellow solid; [*α*${]}_{D}^{25}$ +8.74 (*c* 0.1, MeOH); UV (MeOH) *λ*_max_ (log *ε*) 234 (2.82), 267 (3.08), 317 (3.04) nm; ECD (0.3 mg/mL, MeOH) *λ*max (Δ*ε*) 258 (+3.91) nm; IR (film) *ν*_max_ 3350, 2951, 2837, 1645, 1450 1408, 1117, 1015, 673 cm^−1^; ^1^H and ^13^C NMR data available in Table 3; HRESIMS *m*/*z* 273.1701 [M+H]^+^ (calculated for C_12_H_25_O_5_^+^, 273.1697).

### 3.6. X-Ray Crystallographic Analysis

Crystallographic data for **2** were collected using an XtaLAB PRO single-crystal diffractometer with Cu K*α* radiation, following the slow evaporation of the compound in methanol. The X-ray crystal structures were solved using the *SHELXS97* program, expanded through difference Fourier methods, and refined via full-matrix least-squares calculations. Non-hydrogen atoms were subjected to anisotropic refinement, while hydrogen atoms were constrained to calculated positions. The crystallographic data for **2** have been deposited in the Cambridge Crystallographic Data Centre (CCDC).

Crystal Data for C_12_H_14_O_4_ (*M* = 222.23 g/mol): orthorhombic, space group P2_1_2_1_2_1_ (no. 19), *a* = 6.91550(10) Å, *b* = 8.21390(10) Å, *c* = 19.3752(2) Å, *V* = 1100.57(2) Å^3^, *Z* = 4, *T* = 100.00(10) K, μ (Cu K*α*) = 0.837 mm^−1^, *Dcalc* = 1.341 g/cm^3^, 10,749 reflections measured (9.128° ≤ 2Θ ≤ 148.58°), 2196 unique (*R*_int_ = 0.0314, R_sigma_ = 0.0212), which were used in all calculations. The final *R*_1_ was 0.0291 (I > 2σ(I)) and the *wR*_2_ was 0.0777 (all data) (Figure 4, Flack parameter −0.08(8) and CCDC 2429168).

### 3.7. ECD Computational and DP4+ Probability Analysis Methods

Random conformational searches for molecules **1**, **5**, and **7**−**8** were conducted using the MMFF94s molecular force field implemented in Spartan ′14 V1.1.4. The identified stable conformers were further optimized in Gaussian09 at the B3LYP/6-31G(d) level under gas-phase conditions. These optimized conformers were subsequently utilized for ECD calculations in methanol at the B3LYP/6-311G (d, p) level of theory [28]. The resulting ECD data were weighted based on the Boltzmann distribution with a half-bandwidth of 0.3 eV, processed using GaussView6.0, and the ECD curves were plotted in OriginPro 2021 following UV corrections.

First, an initial conformational search was performed using the Spartan’14 software with the MIMFF94 force field, yielding a set of low-energy conformations within a 5 kcal/mol range. Subsequently, each conformation was optimized using the TDDFT quantum chemistry method at the B3LYP/6-31G (d, p) level of theory with Gaussian 09. NMR shieldings were then calculated using the GIAO method at the same B3LYP/6-31G (d, p) level, employing the PCM solvent model. Finally, Boltzmann weights were calculated using Molclus, and a DP4^+^ probability analysis was conducted [29].

### 3.8. Antioxidant Activity Assay

The DPPH radical scavenging assay was used to assess the antioxidant capacity of the compounds. Test compounds at concentrations of 1000, 500, 100, 50, and 10 *μ*g/mL, with ascorbic acid as a positive control, were dissolved in MeOH and mixed with fresh DPPH in a methanol solution. The assay was performed in triplicate, and, after a 30 min reaction in the absence of light, the absorbance was measured at 517 nm using an Enspire Genios microplate reader. The blank solution contained only MeOH, while the control solution contained MeOH instead of the sample. The DPPH scavenging activity (%) was calculated using the formula [1 − (sample absorbance − blank absorbance)/control absorbance] × 100. EC_50_ values, representing the concentration causing 50% of the maximum effect, were determined using the Origin 2022 software [30].

### 3.9. Antimicrobial Activity Assay

Compounds were evaluated for antibacterial activity against Escherichia coli, Staphylococcus aureus, Streptococcus spp., and Salmonella spp. using a modified broth microdilution method in 96-well plates [31].

### 3.10. TGF-β1-Stimulated HK-2 Cell Model

The HK-2 cells were cultured in a 37 °C, 5% CO_2_ cell incubator, maintained with DMEM/F12 (Gibco, New York, NY, USA) supplemented with 10% fetal bovine serum (FBS) (ExCell, FSP500, Suzhou, China). HK-2 cells were inoculated for 12 h in 6-well (2 × 10^5^ cells per well) plates. Subsequently, to establish the TGF-β1-stimulated HK-2 cell model, the cells were starved in serum-free medium for 24 h and then were treated with 10 ng mL^−1^ recombinant TGF-*β*1 (R&D Systems, 240-B-002, Minneapolis, MN, USA), with or without compounds, for 48 h.

### 3.11. Western Blot (WB) Analysis

The kidney tissue, HK-2, or 293T cells were lysed in RIPA (Ncmblo, WB3100, Suzhou, China) with proteinase inhibitors (Meilunbio, MB2678-1, Dalian, China) and then centrifuged (13,000× *g*, 15 min, 4 °C). A BCA kit (Epizyme, ZJ102, Shanghai, China) was employed to measure the concentration of total protein. Quantified protein-mixed loading buffer (Epizyme, LT101S) was boiled for 10 min at 100 °C, separated by SDS-PAGE, and transferred onto PVDF membranes (Millipore, ISEQ00010, Burlington, MA, USA). After incubation with blocking buffer for 1 h at room temperature, the membranes were incubated overnight at 4 °C with antibodies specific to the indicated proteins. Then, the washed membranes were incubated with the secondary antibodies (anti-rabbit or anti-mouse IgG) for 2 h at room temperature. Western blot bands were detected by the ECL (Fdbio, FD8020, Hangzhou, China) substrates and analyzed by the Image-J 1.52a software [32].

### 3.12. Statistical Analysis

All experiments, except the in vivo mouse studies, were performed at least three times. All data are expressed as the mean ± standard deviation (SD). Statistical analysis was performed using the Prism 7 software (GraphPad, San Diego, CA, USA), including Student’s *t*-test, one-way ANOVA, and two-way ANOVA. *p* < 0.05 was considered to be significant (* *p* < 0.05, ** *p* < 0.01, *** *p* < 0.001, **** *p* < 0.0001).

## 4. Conclusions

Sixteen polyketide compounds were isolated from the sponge-associated fungus *Neopestalotiopsis* sp. SCSIO 41422, including six new compounds (**1**−**2** and **5**−**8**). Their chemical structures were fully determined through NMR and HRESIMS, with quantum chemical calculations used to assign the absolute configurations of compounds **1** and **5**−**8** and X-ray diffraction confirming the absolute configuration of compound **2**. All tested compounds (**1**, **5**–**8**, and **11**–**12**) at 10 µM exhibited significant anti-fibrotic activity by inhibiting TGF-*β*1-induced *α*-SMA expression and ECM component production (collagen I and fibronectin). Among them, gamahorin A (**1**) has been shown to be the most potent and the most promising inhibitor against renal fibrosis, being even better than the positive control PFD. This lead compound also demonstrated the strongest ECM-modulatory effects, significantly suppressing fibronectin and collagen I accumulation to a greater extent than all other derivatives. This study provides promising lead compounds for the discovery of marine-derived renal fibrosis inhibitors and candidate drugs for the prevention and treatment of chronic kidney disease.

## Figures and Tables

**Table 1:** ^1^H (700MHz) and ^13^C (175MHz) NMR data of **1**-**2** and **5** in CD_3_OD.

Pos.	1		2		5	
	*δ*_C_, Type	*δ*_H_, (*J* in Hz)	*δ*_C_, Type	*δ*_H_, (*J* in Hz)	*δ*_C_, Type	*δ*_H_, (*J* in Hz)
1	170.5, C		170.7, C		170.9, C	
3	77.6, CH	5.05, qd, (6.7, 2.0)	83, CH	4.73, q, (6.7)	82.6, CH	4.57, m
4	46.7, CH	2.89, ddd, (7.6, 5.6, 1.8)	32.7, CH	3.19, q, (6.8)	38.5, CH	2.96, p, (7.0)
4a	137.9, C		126.5, C		145.3, C	
5	120.1, CH	6.79, d, (7.5)	147, C		117.9, CH	6.90, d, (7.7)
6	138.4, CH	7.39, d, (7.5)	126.6, CH	6.95, s	137.6, CH	7.74, d, (7.7)
7	126.6, C		126, C		125.9, C	
8	161.3, C		154.7, C		160.9, C	
8a	108.2, C		107, C		108.2, C	
9	19.9, CH_3_	1.37, d, (6.7)	20.3, CH_3_	1.30, d, (6.7)	19.9, CH_3_	1.43, d, (6.5)
10	64.7, CH_2_	3.66, m	19.9, CH_3_	1.27, d, (7.1)	18.2, CH_3_	1.36, d, (6.9)
11	15.5, CH_3_	2.22, s	15.5, CH_3_	2.17, s	64.7, CH_2_	4.85, d, (12.5)4.61, d, (12.5)
13					100.0, CH	4.95, d, (3.8)
15					73.7, CH	3.44, dd, (9.7, 3.8)
16					75.1, CH	3.70, m
17					71.7, CH	3.34, m
18					73.8, CH	3.66, td, (4.9, 2.1)
19					62.5, CH_2_	3.77, dd, (11.2, 2.1)3.70, m

**Table 2:** ^1^H (500MHz) and ^13^C (125MHz) NMR data of **6** in CD_3_OD.

Pos.	6	
	*δ*_C_, Type	*δ*_H_, (*J* in Hz)
2	165.6, C	
3	91.3, CH	5.74, d, (1.9)
4	172.9, C	
5	104, CH	6.55, d, (2.1)
6	160.1, C	
7	57.3, CH_3_	3.9, s
1′	144, C	
2′	120.8, CH	6.65, d, (1.6)
3′	167.9, C	
4′	13.6, CH_3_	2.37, d, (1.1)
1″	64.4, CH_2_	3.59, dd, (11.2, 4.9)
2″	73.9, CH	3.65, m
3″	64.4, CH_2_	3.51, dd, (11.2, 6.0)

**Table 3:** ^1^H (500MHz) and ^13^C (125MHz) NMR data of **7** (DMSO-*d*_6_) and **8** (CD_3_OD).

Pos.	7		8	
	*δ*_C_, Type	*δ*_H_, (*J* in Hz)	*δ*_C_, Type	*δ*_H_, (*J* in Hz)
1	170.5, C		64.1, CH_2_	3.61, m
2	122.4, CH	6.29, d, (5.6)	71.3, CH	3.90, m
3	155.5, CH	7.55, d, (5.6)	66.9, CH_2_	4.25, dd, (11.4, 4.8)4.16, dd, (11.4, 6.1)
4	107.5, C			
5	36.4, CH_2_	1.91, m1.65, dd, (13.5, 4.9)	170.5, C	
6	70.2, CH	3.54, dd, (11.8, 4.7)	126.4, C	
7	38.1, C		144.8, CH	7.21, s
8	82.8, CH	3.36, m	133.1, C	
9	21.3, CH_2_	1.55, m1.26, m	140.3, CH	5.51, d, (10.0)
10	11.4, CH_3_	0.83, t, (7.3)	41.9, CH	2.5, m
11	11.7, CH_3_	0.77, s	72.5, CH	3.58, m
12	22.2, CH_3_	0.90, s	21.4, CH_3_	1.17, d, (6.3)
13			14.3, CH_3_	2.04, s
14			16.8, CH_3_	1.91, s
15			16.8, CH_3_	1.07, d, (6.7)

## Data Availability

Data are contained within the article and Supplementary Materials.

## Supplementary Materials

The following supporting information can be downloaded at https://www.mdpi.com/article/10.3390/md23040148/s1: Figures S1–S58: The NMR, HRESIMS, UV, and IR spectra of **1**–**2** and **5**–**8**; physicochemical data of **3**–**4** and **11**–**16**; ITS sequence data of the strain.
