# Supplementary material for: New Polyketides from a Marine Sponge-Derived Fungus, Neopestalotiopsis sp., with Anti-Renal Fibrosis Activity
Source: Mar Drugs. 2025 Mar 29;23(4):148. doi: 10.3390/md23040148 (PMC12028388; doi:10.3390/md23040148)
Supplement: Supplementary file 1 [file marinedrugs-23-00148-s001.zip › marinedrugs-3535245-supplementary.pdf]

Supporting Information

New Polyketides from a Marine Sponge-Derived Fungus *Neopestalotiopsis* sp., with Anti-Renal Fibrosis Activity

Xinlong Li [1,†], Jianglian She [2,3,4,†], Meiqun Cai [3], Xinqi Chen [2,4], Rongxiang Qiu [3,5], Xiaowei Luo [1], Yonghong Liu [1,2], Xuefeng Zhou [1,2,4,*] and Lan Tang [3,*]

[1] Guangxi Key Laboratory of Marine Drugs, Institute of Marine Drugs, Guangxi University of Chinese Medicine, Nanning 530200, China

[2] CAS Key Laboratory of Tropical Marine Bio-resources and Ecology, Guangdong Key Laboratory of Marine Materia Medica, South China Sea Institute of Oceanology, Chinese Academy of Sciences, Guangzhou 510301, China

[3] NMPA Key Laboratory for Research and Evaluation of Drug Metabolism, Guangdong Provincial Key Laboratory of New Drug Screening, School of Pharmaceutical Sciences, Southern Medical University, Guangzhou 510515, China

[4] University of Chinese Academy of Sciences, Beijing 100049, China

[5] School of Chinese Materia Medica, Guangdong Pharmaceutical University, Guangzhou 510006, China

* Correspondence: xfzhou@scsio.ac.cn (X.Z.); tl405@smu.edu.cn (L.T.)

† These authors contributed equally to this work.

Contents

Figure S1. HRESIMS spectrum of compound **1**. 4
Figure S2. ^{1}H NMR (700 MHz, CD_3OD) spectrum of compound **1**....4
Figure S3. ^{13}C NMR (700 MHz, CD_3OD) spectrum of compound **1**....5
Figure S4. ^{1}H-^{1}H COSY spectrum of compound **1**. 5
Figure S5. HSQC spectrum of compound **1**....6
Figure S6. HMBC spectrum of compound **1**....6
Figure S7. NOESY spectrum of compound **1**. 7
Figure S8. The IR spectrum of compound **1**. 7
Figure S9. The UV spectrum of compound **1.** 8
Figure S10. The CD spectrum of compound **1**....9
Figure S11. HRESIMS spectrum of compound **2.** 9
Figure S12. ^{1}H NMR (700 MHz, CD_3OD) spectrum of compound **2**....9
Figure S13. ^{13}C NMR (700 MHz, CD_3OD) spectrum of compound **2**....10
Figure S14. ^{1}H-^{1}H COSY spectrum of compound **2**. 10
Figure S15. HSQC spectrum of compound **2**....11
Figure S16. HMBC spectrum of compound **2**....11
Figure S17. The IR spectrum of compound **2**. 12
Figure S20. HRESIMS spectrum of compound **5.** 13
Figure S21. ^{1}H NMR (700 MHz, CD_3OD) spectrum of compound **5**....14
Figure S22. ^{13}C NMR (700 MHz, CD_3OD) spectrum of compound **5**....14
Figure S23. ^{1}H-^{1}H COSY spectrum of compound **5**. 15
Figure S25. HMBC spectrum of compound **5**....16
Figure S26. NOESY spectrum of compound **5**. 16
Figure S27. The IR spectrum of compound **5**. 17
Figure S28. The UV spectrum of compound **5.** 17
Figure S29. The CD spectrum of compound **5**....18
....18
Figure S30. HRESIMS spectrum of compound **6.** 18
Figure S31. ^{1}H NMR (500 MHz, CD_3OD) spectrum of compound **6**....19
Figure S32. ^{13}C NMR (500 MHz, CD_3OD) spectrum of compound **6**....19
Figure S33. ^{1}H-^{1}H COSY spectrum of compound **6**. 20
Figure S34. HSQC spectrum of compound **6**....20
Figure S35. HMBC spectrum of compound **6**....21
Figure S36. The IR spectrum of compound **6**. 21
....22
Figure S37. The UV spectrum of compound **6.** 22
Figure S38. The CD spectrum of compound **6.**....22
Figure S39. HRESIMS spectrum of compound **7.** 23
Figure S40. ^{1}H NMR (500 MHz, DMSO-d_6) spectrum of compound **7**. 23
Figure S41. ^{13}C NMR (500 MHz, DMSO-d_6) spectrum of compound **7**. 24
Figure S42. ^{1}H-^{1}H COSY spectrum of compound **7**. 24
Figure S43. HSQC spectrum of compound **7**....25

Figure S44. HMBC spectrum of compound **7**.....25
Figure S45. NOESY spectrum of compound **7**.....26
Figure S46. The IR spectrum of compound **7**.....26
Figure S47. The UV spectrum of compound **7.**.....27
Figure S48. The CD spectrum of compound **7**.....28
Figure S49. HRESIMS spectrum of compound **8.**.....28
Figure S50. ^{1}H NMR (500 MHz, CD_3OD) spectrum of compound **8**.....28
Figure S51. ^{13}C NMR (500 MHz, CD_3OD) spectrum of compound **8**.....29
Figure S52. ^{1}H-^{1}H COSY spectrum of compound **8**.....29
Figure S53. HSQC spectrum of compound **8**.....30
Figure S54. HMBC spectrum of compound **8**.....30
Figure S55. NOESY spectrum of compound **8**.....31
Figure S56. The IR spectrum of compound **8**.....31
Figure S57. The UV spectrum of compound **8.**.....32
Figure S58. The CD spectrum of compound **8**.....32
The physicochemical data of the known compounds 3-4, and 11-16.....33
The ITS gene sequences data of *Neopestalotiopsis sp.* SCSIO 41422.....35

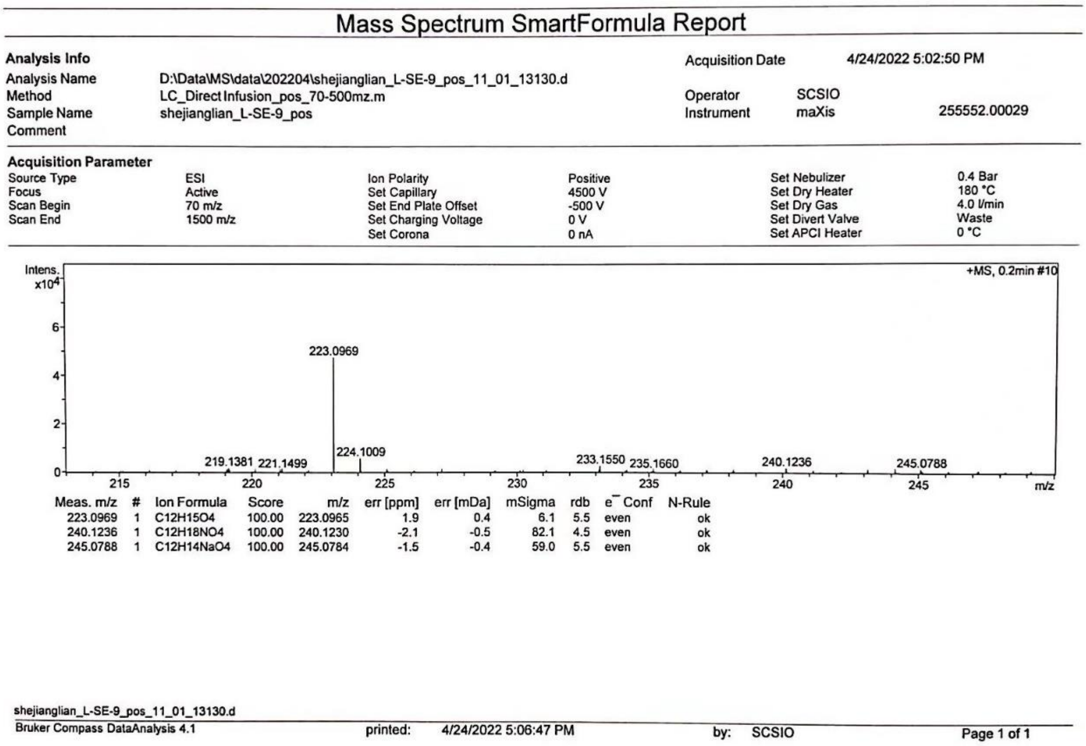

Mass Spectrum SmartFormula Report

Analysis Info			
Analysis Name	D:\Data\MS\data\202204\shejianglian_L-SE-9_pos_11_01_13130.d	Acquisition Date	4/24/2022 5:02:50 PM
Method	LC_Direct Infusion_pos_70-500mz.m	Operator	SCSIO
Sample Name	shejianglian_L-SE-9_pos	Instrument	maXis 255552.00029
Comment			

Acquisition Parameter					
Source Type	ESI	Ion Polarity	Positive	Set Nebulizer	0.4 Bar
Focus	Active	Set Capillary	4500 V	Set Dry Heater	180 °C
Scan Begin	70 m/z	Set End Plate Offset	-500 V	Set Dry Gas	4.0 l/min
Scan End	1500 m/z	Set Charging Voltage	0 V	Set Divert Valve	Waste
		Set Corona	0 nA	Set APCI Heater	0 °C

Meas. m/z	#	Ion Formula	Score	m/z	err [ppm]	err [mDa]	mSigma	rdb	e⁻ Conf	N-Rule
223.0969	1	C12H15O4	100.00	223.0965	1.9	0.4	6.1	5.5	even	ok
240.1236	1	C12H18NO4	100.00	240.1230	-2.1	-0.5	82.1	4.5	even	ok
245.0788	1	C12H14NaO4	100.00	245.0784	-1.5	-0.4	59.0	5.5	even	ok

shejianglian_L-SE-9_pos_11_01_13130.d
Bruker Compass DataAnalysis 4.1 printed: 4/24/2022 5:06:47 PM by: SCSIO Page 1 of 1

Figure S1. HRESIMS spectrum of compound 1.

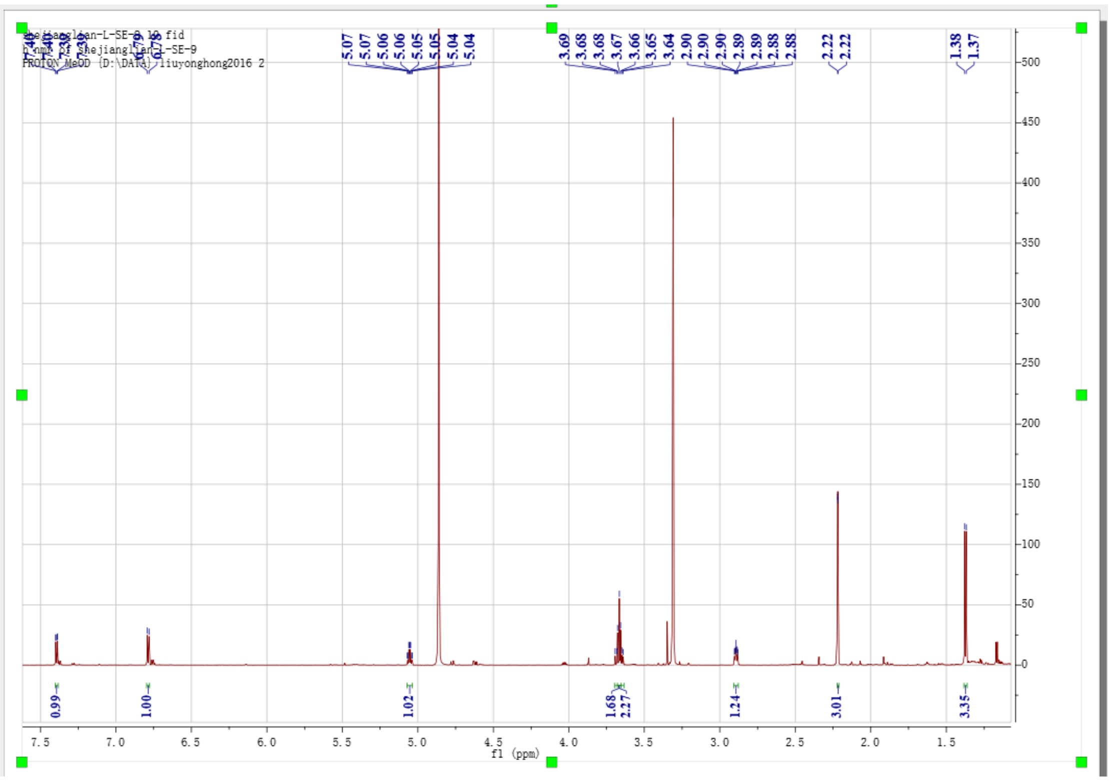

Figure S2. ^{1}H NMR (700 MHz, CD_3OD) spectrum of compound **1**.

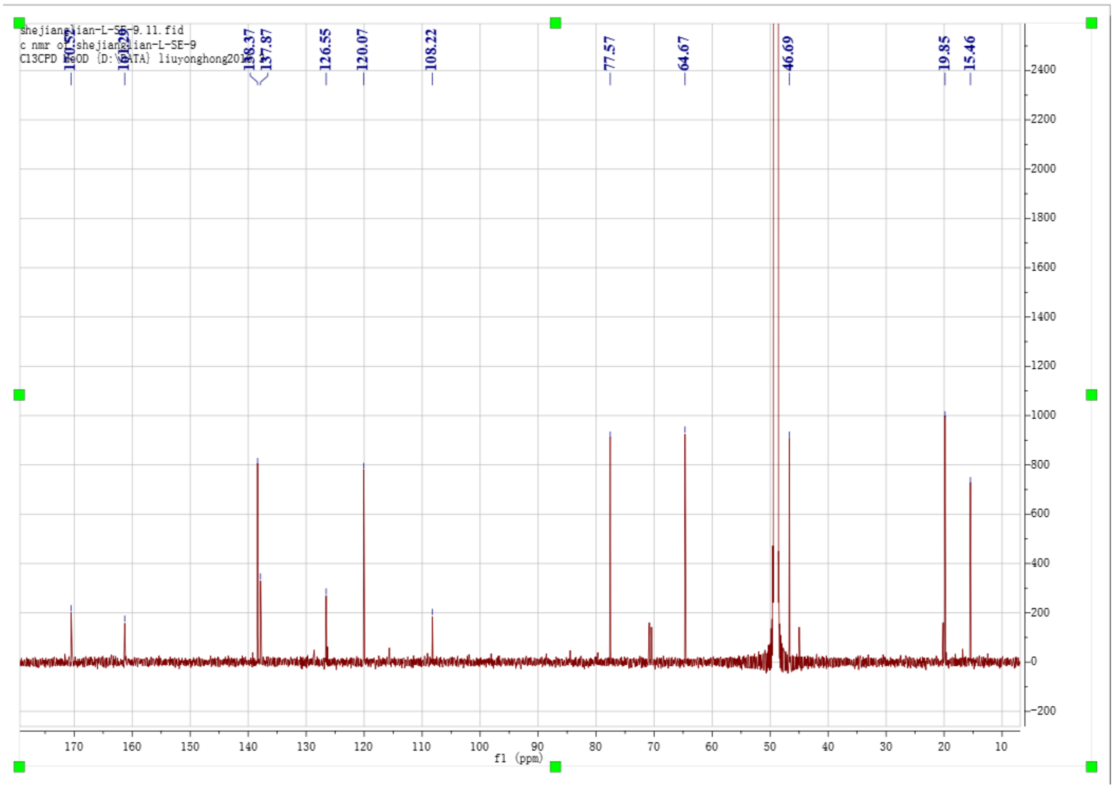

Figure S3. ^{13}C NMR (700 MHz, CD_3OD) spectrum of compound **1**.

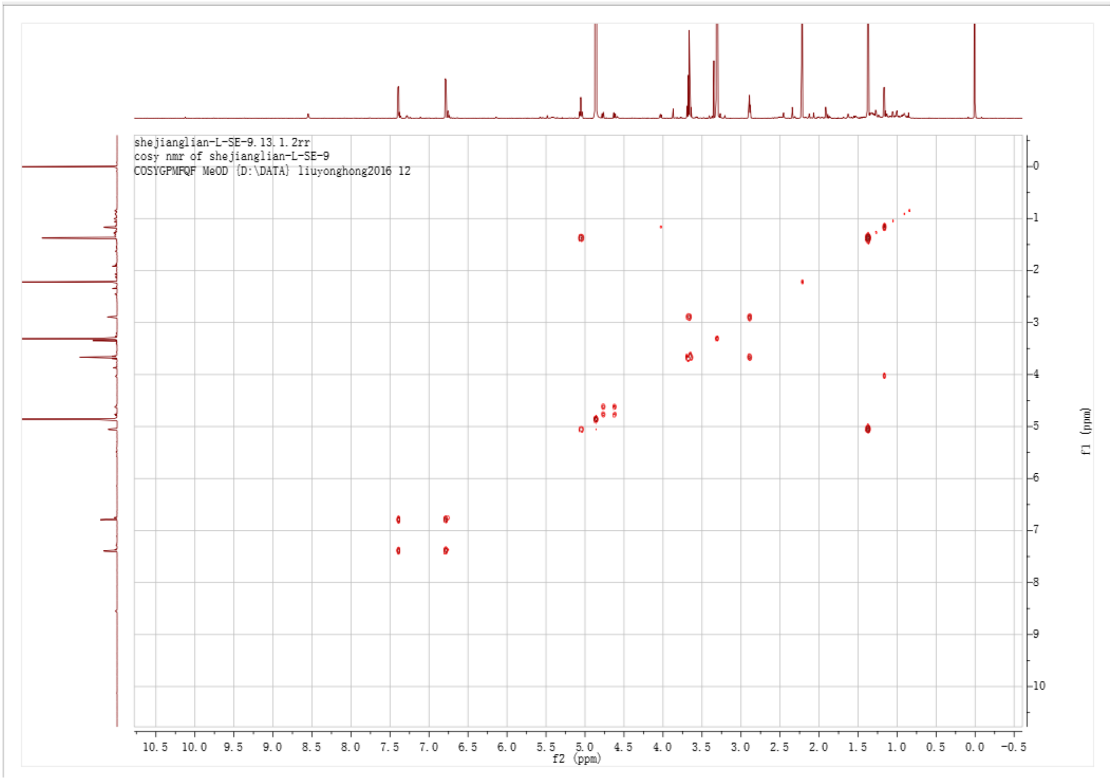

Figure S4. ^{1}H-^{1}H COSY spectrum of compound **1**.

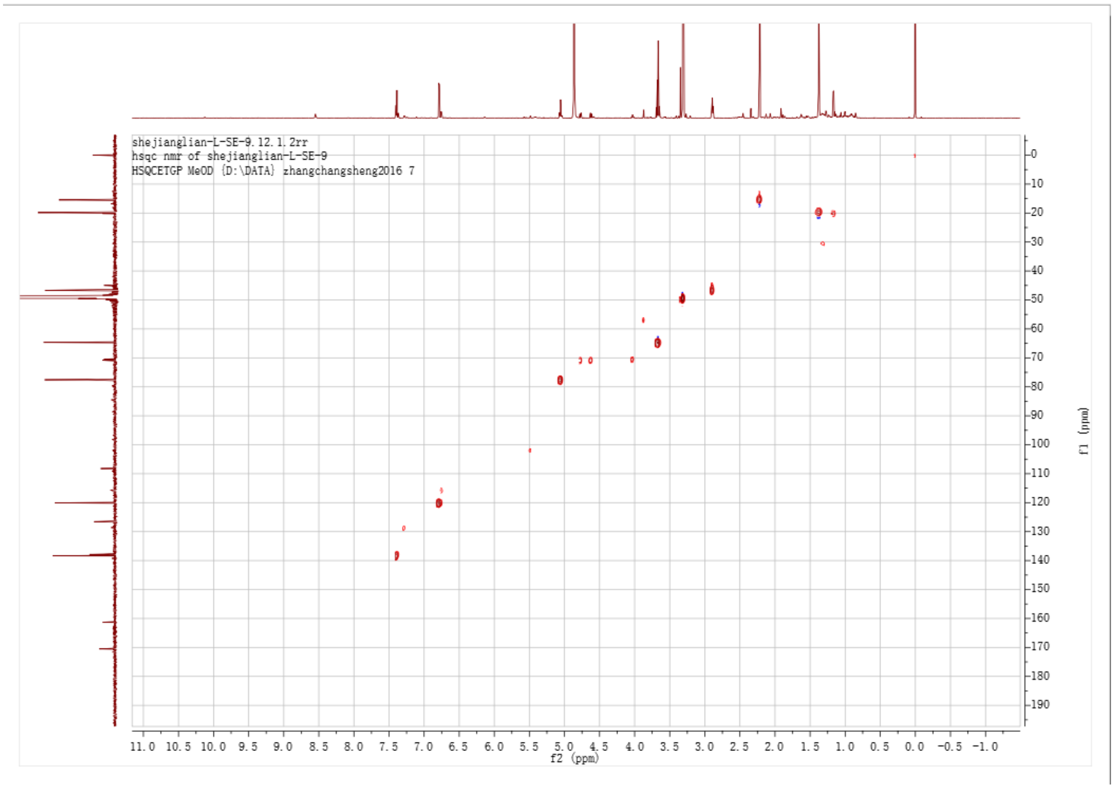

Figure S5. HSQC spectrum of compound **1**.

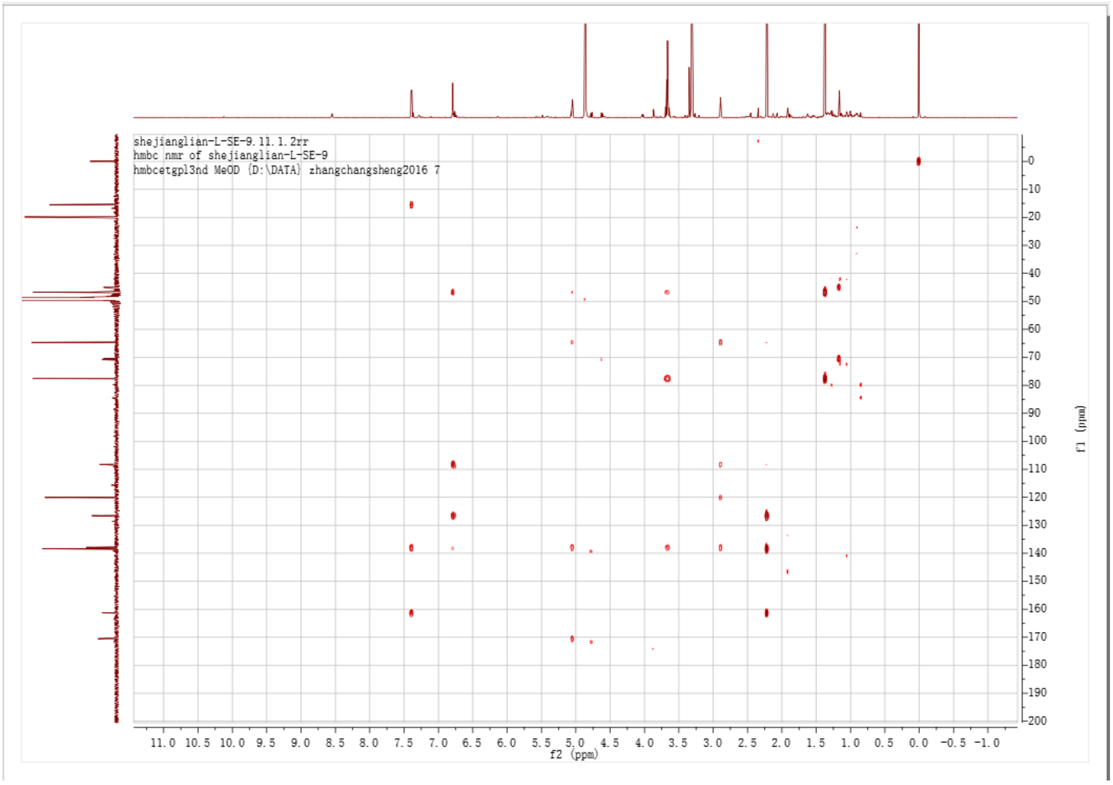

Figure S6. HMBC spectrum of compound **1**.

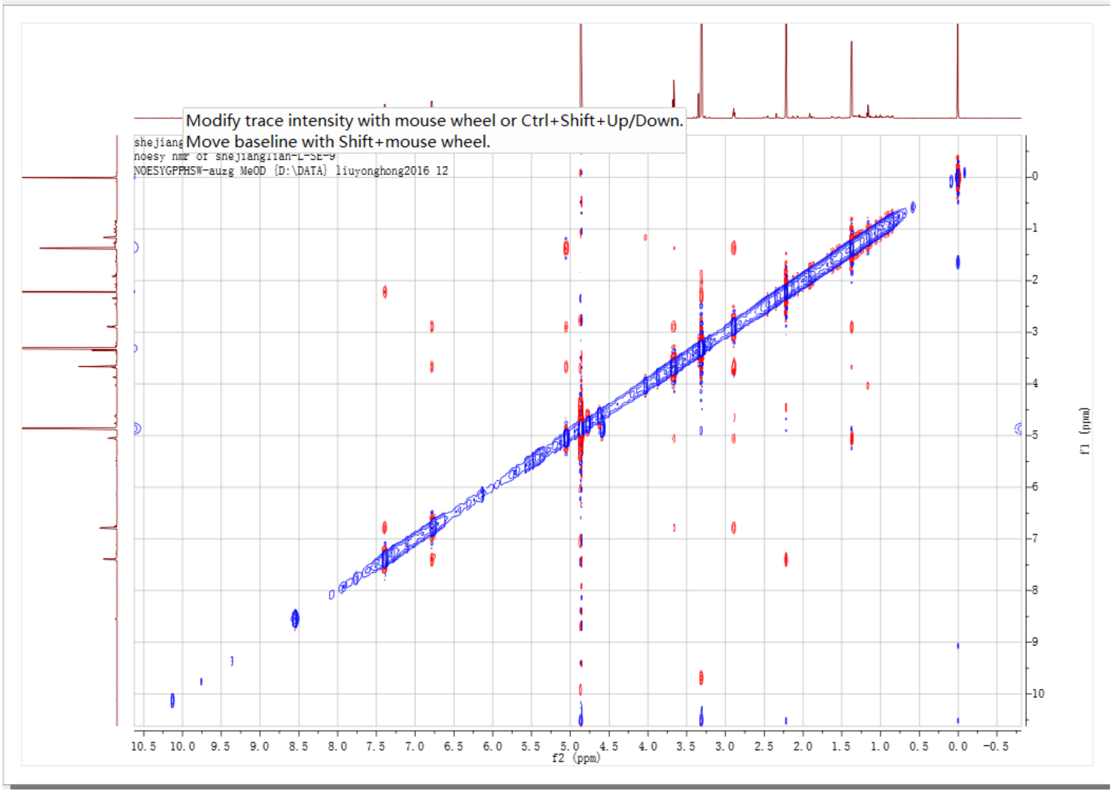

Figure S7. NOESY spectrum of compound **1**.

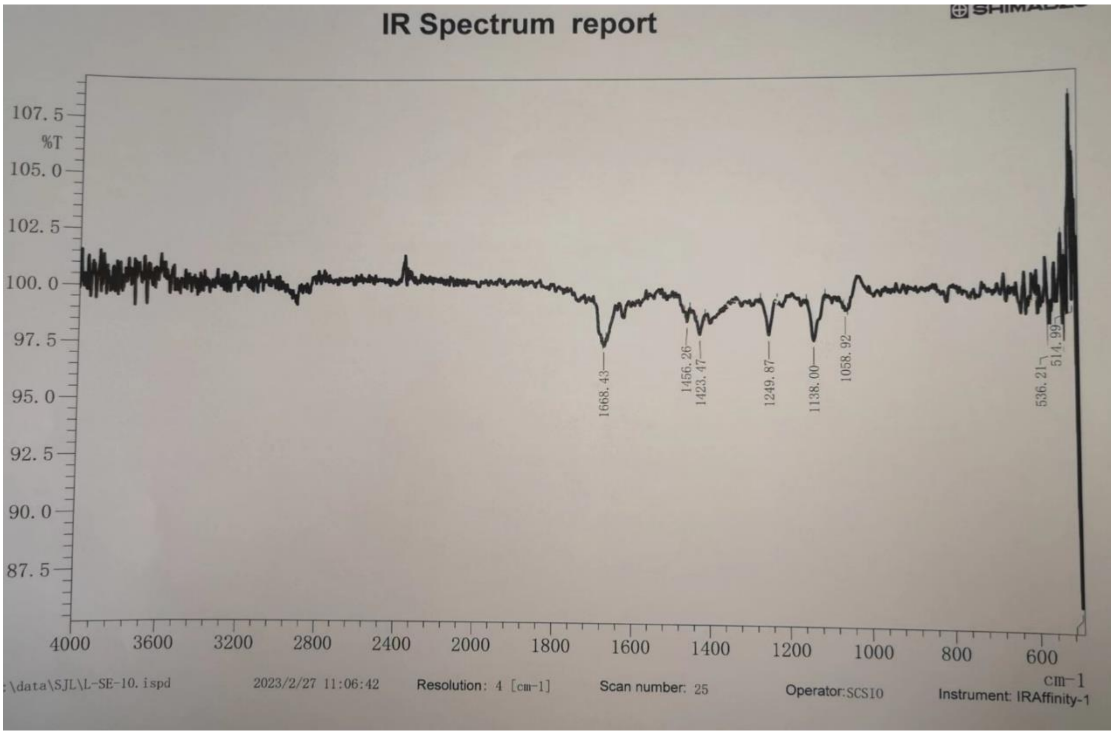

Figure S8. The IR spectrum of compound **1**.

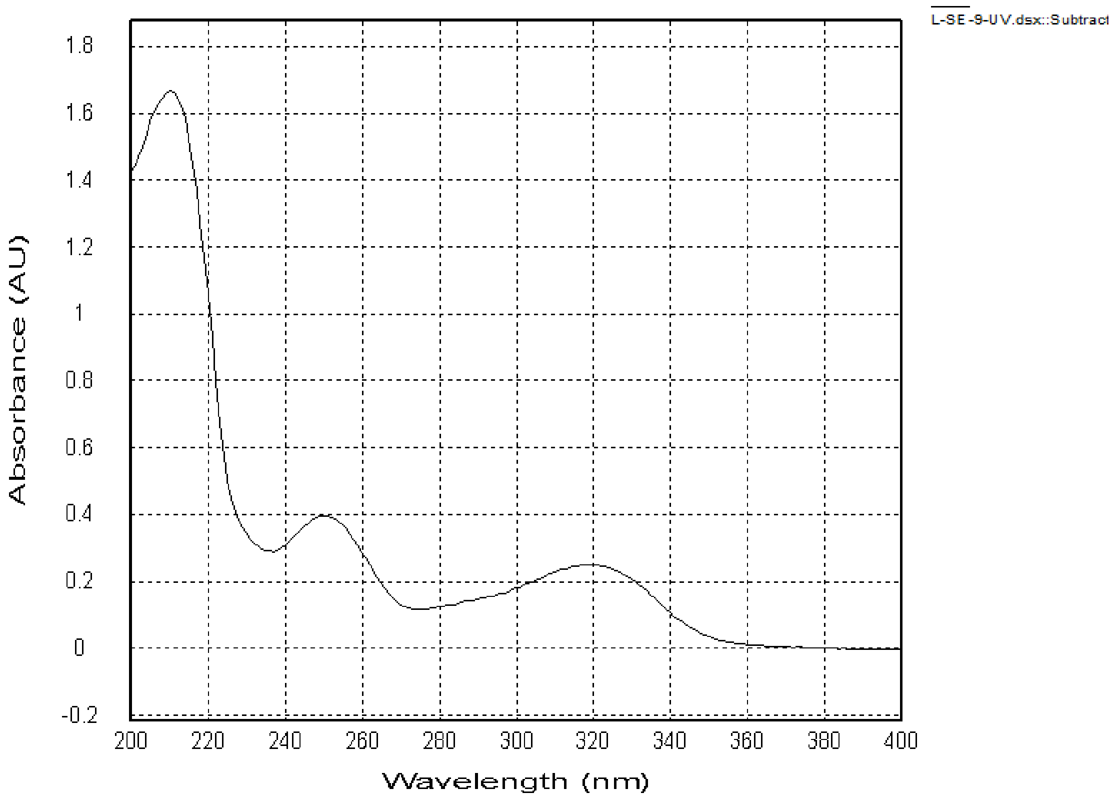

Figure S9. The UV spectrum of compound **1.**

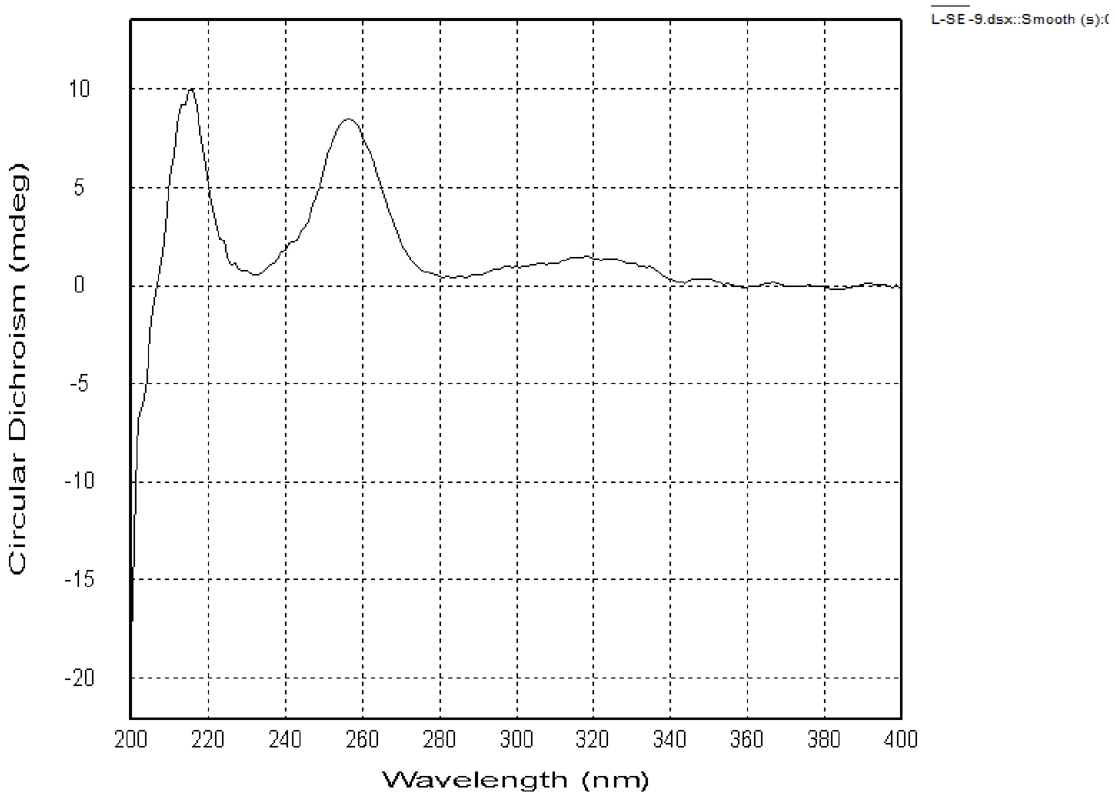

Figure S10. The CD spectrum of compound **1**.

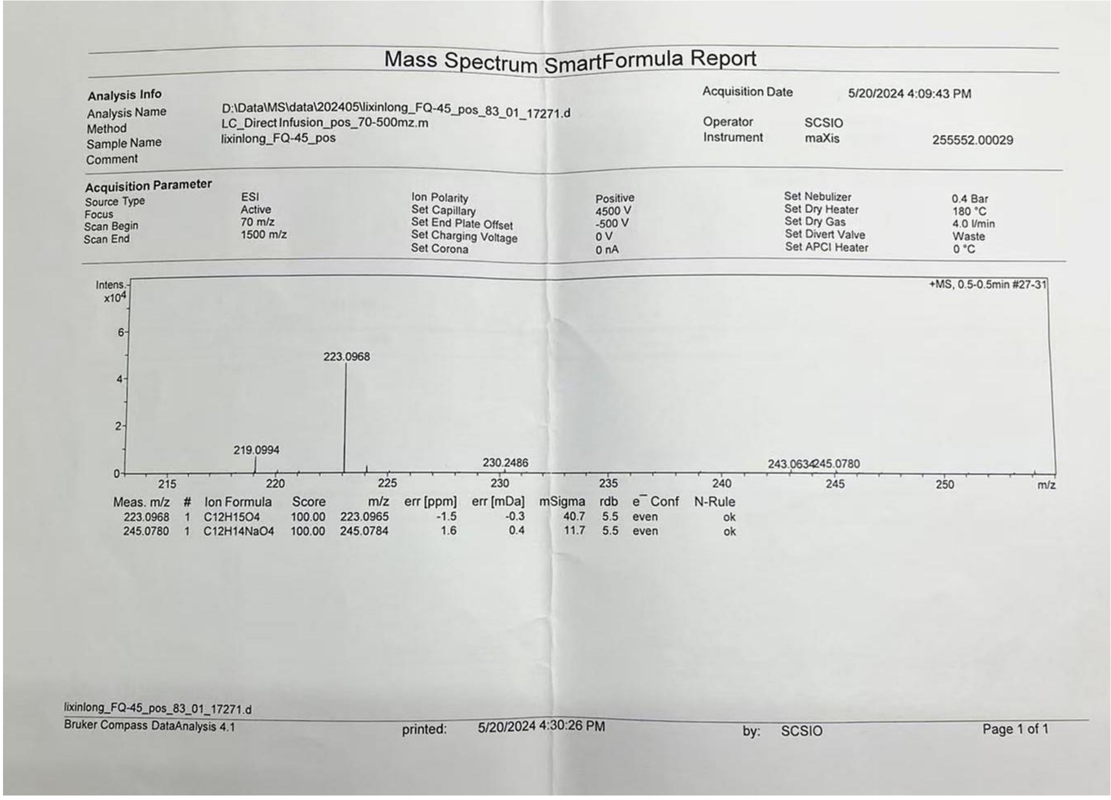

Figure S11. HRESIMS spectrum of compound **2.**

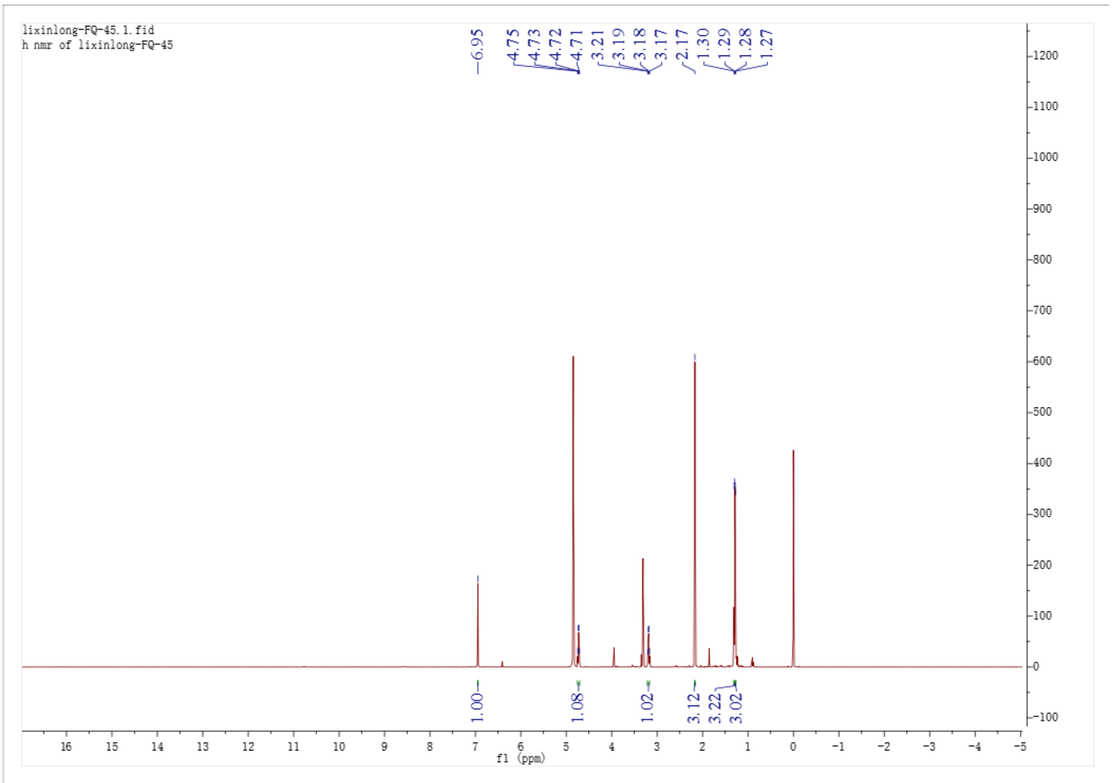

Figure S12. ^{1}H NMR (700 MHz, CD_3OD) spectrum of compound **2**.

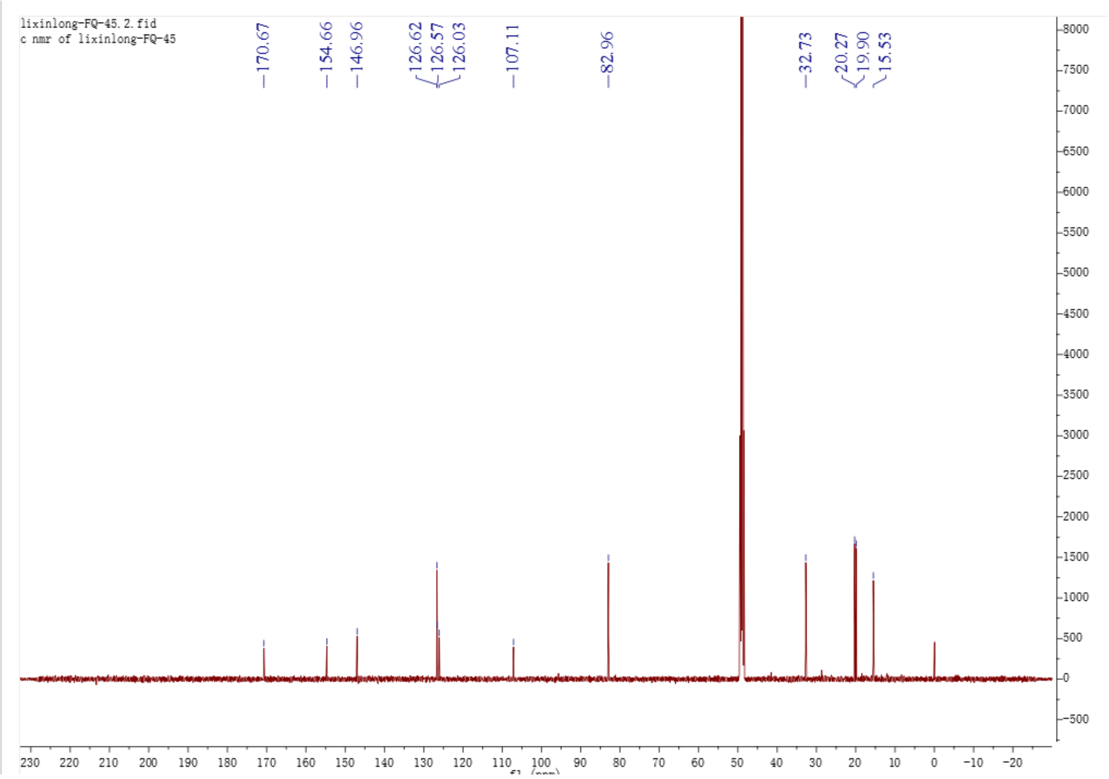

Figure S13. ^{13}C NMR (700 MHz, CD_3OD) spectrum of compound **2**.

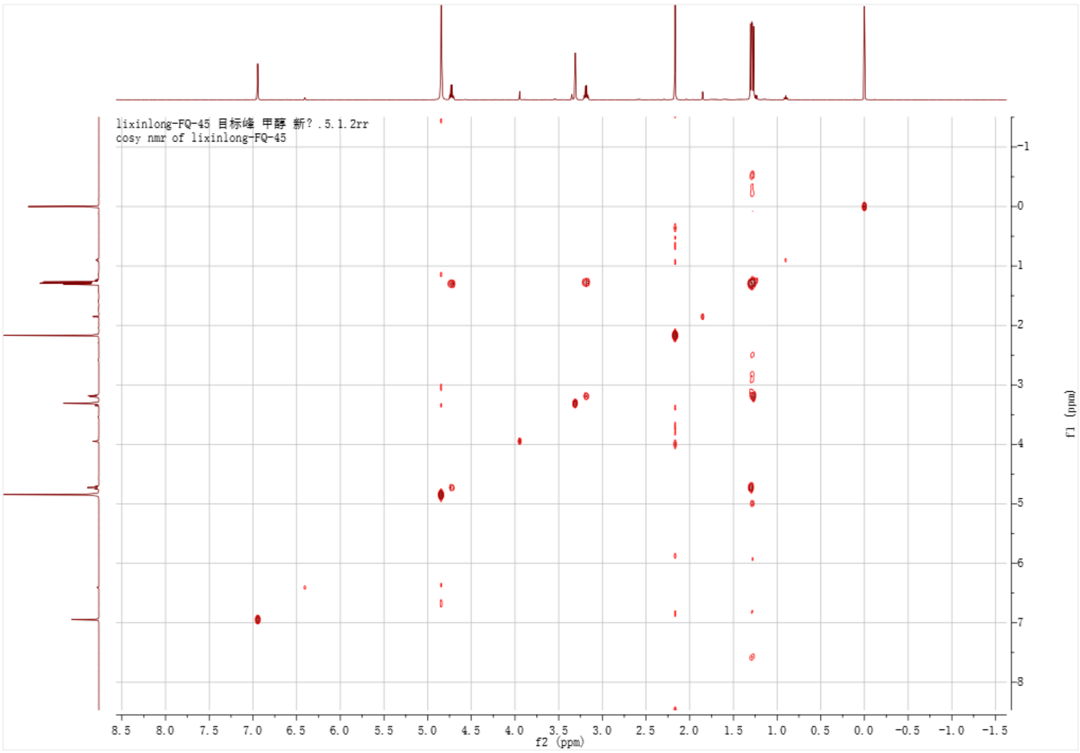

Figure S14. ^{1}H-^{1}H COSY spectrum of compound **2**.

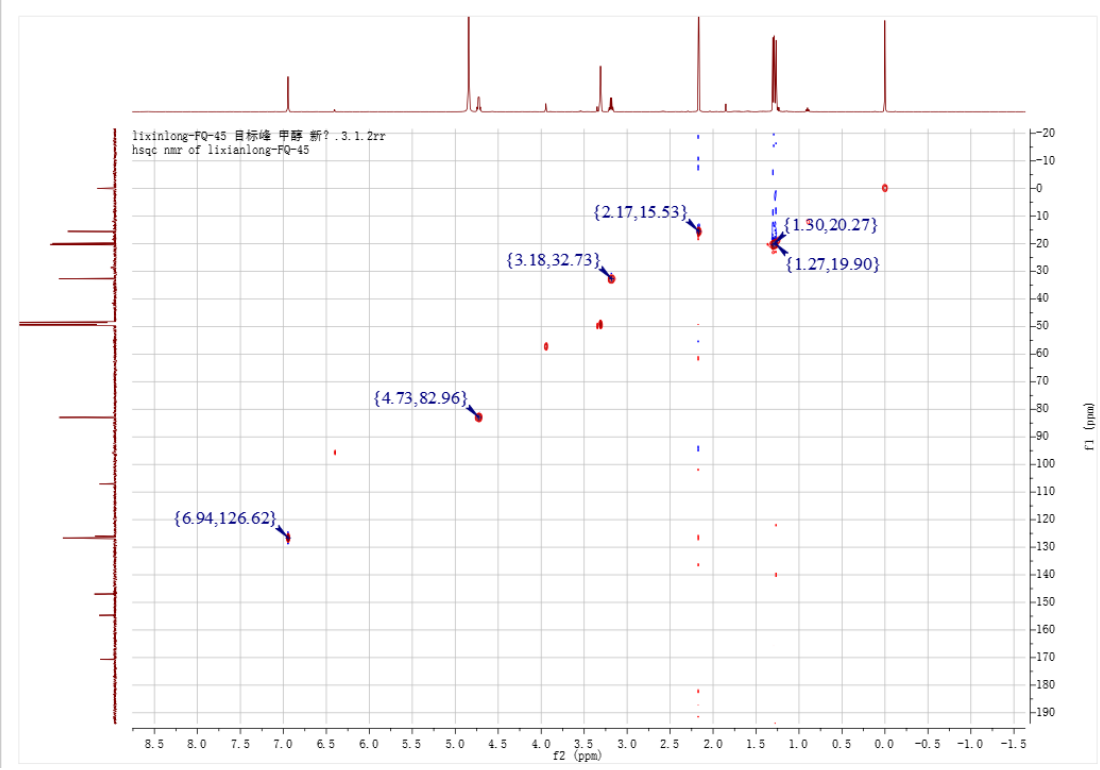

Figure S15. HSQC spectrum of compound **2**.

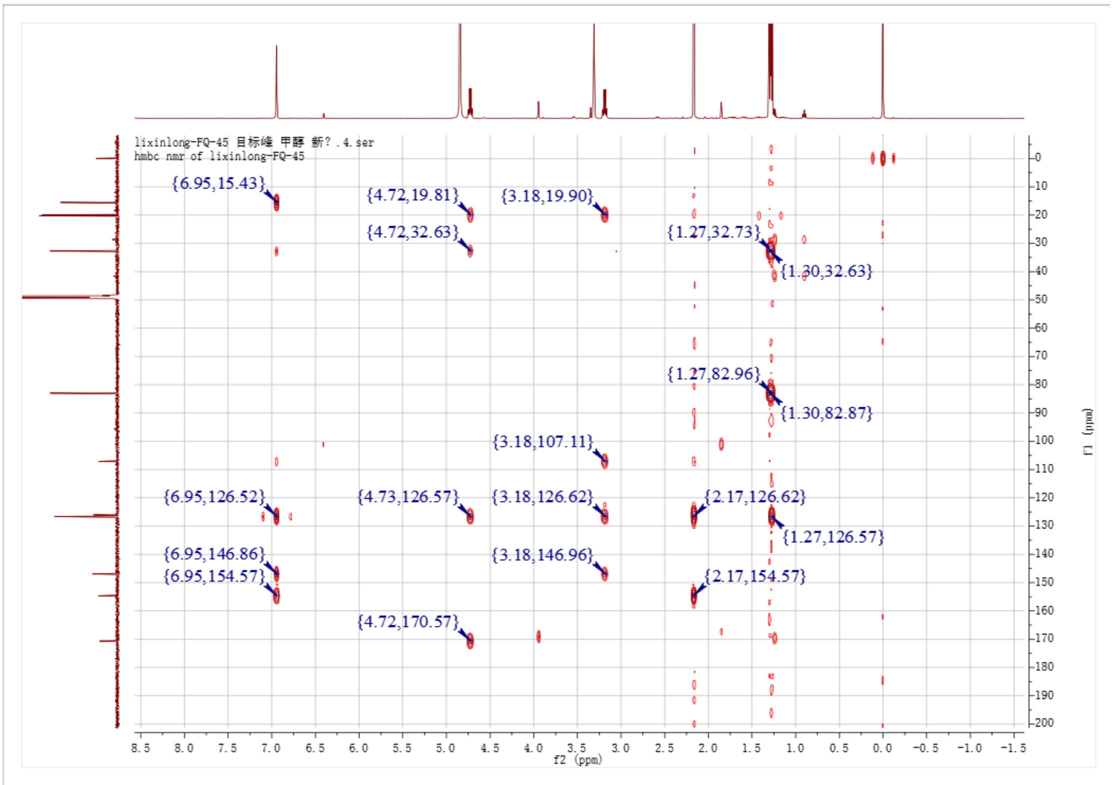

Figure S16. HMBC spectrum of compound **2**.

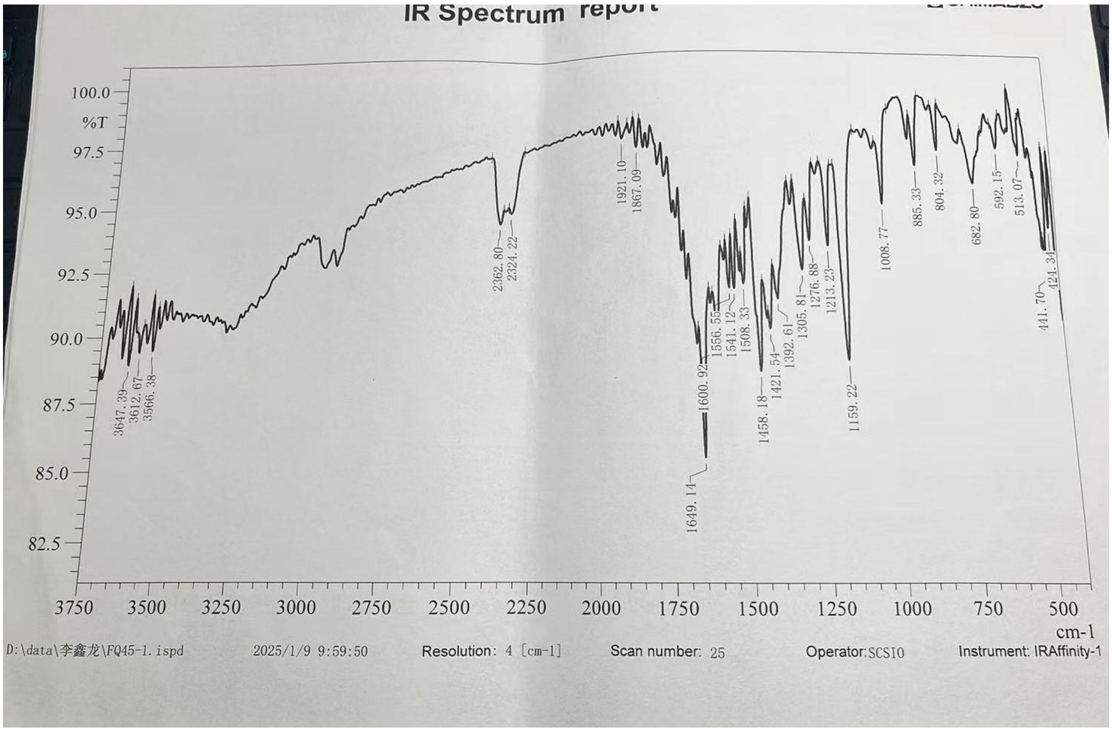

Figure S17. The IR spectrum of compound **2**.

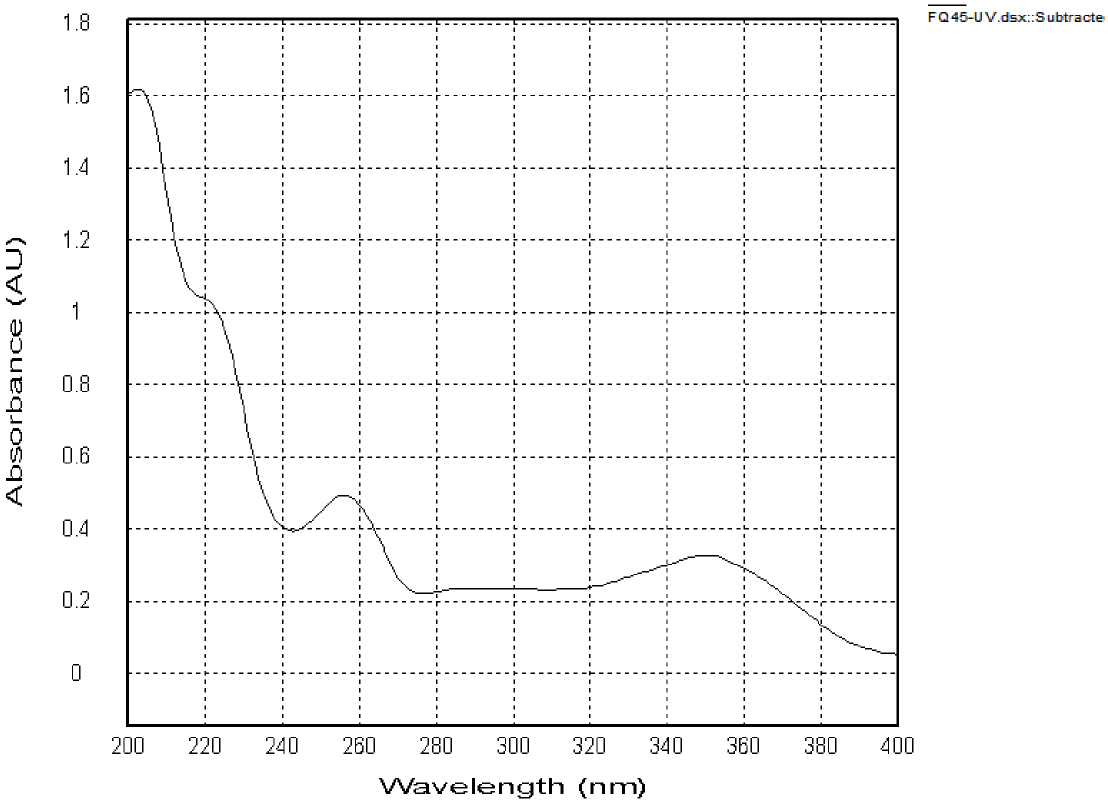

Figure S18. The UV spectrum of compound **2**.

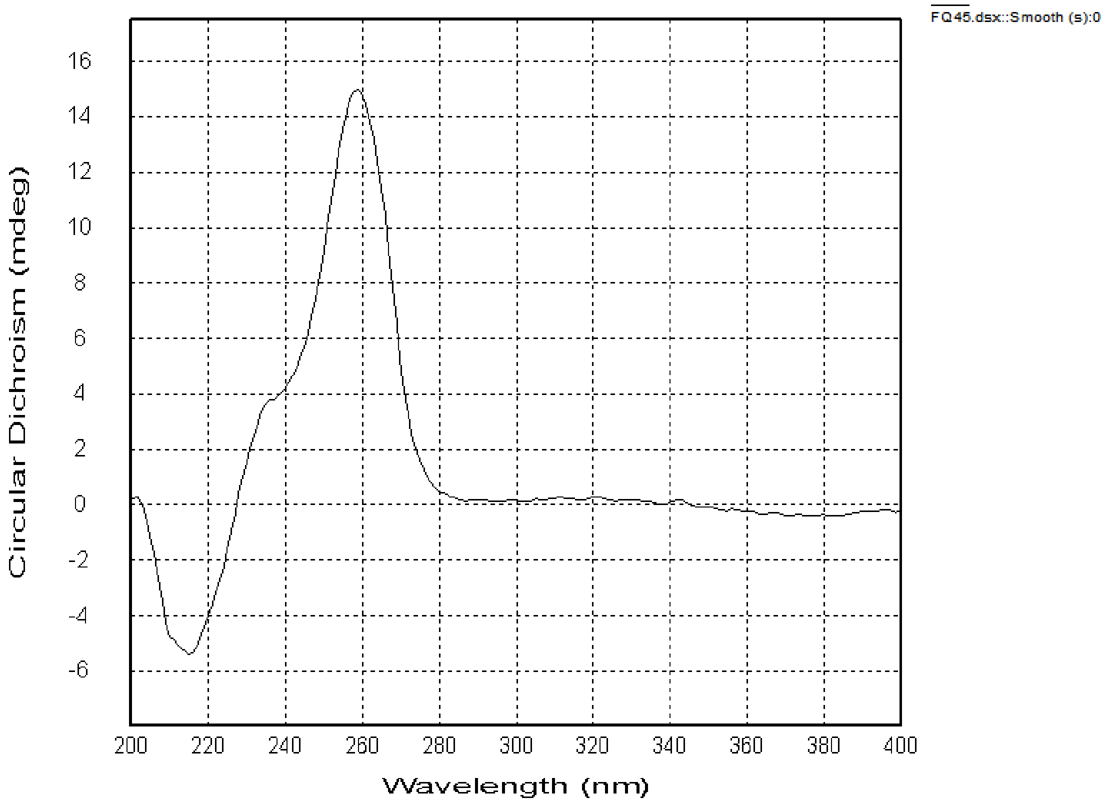

Figure S19. The CD spectrum of compound **2**.

Mass Spectrum SmartFormula Report

Analysis Info

Analysis Name	D:\Data\MS\data\202207\shejianglian_L-SE-55_pos_53_01_13270.d	Acquisition Date	7/18/2022 3:48:37 PM
Method	LC_Direct Infusion_pos_70-500mz.m	Operator	SCSIO
Sample Name	shejianglian_L-SE-55_pos	Instrument	maXis 255552.00029
Comment			

Acquisition Parameter

Source Type	ESI	Ion Polarity	Positive	Set Nebulizer	0.4 Bar
Focus	Active	Set Capillary	4500 V	Set Dry Heater	180 °C
Scan Begin	70 m/z	Set End Plate Offset	-500 V	Set Dry Gas	4.0 l/min
Scan End	1500 m/z	Set Charging Voltage	0 V	Set Divert Valve	Waste
		Set Corona	0 nA	Set APCI Heater	0 °C

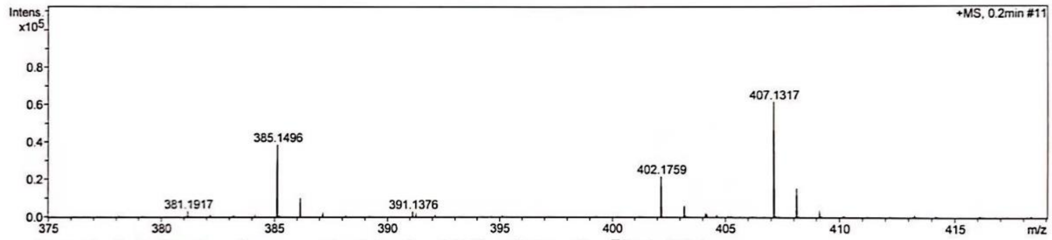

Meas. m/z	#	Ion Formula	Score	m/z	err [ppm]	err [mDa]	mSigma	rdb	e⁻ Conf	N-Rule
385.1496	1	C18H25O9	100.00	385.1493	-0.8	-0.3	30.0	6.5	even	ok
402.1759	1	C18H28NO9	100.00	402.1759	0.1	0.0	41.7	5.5	even	ok
407.1317	1	C18H24NaO9	100.00	407.1313	1.0	0.4	26.6	6.5	even	ok
786.3185	1	C36H52NO18	100.00	786.3179	-0.7	-0.6	89.7	11.5	even	ok
791.2735	1	C36H48NaO18	100.00	791.2733	0.3	0.3	42.2	12.5	even	ok

shejianglian_L-SE-55_pos_53_01_13270.d
Bruker Compass DataAnalysis 4.1 printed: 7/18/2022 3:54:36 PM by: SCSIO Page 1 of 1

Figure S20. HRESIMS spectrum of compound **5.**

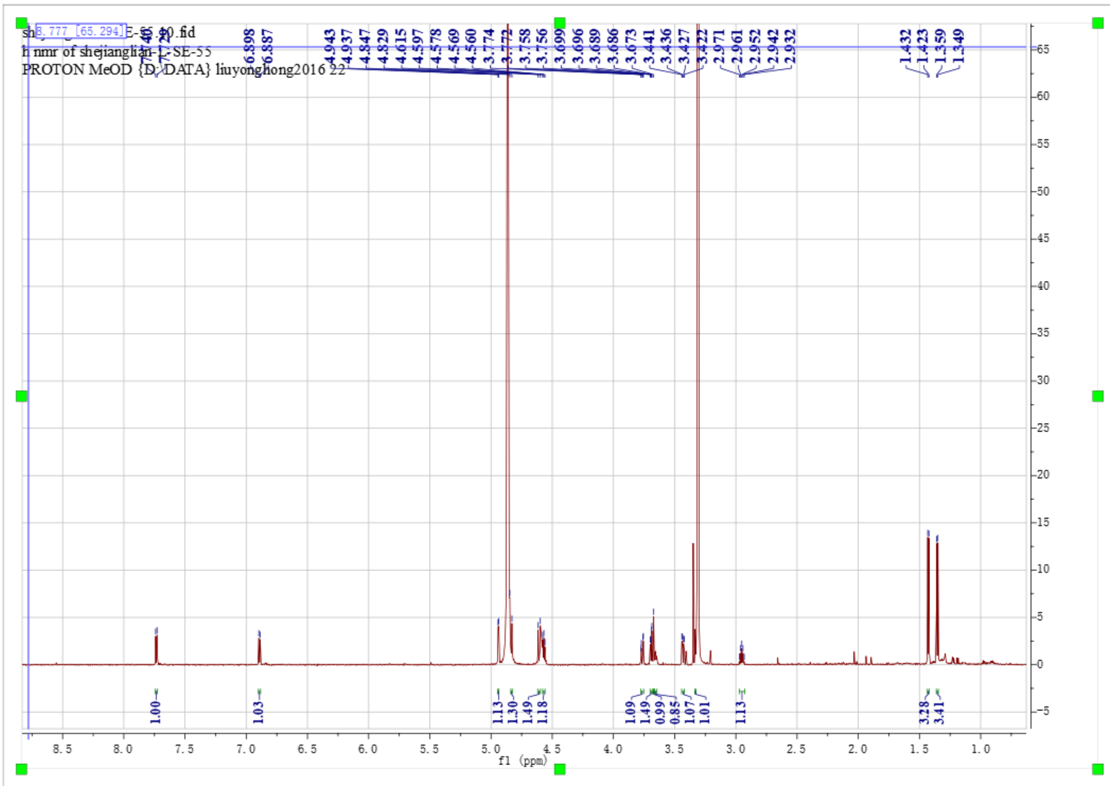

Figure S21. ^{1}H NMR (700 MHz, CD_3OD) spectrum of compound **5**.

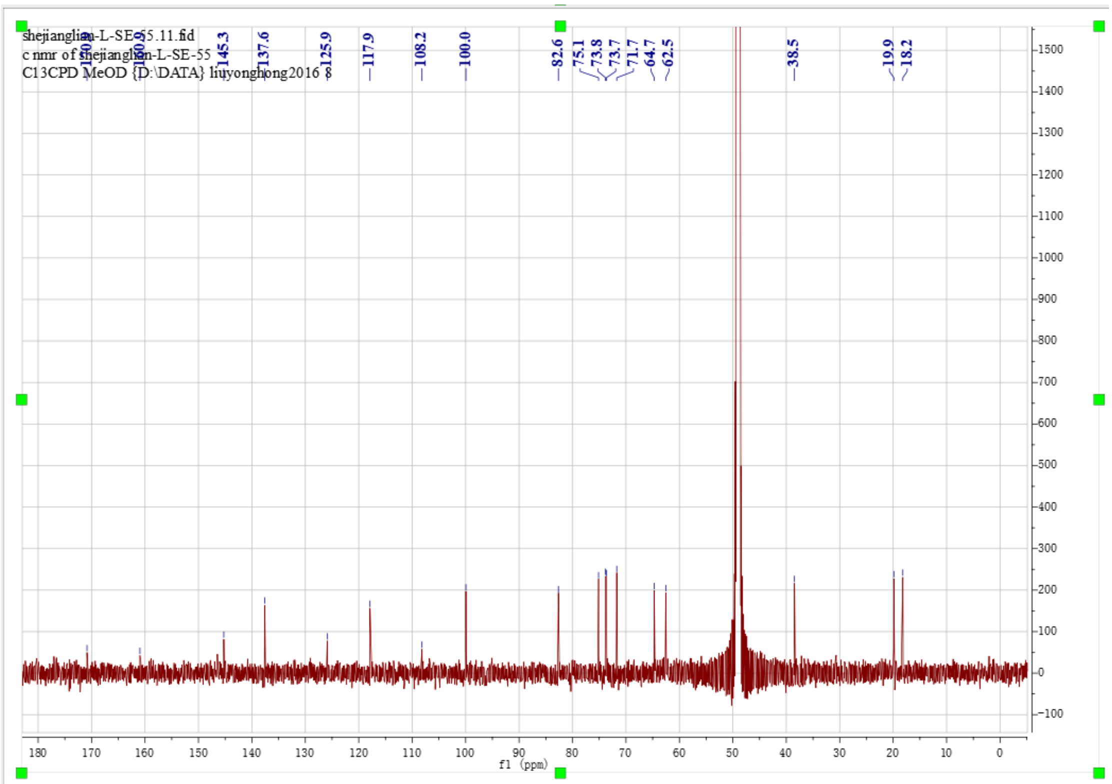

Figure S22. ^{13}C NMR (700 MHz, CD_3OD) spectrum of compound **5**.

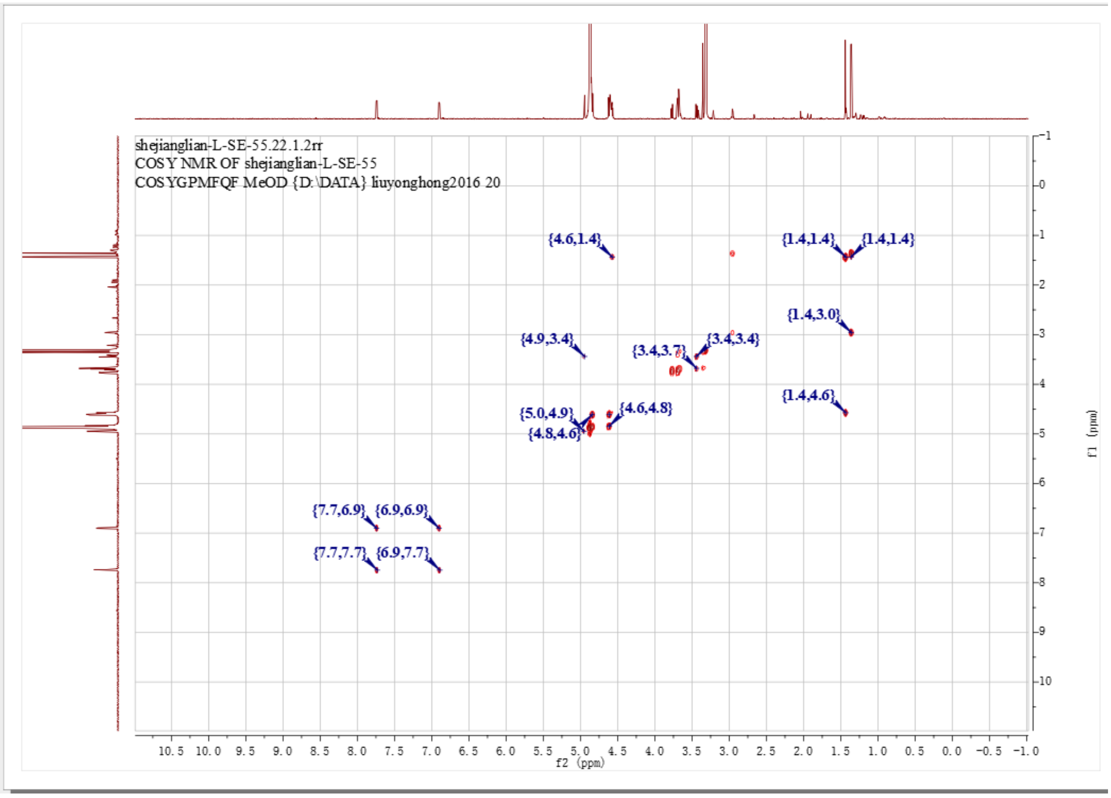

Figure S23. ^{1}H-^{1}H COSY spectrum of compound **5**.

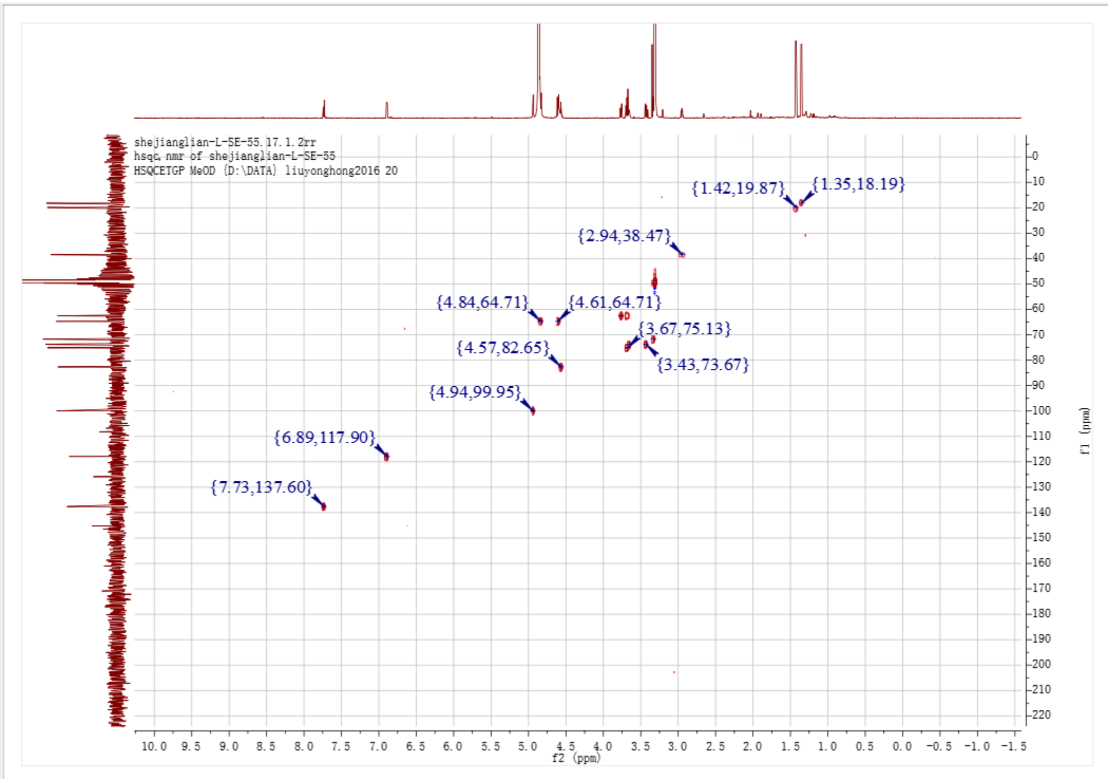

Figure S24. HSQC spectrum of compound **5**.

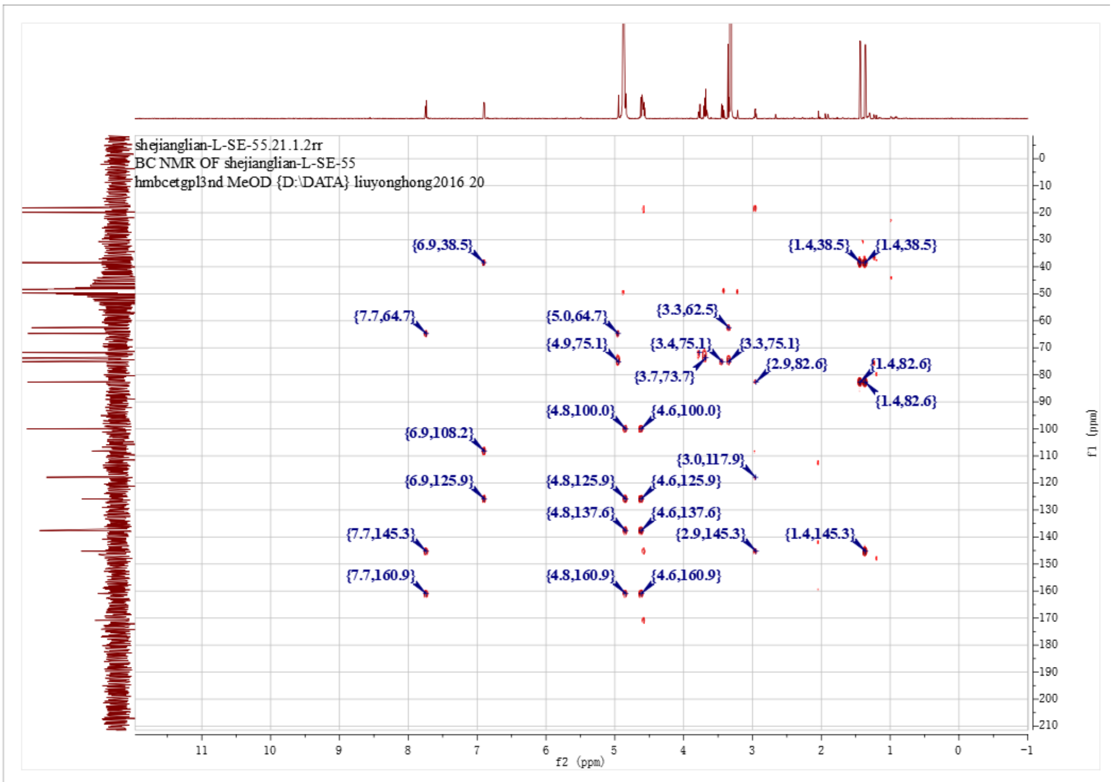

Figure S25. HMBC spectrum of compound **5**.

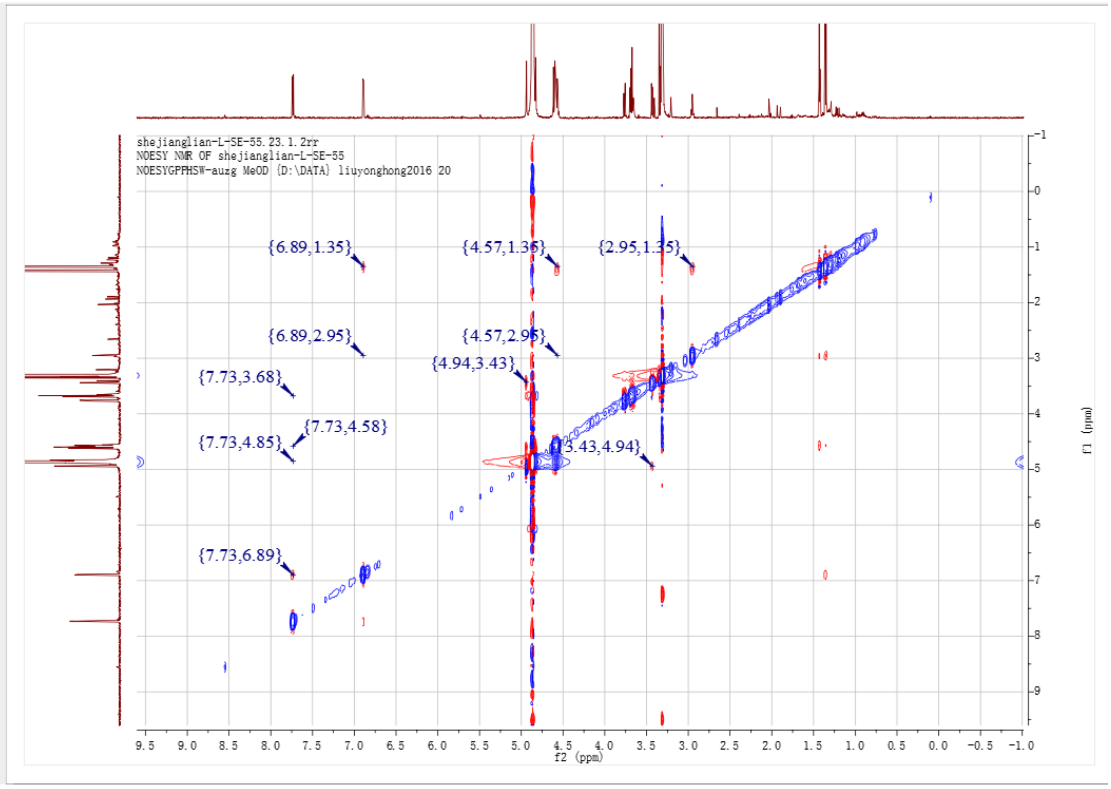

Figure S26. NOESY spectrum of compound **5**.

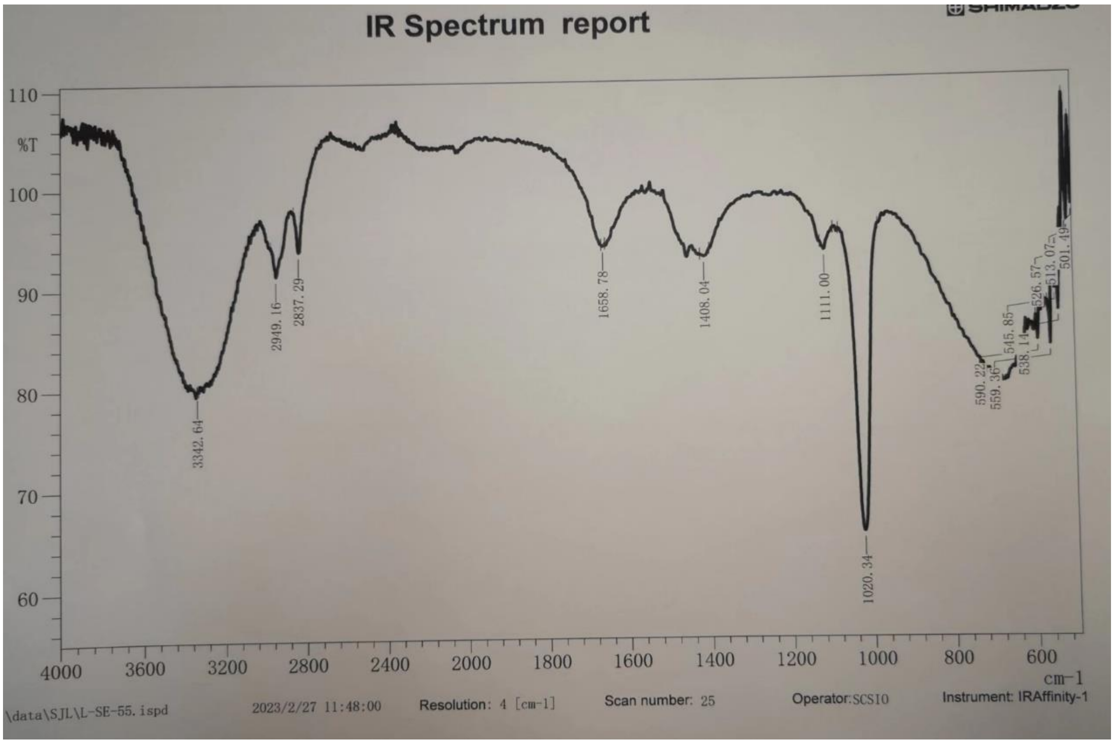

Figure S27. The IR spectrum of compound **5**.

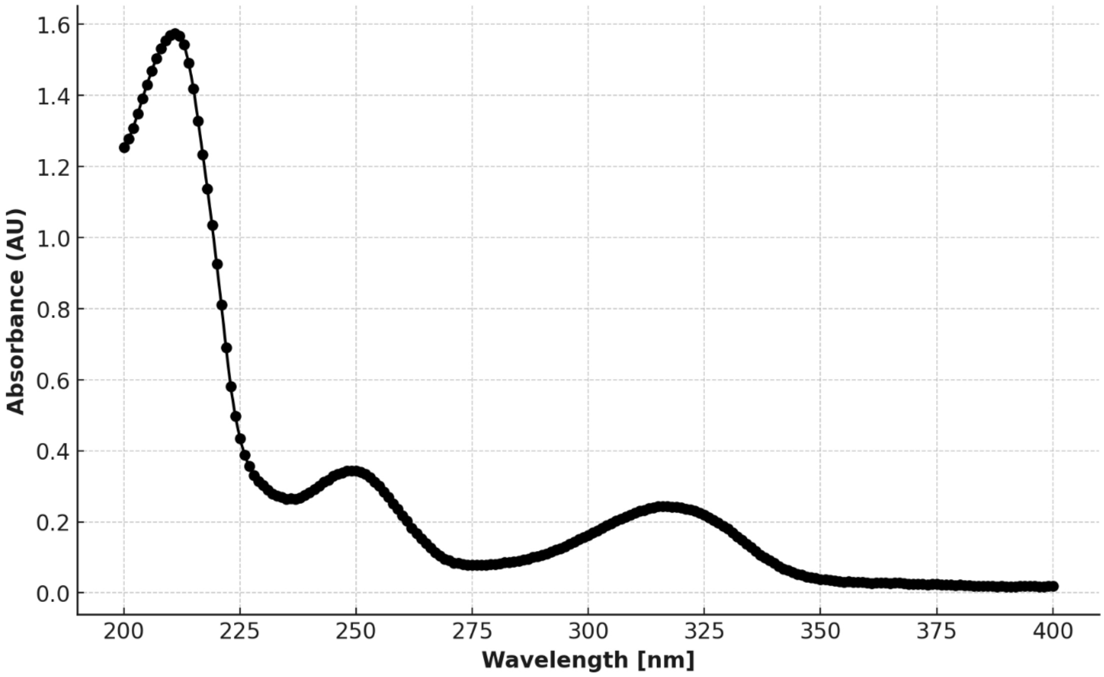

Figure S28. The UV spectrum of compound **5.**

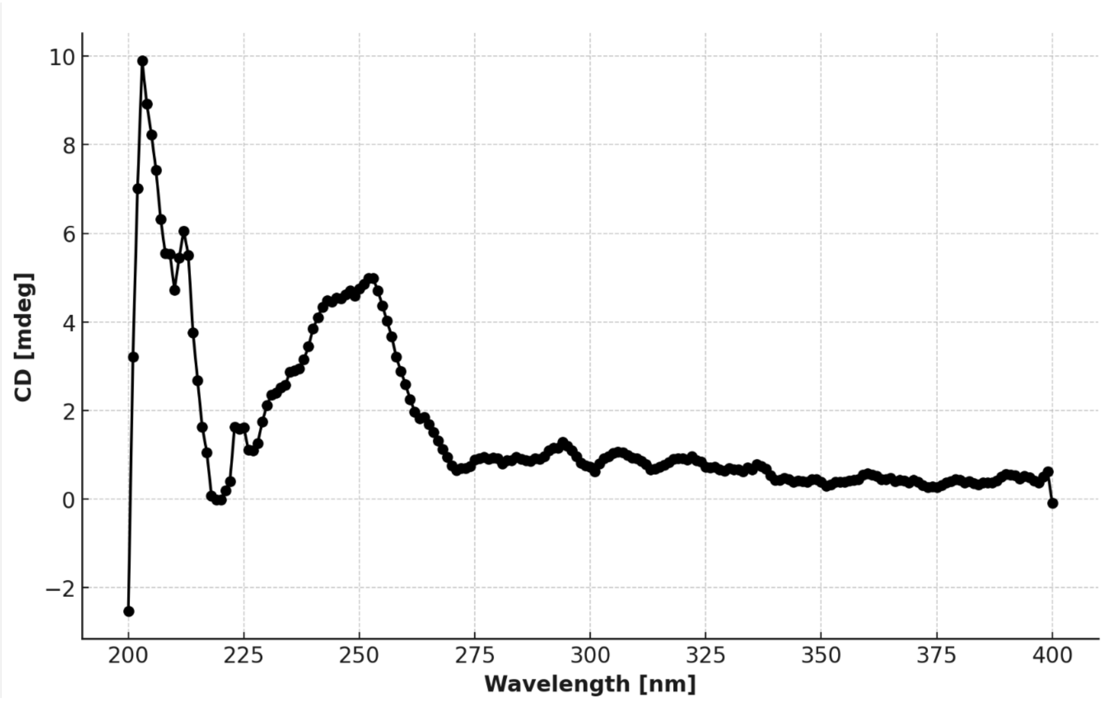

Figure S29. The CD spectrum of compound **5**.

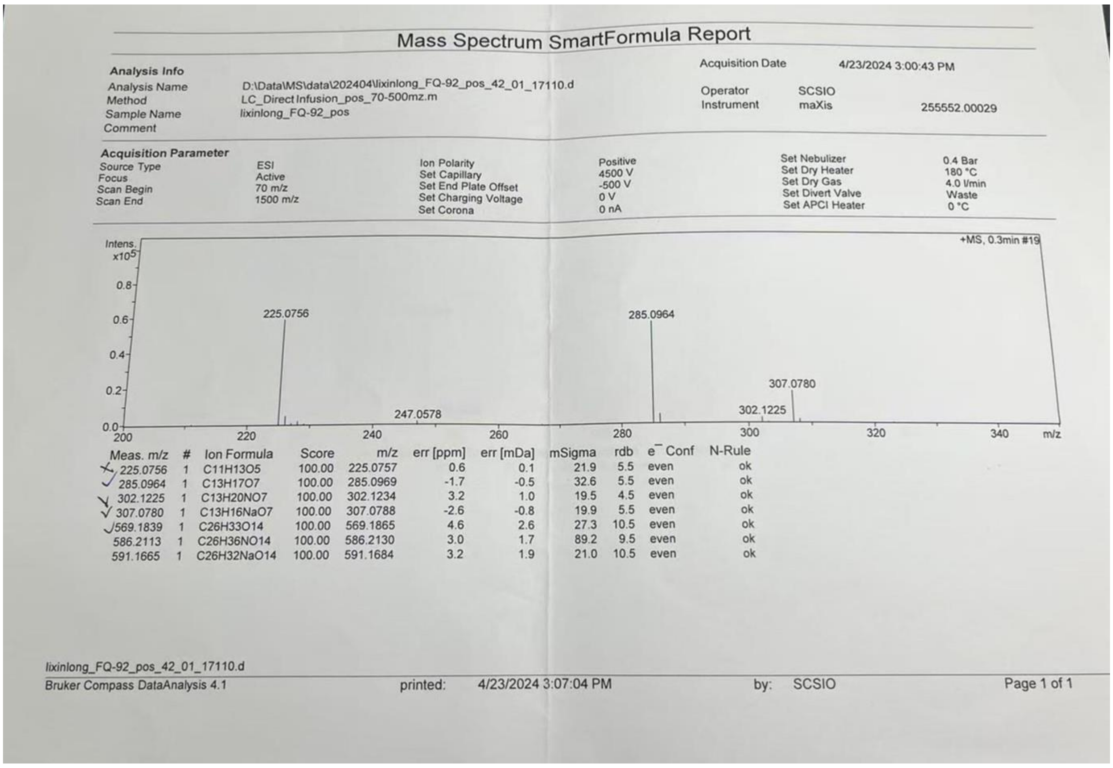

Mass Spectrum SmartFormula Report

Analysis Info

Analysis Name D:\Data\MS\data\202404\lixinlong_FQ-92_pos_42_01_17110.d
Method LC_Direct Infusion_pos_70-500mz.m
Sample Name lixinlong_FQ-92_pos
Comment

Acquisition Date 4/23/2024 3:00:43 PM
Operator SCSIO
Instrument maXis 255552.00029

Acquisition Parameter

Source Type	ESI	Ion Polarity	Positive	Set Nebulizer	0.4 Bar
Focus	Active	Set Capillary	4500 V	Set Dry Heater	180 °C
Scan Begin	70 m/z	Set End Plate Offset	-500 V	Set Dry Gas	4.0 l/min
Scan End	1500 m/z	Set Charging Voltage	0 V	Set Divert Valve	Waste
		Set Corona	0 nA	Set APCI Heater	0 °C

Meas. m/z	#	Ion Formula	Score	m/z	err [ppm]	err [mDa]	mSigma	rdb	e⁻ Conf	N-Rule
225.0756	1	C11H13O5	100.00	225.0757	0.6	0.1	21.9	5.5	even	ok
285.0964	1	C13H17O7	100.00	285.0969	-1.7	-0.5	32.6	5.5	even	ok
302.1225	1	C13H20NO7	100.00	302.1234	3.2	1.0	19.5	4.5	even	ok
307.0780	1	C13H16NaO7	100.00	307.0788	-2.6	-0.8	19.9	5.5	even	ok
569.1839	1	C26H33O14	100.00	569.1865	4.6	2.6	27.3	10.5	even	ok
586.2113	1	C26H36NO14	100.00	586.2130	3.0	1.7	89.2	9.5	even	ok
591.1665	1	C26H32NaO14	100.00	591.1684	3.2	1.9	21.0	10.5	even	ok

lixinlong_FQ-92_pos_42_01_17110.d
Bruker Compass DataAnalysis 4.1 printed: 4/23/2024 3:07:04 PM by: SCSIO Page 1 of 1

Figure S30. HRESIMS spectrum of compound **6.**

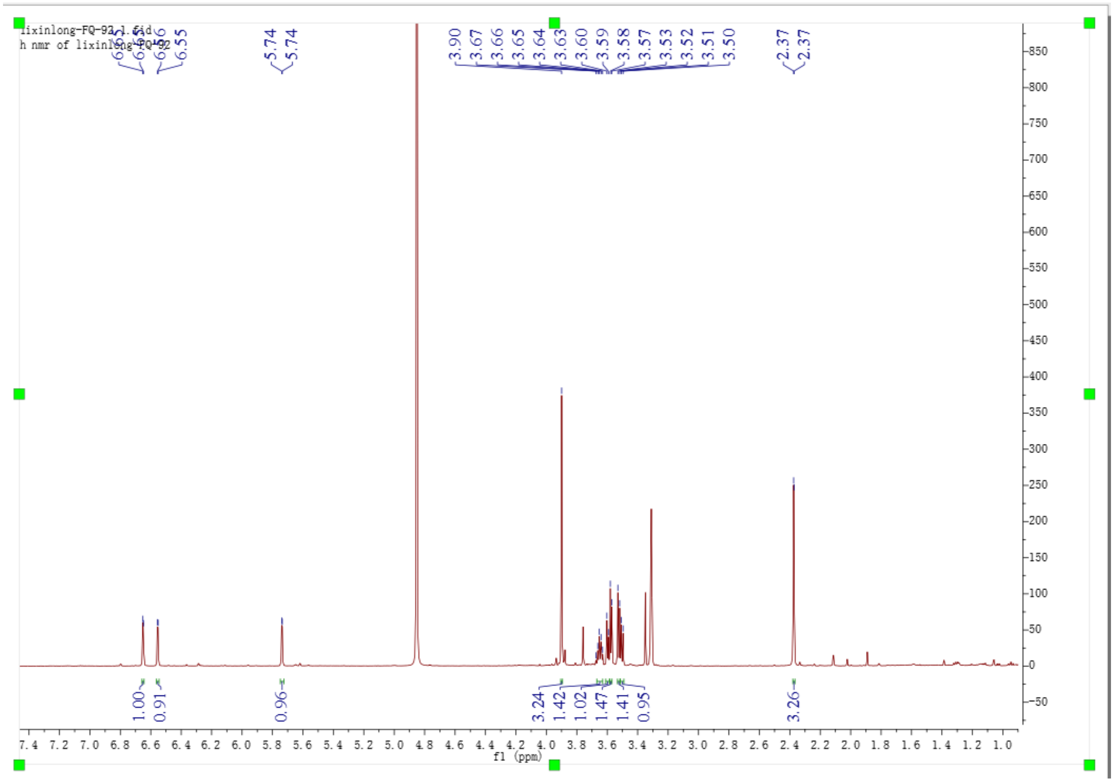

Figure S31. ^{1}H NMR (500 MHz, CD_3OD) spectrum of compound **6**.

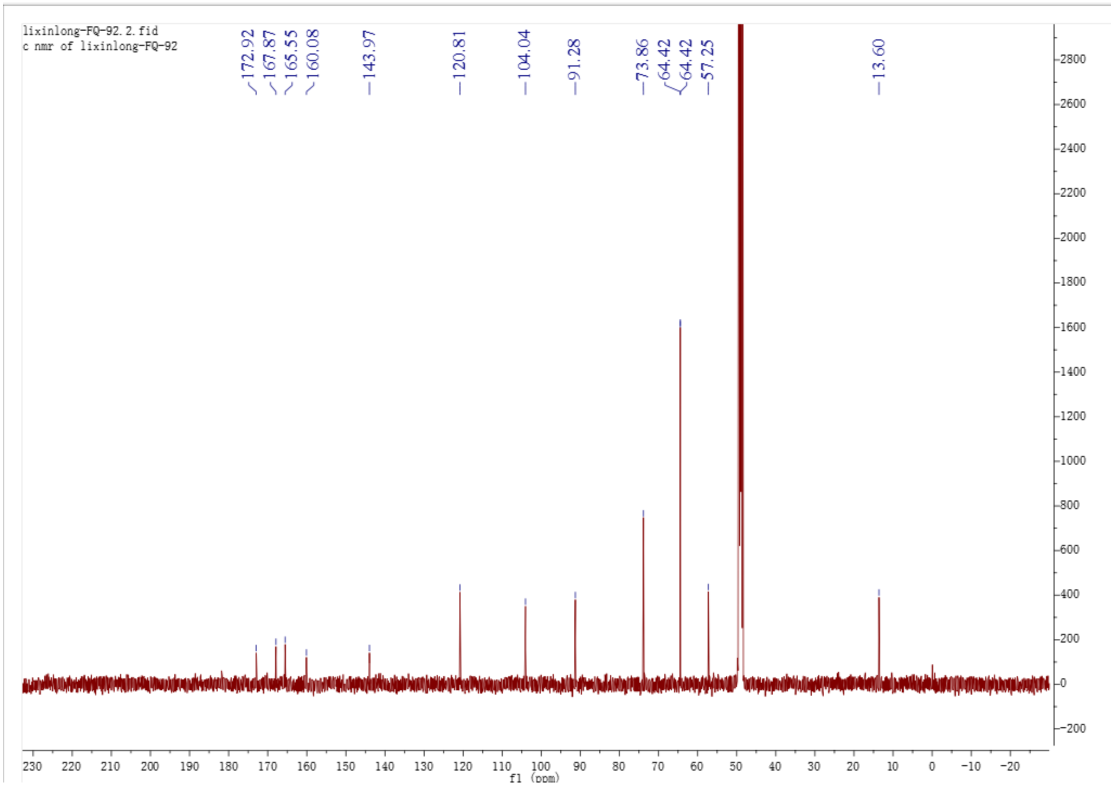

Figure S32. ^{13}C NMR (500 MHz, CD_3OD) spectrum of compound **6**.

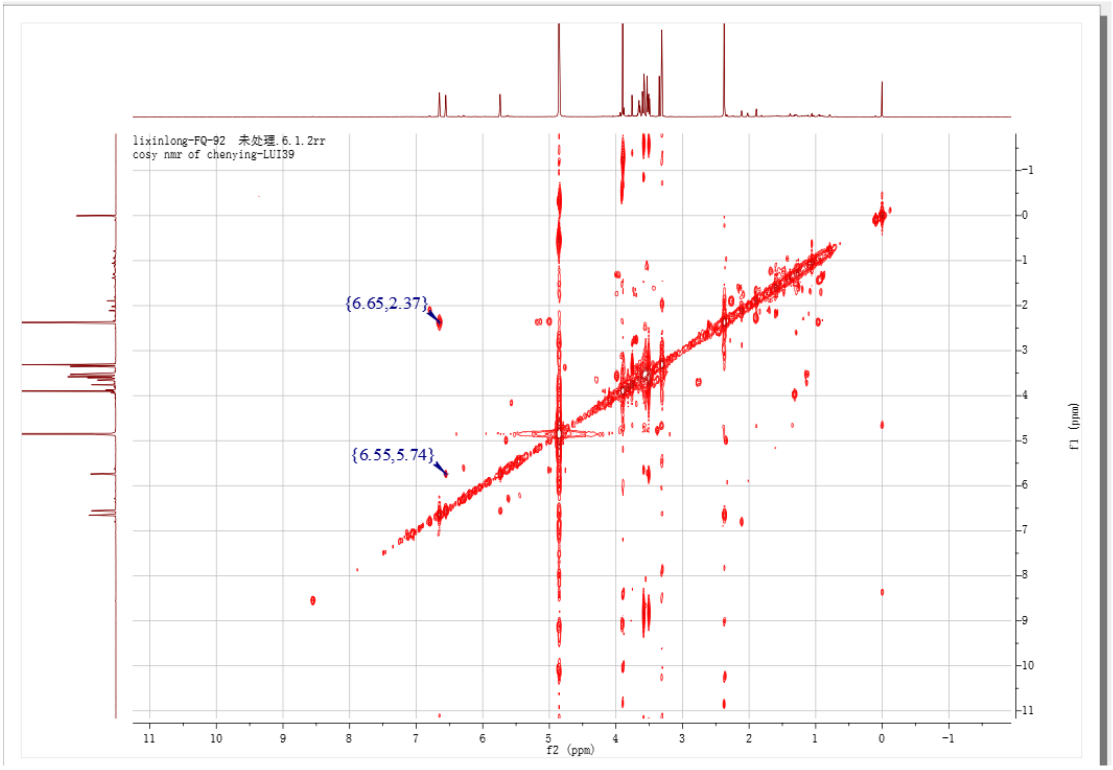

Figure S33. ^{1}H-^{1}H COSY spectrum of compound **6**.

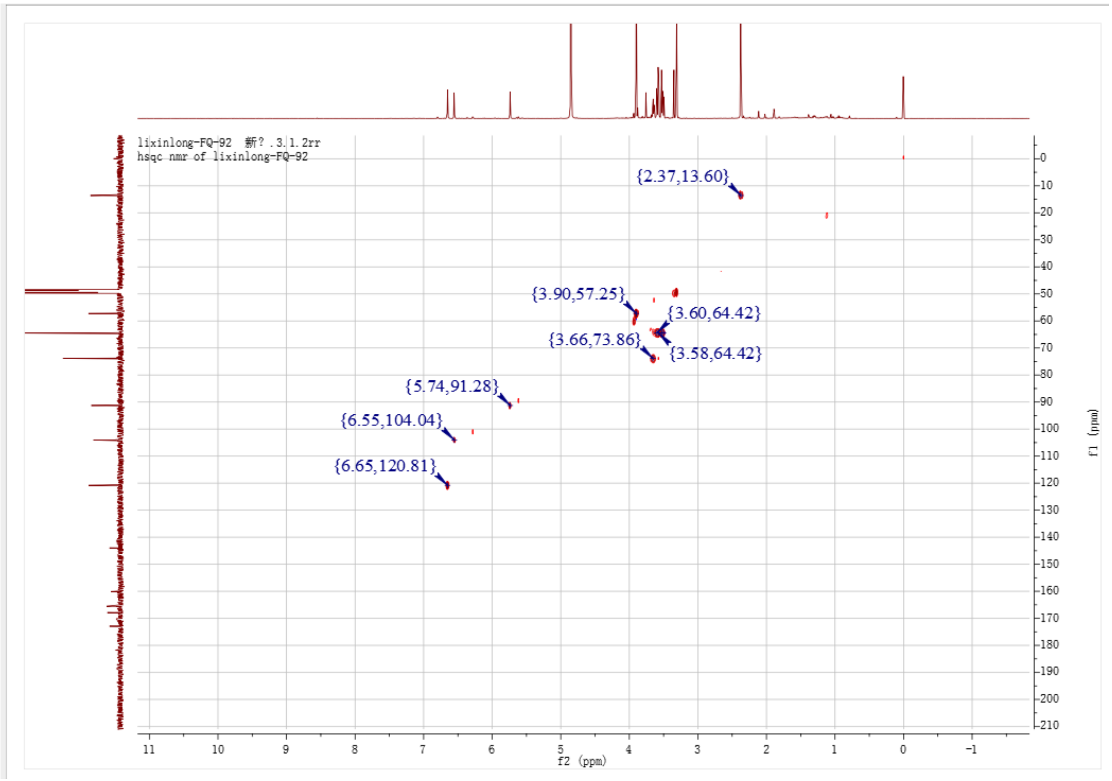

Figure S34. HSQC spectrum of compound **6**.

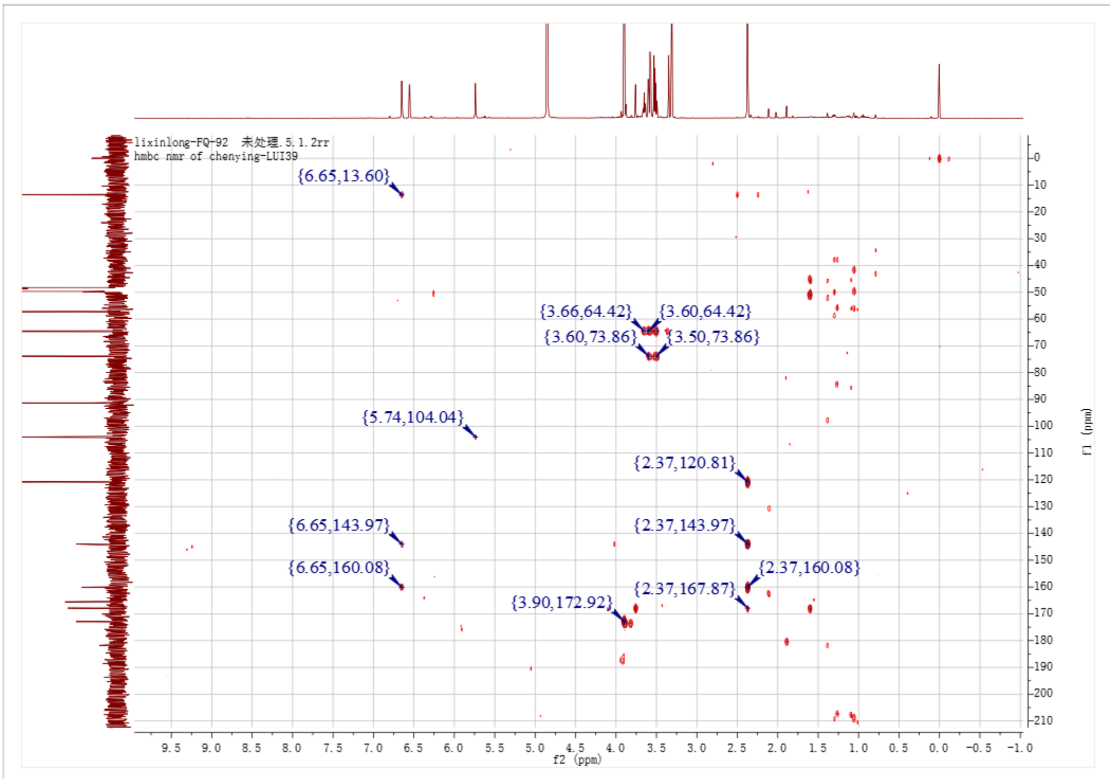

Figure S35. HMBC spectrum of compound **6**.

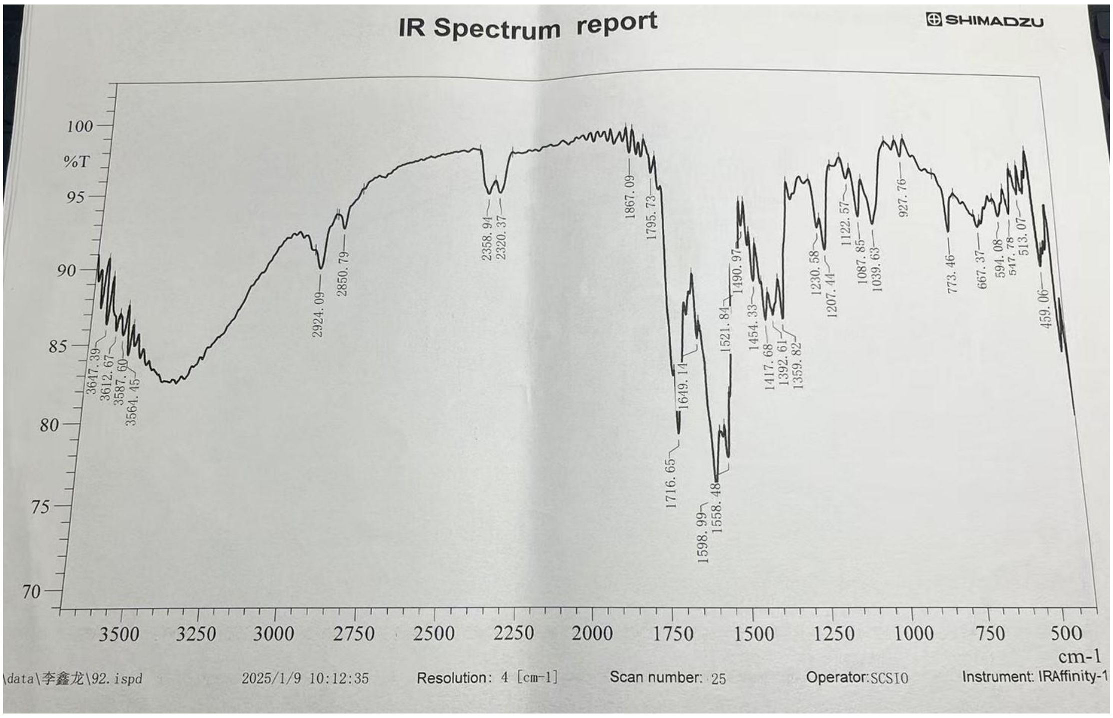

Figure S36. The IR spectrum of compound **6**.

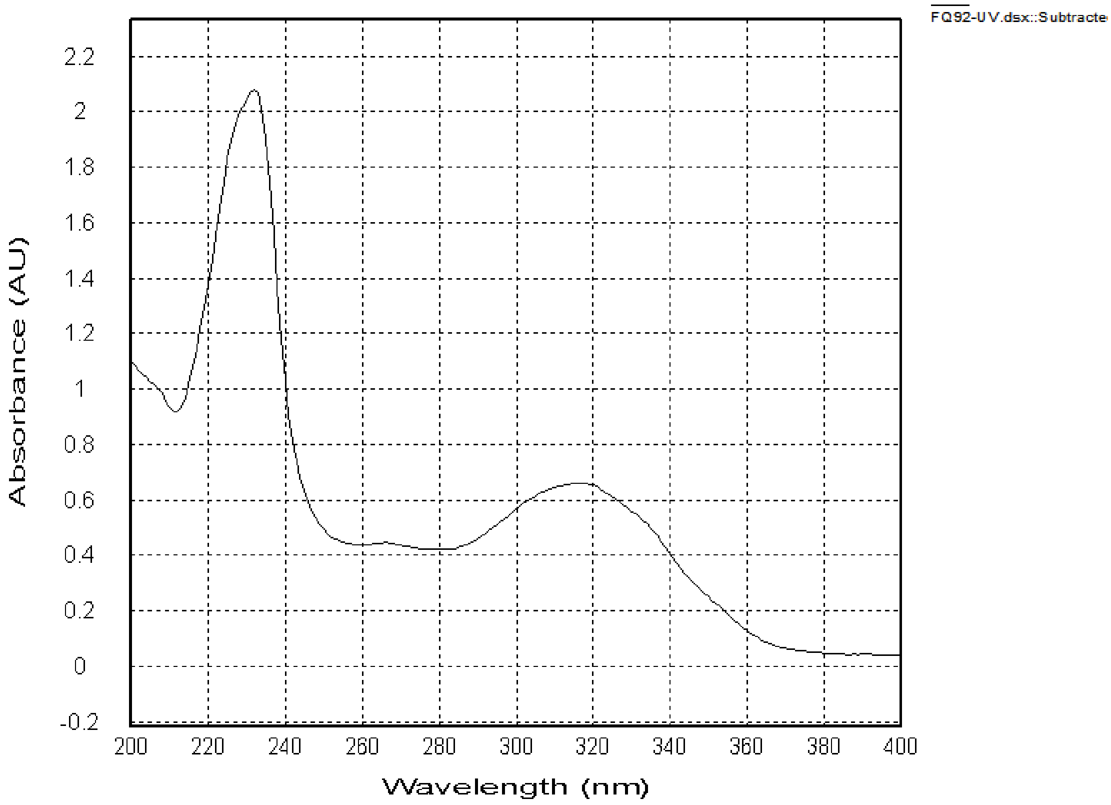

Figure S37. The UV spectrum of compound **6.**

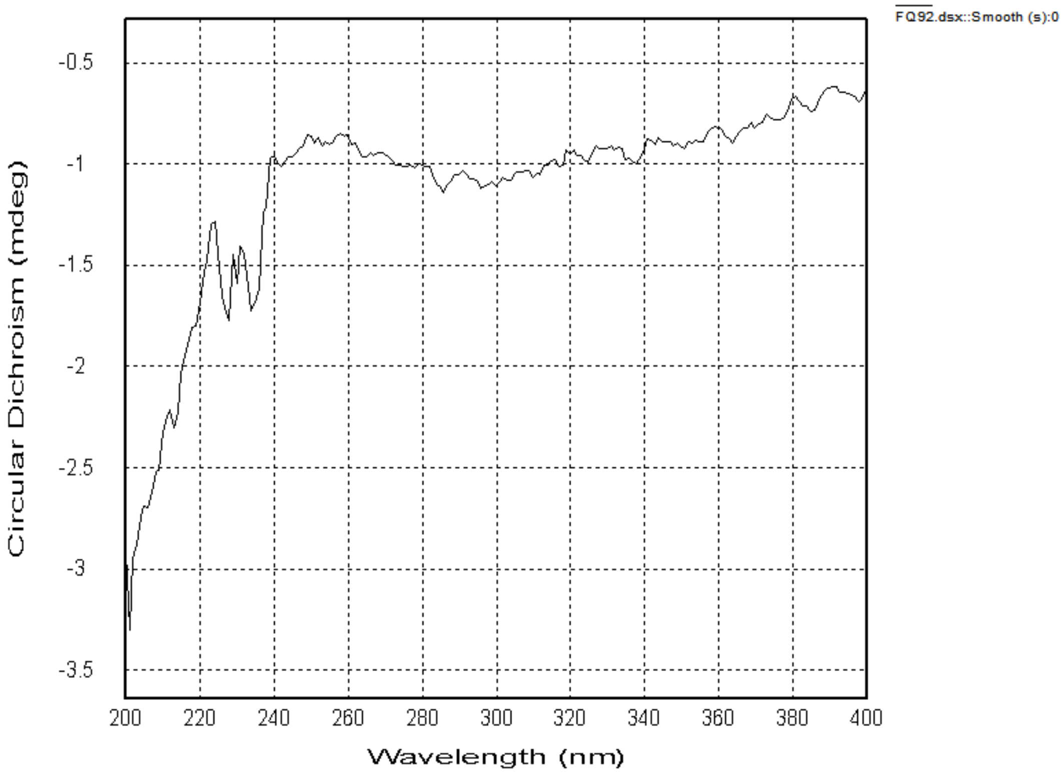

Figure S38. The CD spectrum of compound **6.**

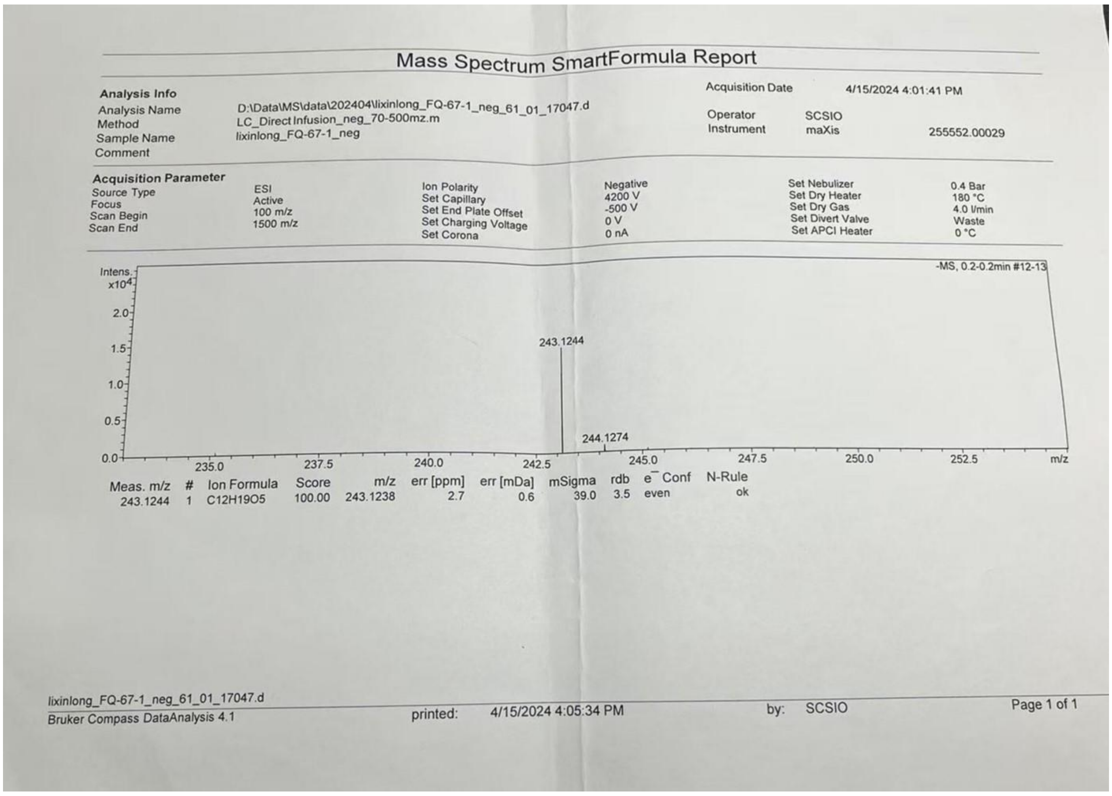

Mass Spectrum SmartFormula Report

Analysis Info
Analysis Name D:\Data\MS\data\202404\lixinlong_FQ-67-1_neg_61_01_17047.d
Method LC_Direct Infusion_neg_70-500mz.m
Sample Name lixinlong_FQ-67-1_neg
Comment

Acquisition Date 4/15/2024 4:01:41 PM
Operator SCSIO
Instrument maXis 255552.00029

Acquisition Parameter					
Source Type	ESI	Ion Polarity	Negative	Set Nebulizer	0.4 Bar
Focus	Active	Set Capillary	4200 V	Set Dry Heater	180 °C
Scan Begin	100 m/z	Set End Plate Offset	-500 V	Set Dry Gas	4.0 l/min
Scan End	1500 m/z	Set Charging Voltage	0 V	Set Divert Valve	Waste
		Set Corona	0 nA	Set APCI Heater	0 °C

Meas. m/z	#	Ion Formula	Score	m/z	err [ppm]	err [mDa]	mSigma	rdb	e⁻ Conf	N-Rule
243.1244	1	C12H19O5	100.00	243.1238	2.7	0.6	39.0	3.5	even	ok

lixinlong_FQ-67-1_neg_61_01_17047.d
Bruker Compass DataAnalysis 4.1 printed: 4/15/2024 4:05:34 PM by: SCSIO Page 1 of 1

Figure S39. HRESIMS spectrum of compound **7.**

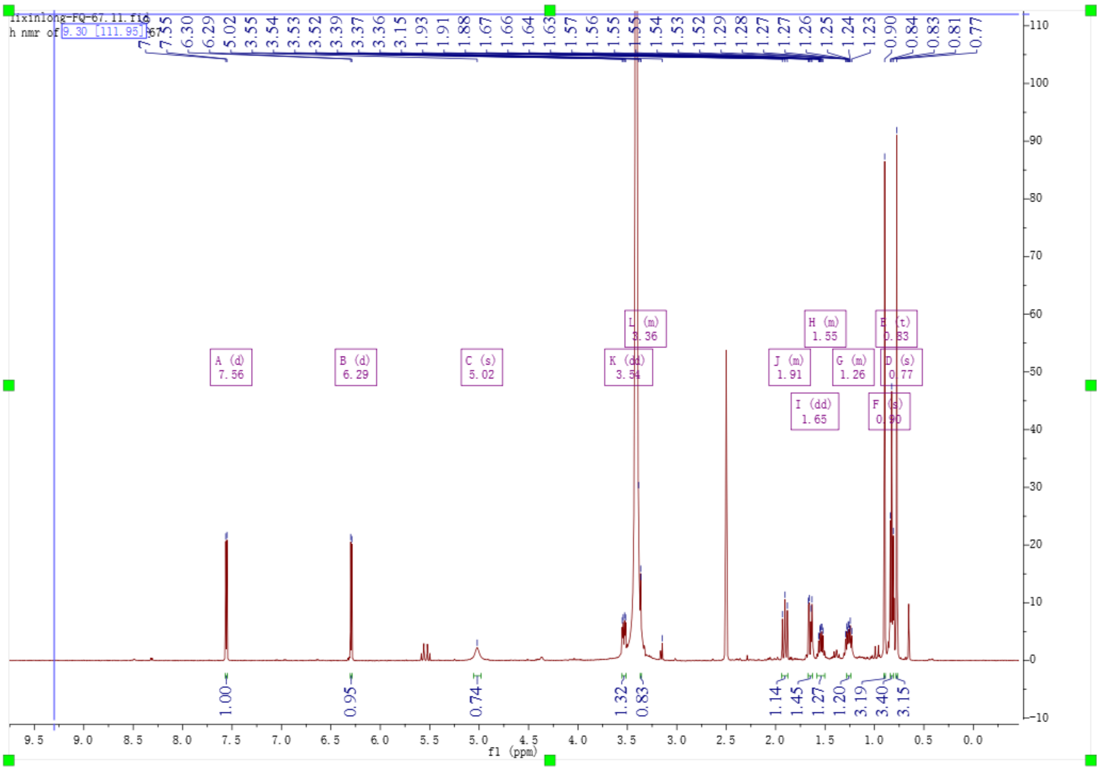

Figure S40. ^{1}H NMR (500 MHz, DMSO-d_6) spectrum of compound **7**.

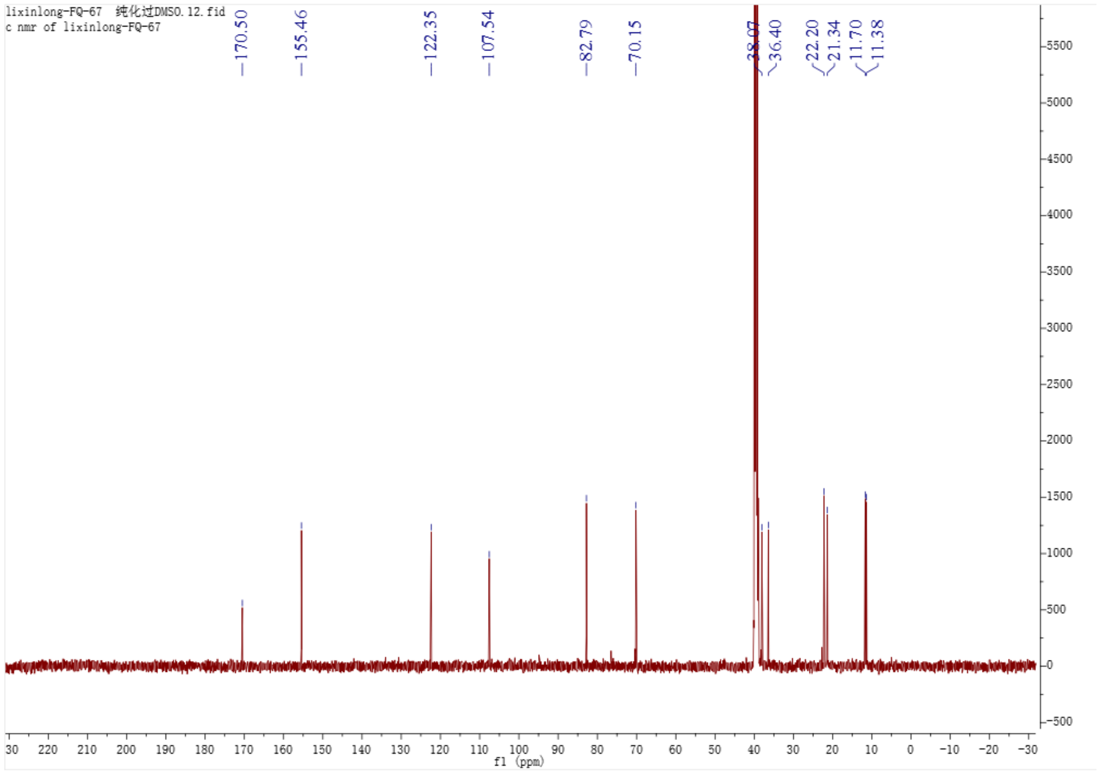

Figure S41. ^{13}C NMR (500 MHz, DMSO-d_6) spectrum of compound **7**.

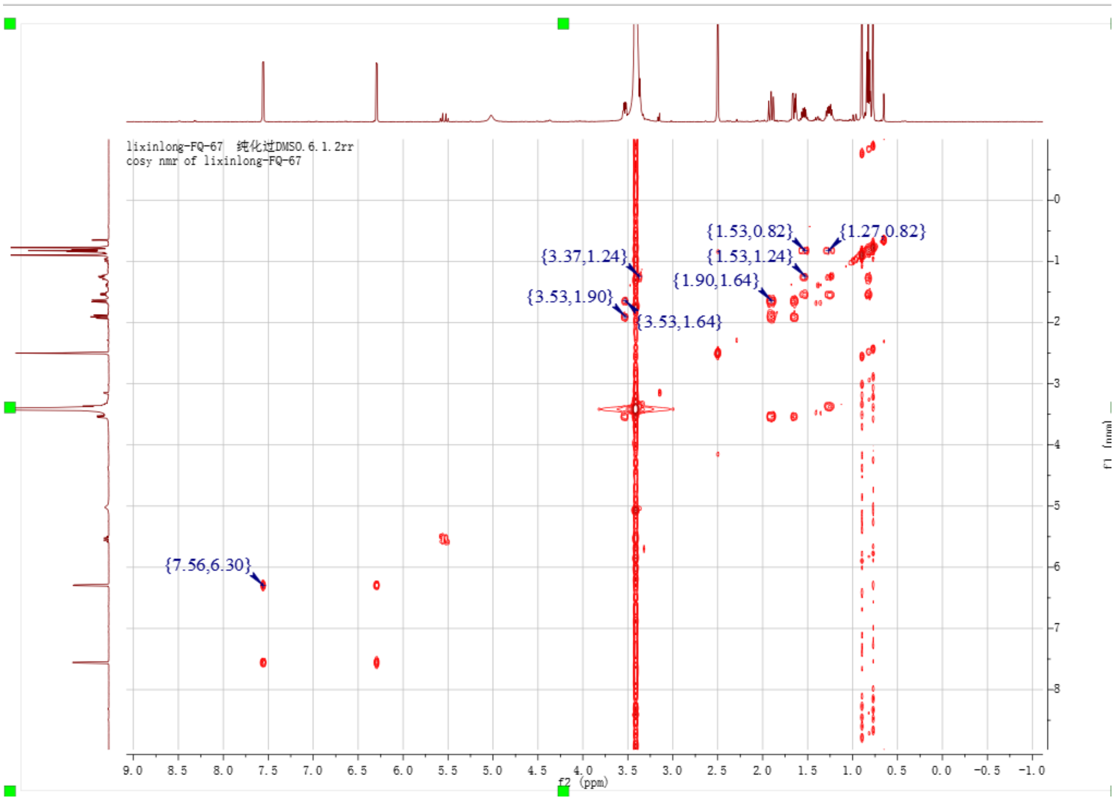

Figure S42. ^{1}H-^{1}H COSY spectrum of compound **7**.

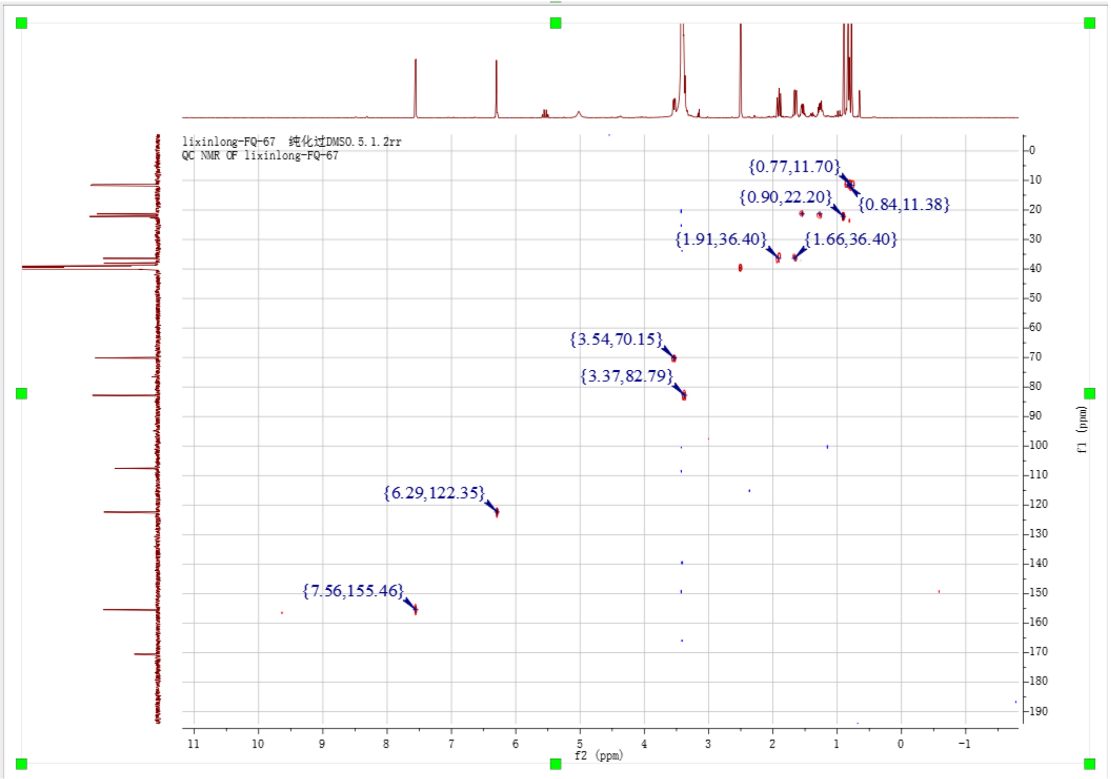

Figure S43. HSQC spectrum of compound **7**.

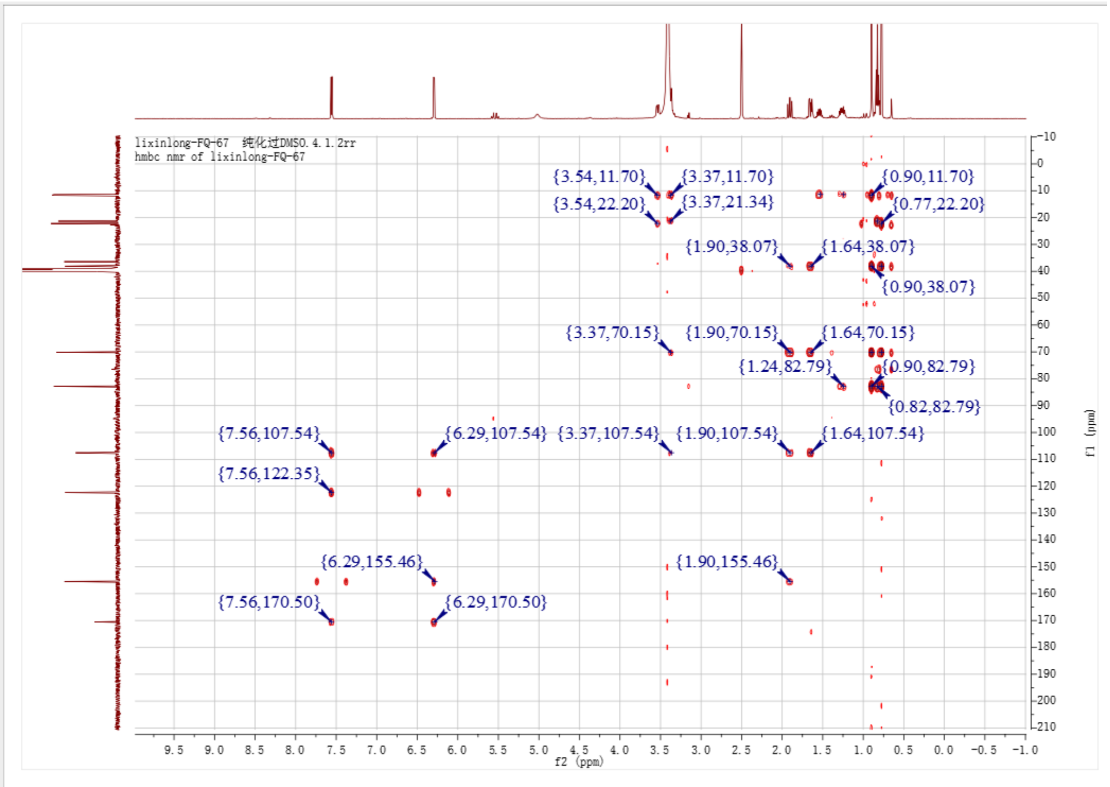

Figure S44. HMBC spectrum of compound **7**.

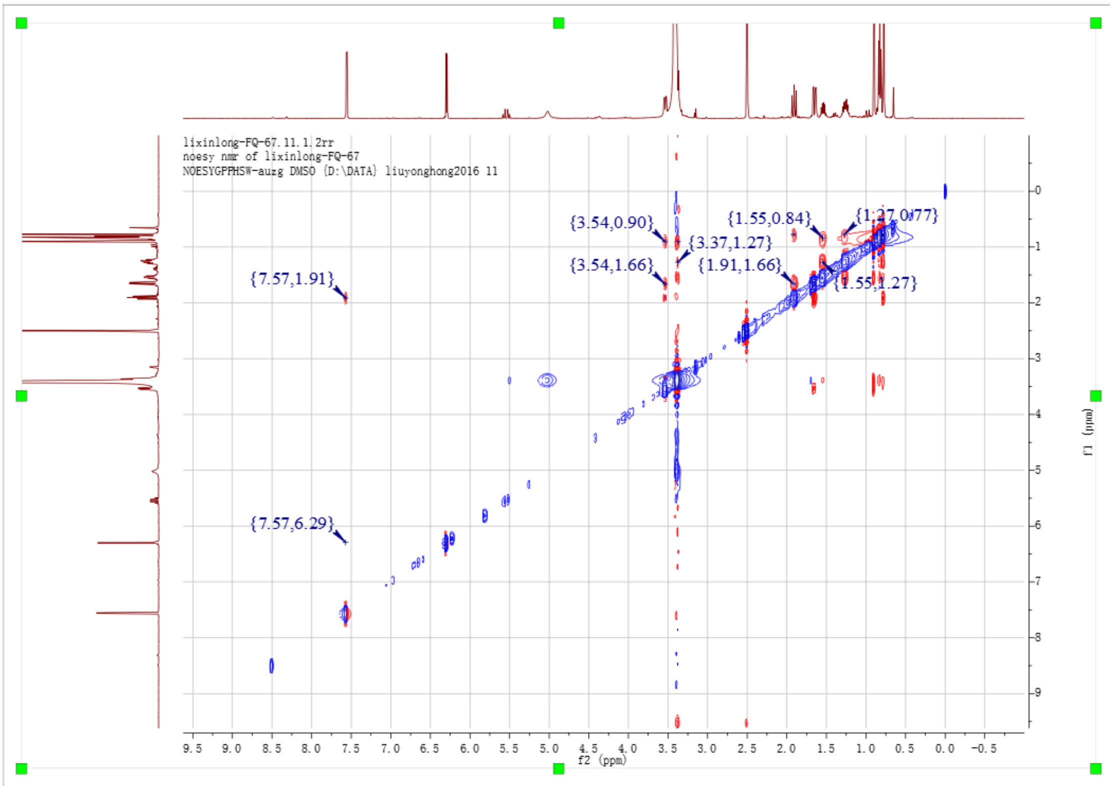

Figure S45. NOESY spectrum of compound **7**.

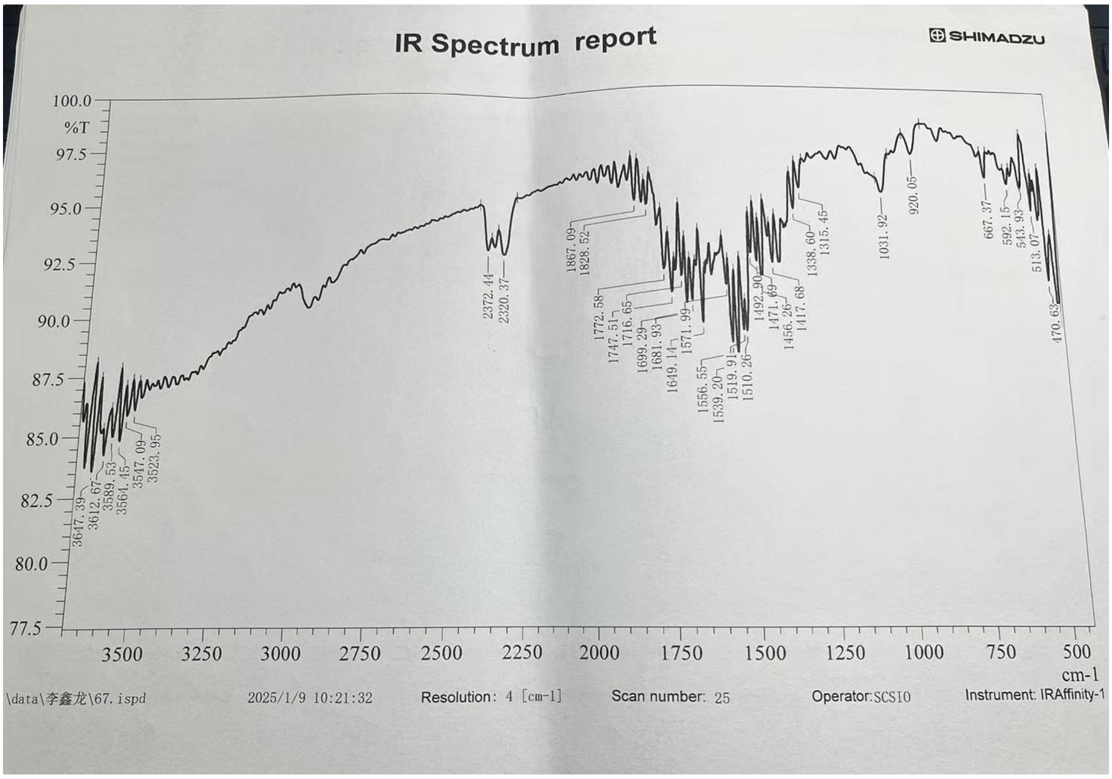

Figure S46. The IR spectrum of compound **7**.

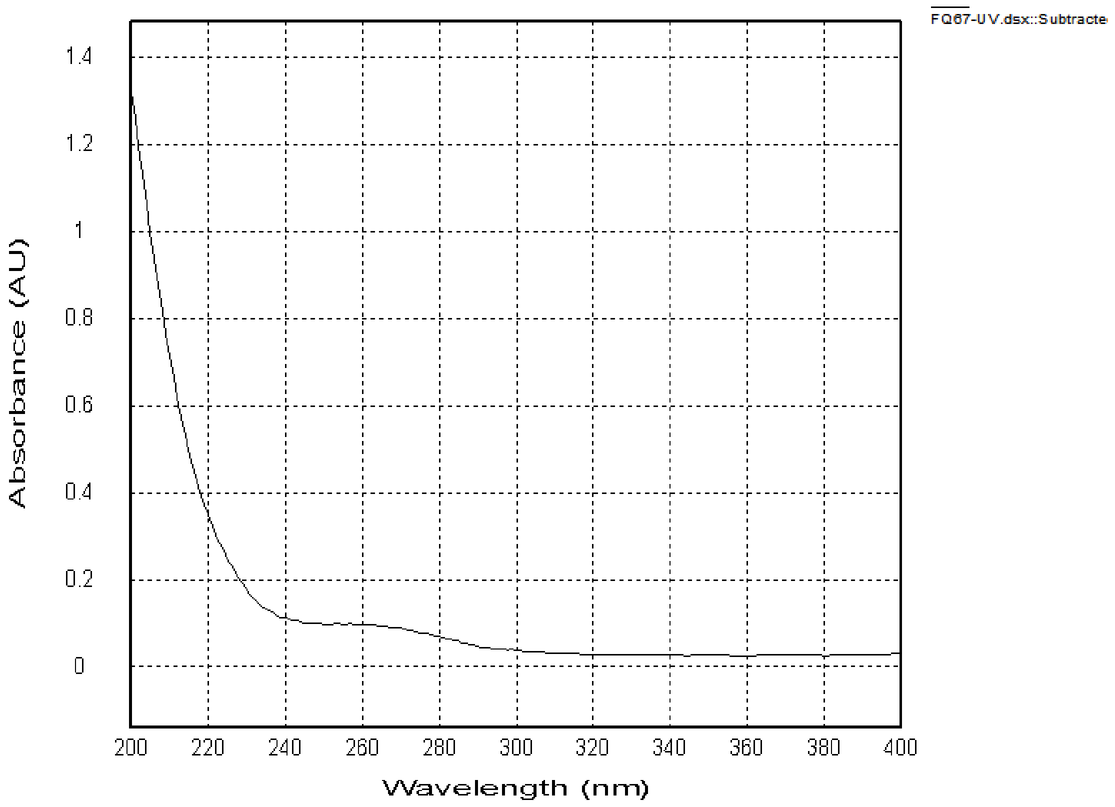

Figure S47. The UV spectrum of compound **7.**

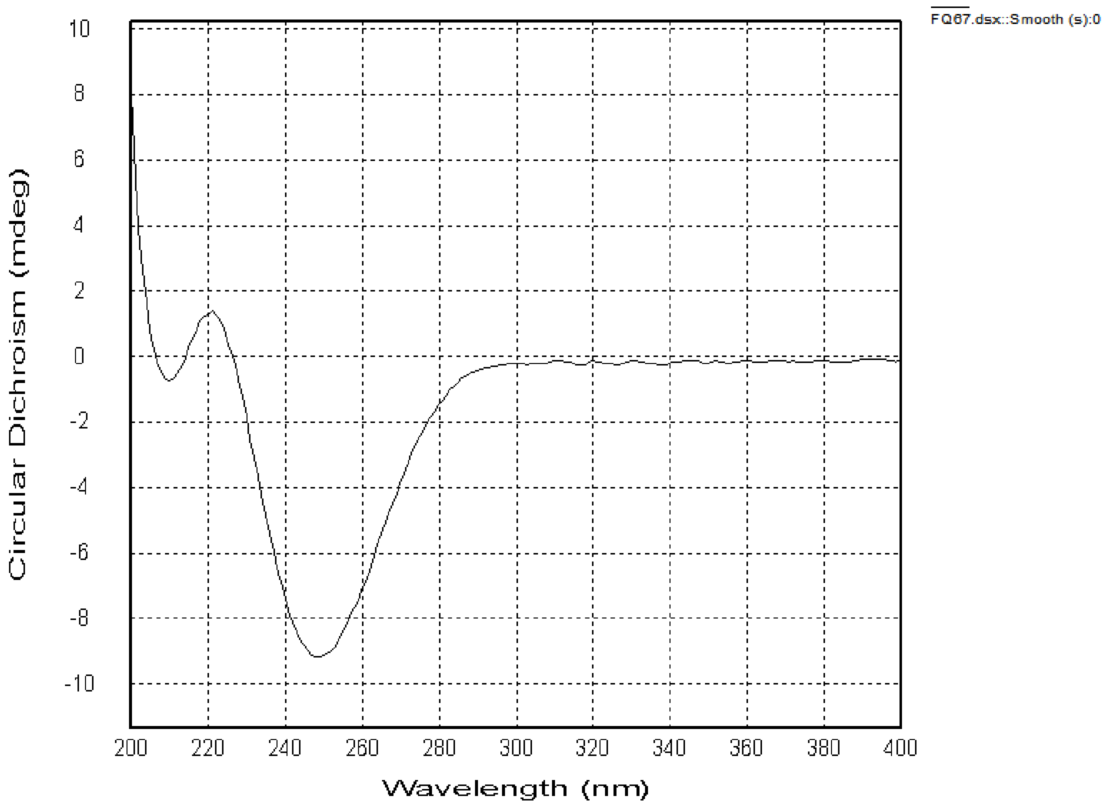

Figure S48. The CD spectrum of compound **7**.

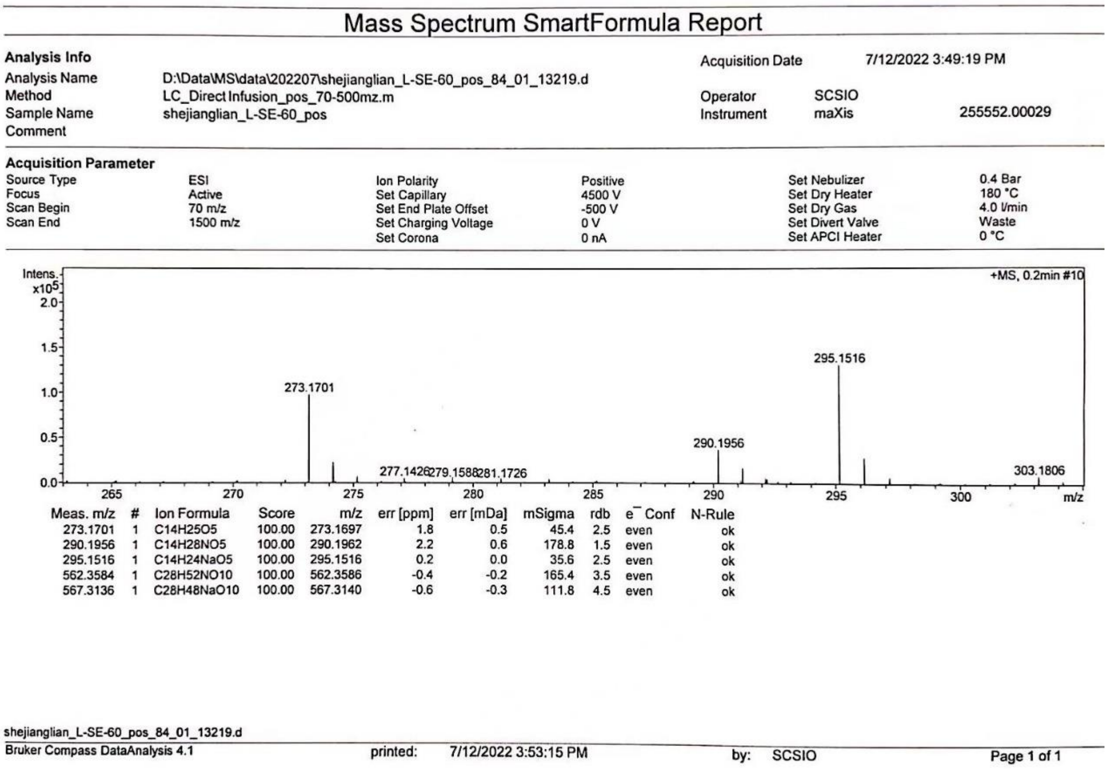

Mass Spectrum SmartFormula Report

**Analysis Info**		Acquisition Date	7/12/2022 3:49:19 PM
Analysis Name	D:\Data\MS\data\202207\shejianglian_L-SE-60_pos_84_01_13219.d		
Method	LC_Direct Infusion_pos_70-500mz.m	Operator	SCSIO
Sample Name	shejianglian_L-SE-60_pos	Instrument	maXis 255552.00029
Comment			

Acquisition Parameter

Source Type	ESI	Ion Polarity	Positive	Set Nebulizer	0.4 Bar
Focus	Active	Set Capillary	4500 V	Set Dry Heater	180 °C
Scan Begin	70 m/z	Set End Plate Offset	-500 V	Set Dry Gas	4.0 l/min
Scan End	1500 m/z	Set Charging Voltage	0 V	Set Divert Valve	Waste
		Set Corona	0 nA	Set APCI Heater	0 °C

Meas. m/z	#	Ion Formula	Score	m/z	err [ppm]	err [mDa]	mSigma	rdb	e⁻ Conf	N-Rule
273.1701	1	C14H25O5	100.00	273.1697	1.8	0.5	45.4	2.5	even	ok
290.1956	1	C14H28NO5	100.00	290.1962	2.2	0.6	178.8	1.5	even	ok
295.1516	1	C14H24NaO5	100.00	295.1516	0.2	0.0	35.6	2.5	even	ok
562.3584	1	C28H52NO10	100.00	562.3586	-0.4	-0.2	165.4	3.5	even	ok
567.3136	1	C28H48NaO10	100.00	567.3140	-0.6	-0.3	111.8	4.5	even	ok

shejianglian_L-SE-60_pos_84_01_13219.d
Bruker Compass DataAnalysis 4.1 printed: 7/12/2022 3:53:15 PM by: SCSIO Page 1 of 1

Figure S49. HRESIMS spectrum of compound **8.**

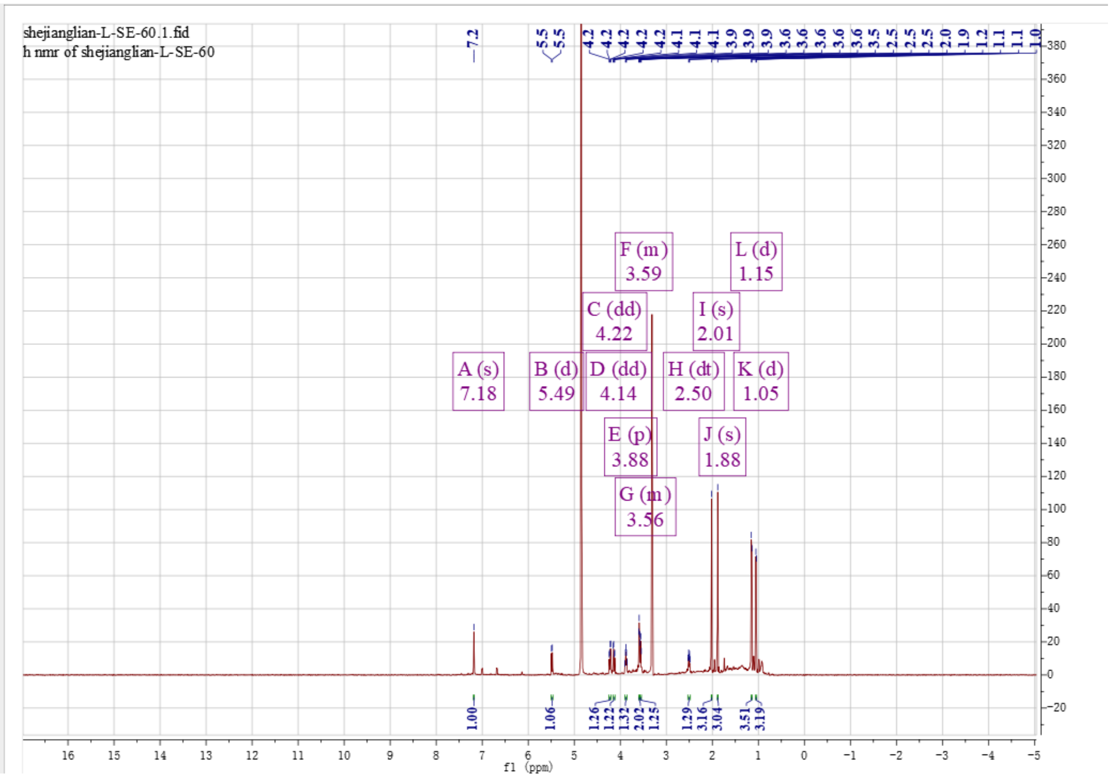

Figure S50. ^{1}H NMR (500 MHz, CD_3OD) spectrum of compound **8**.

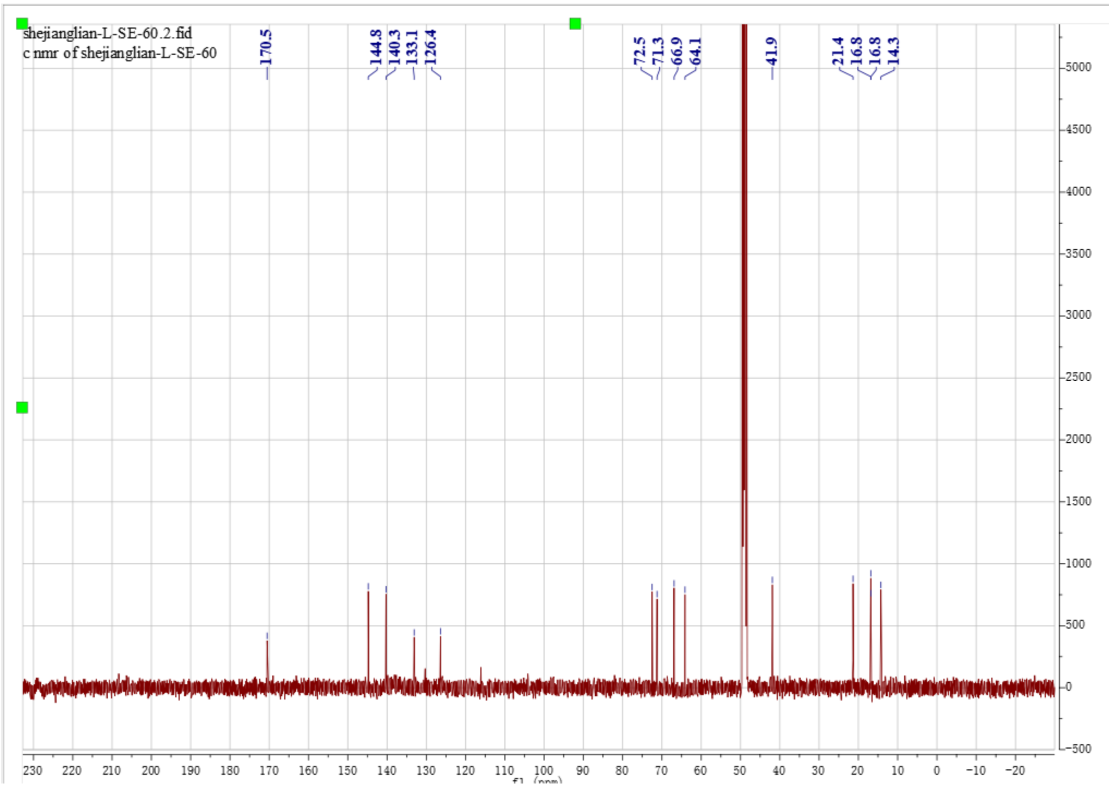

Figure S51. ^{13}C NMR (500 MHz, CD_3OD) spectrum of compound **8**.

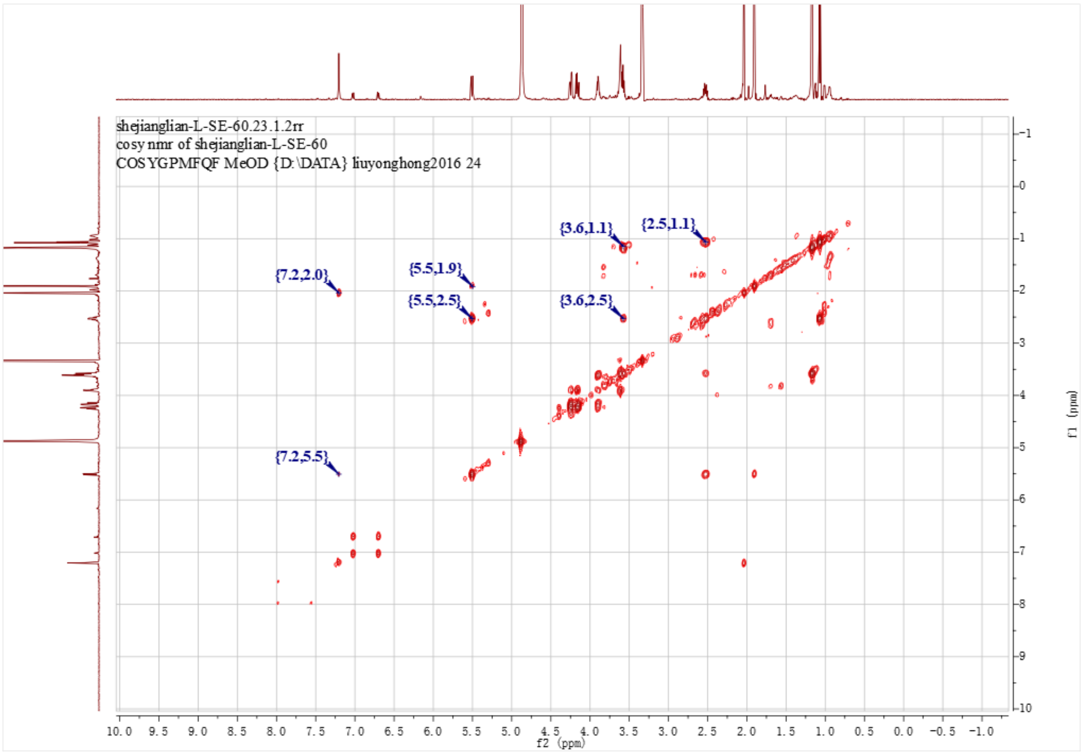

Figure S52. ^{1}H-^{1}H COSY spectrum of compound **8**.

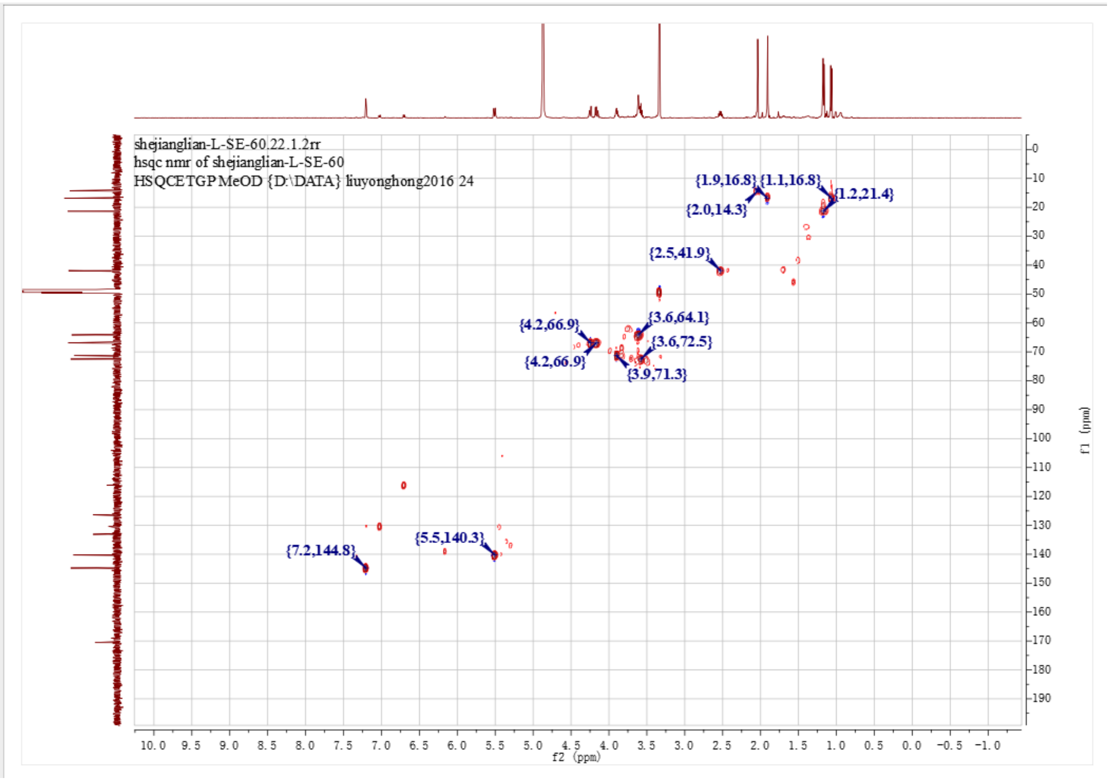

Figure S53. HSQC spectrum of compound **8**.

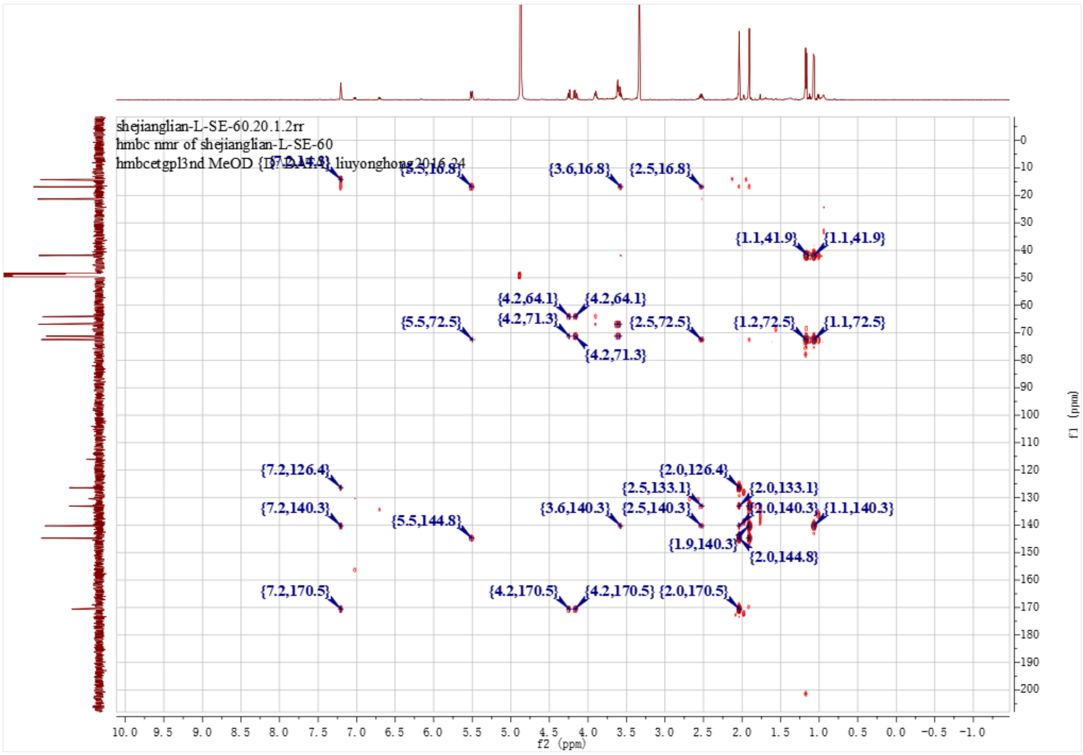

Figure S54. HMBC spectrum of compound **8**.

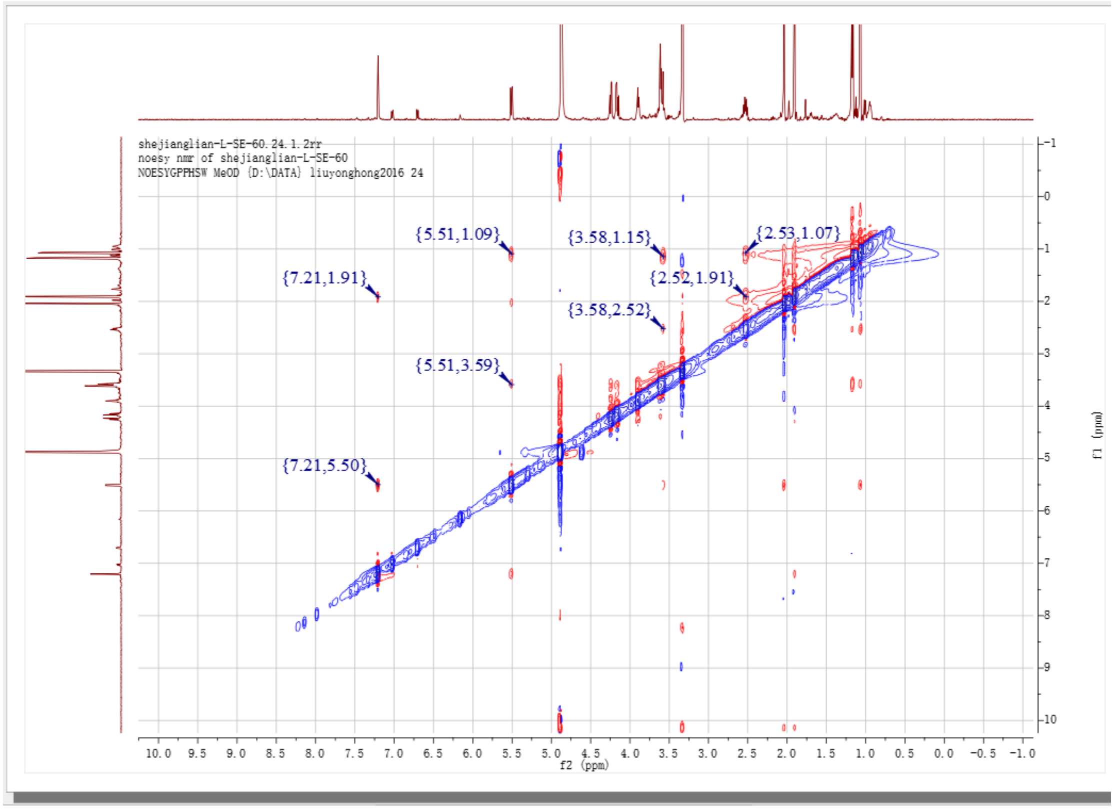

Figure S55. NOESY spectrum of compound **8**.

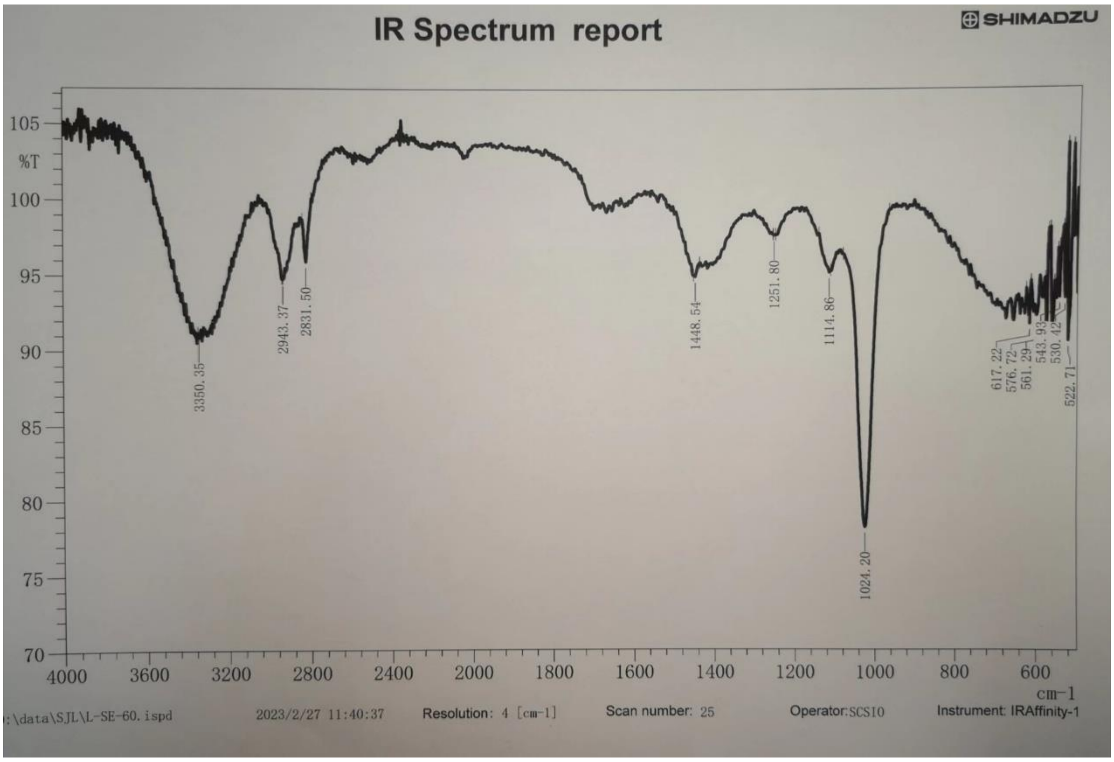

Figure S56. The IR spectrum of compound **8**.

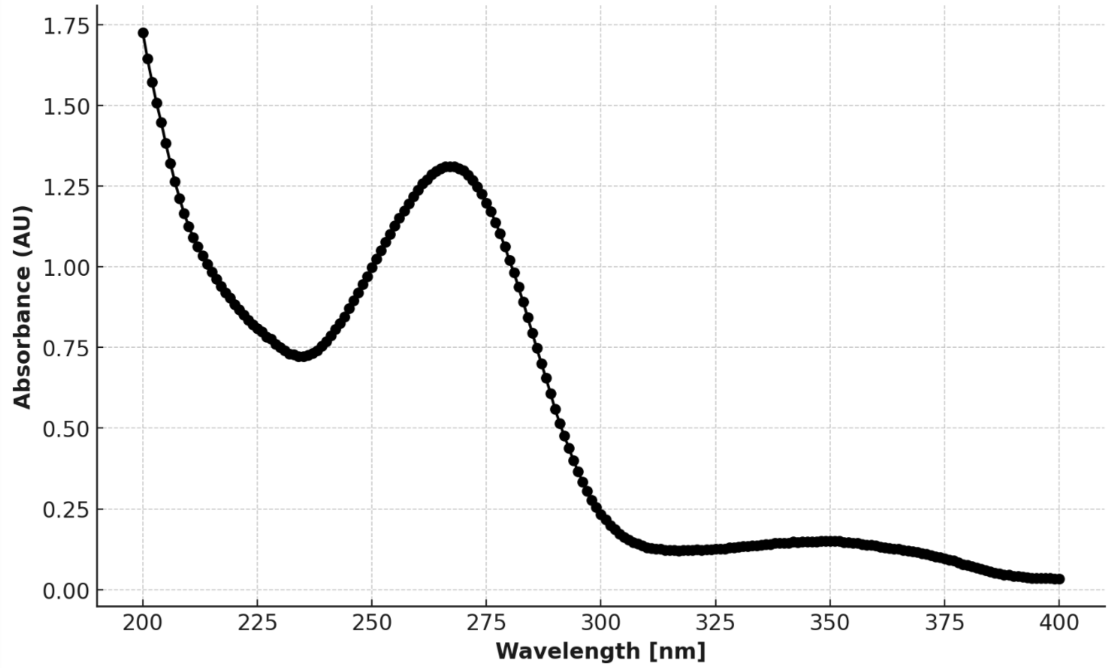

Figure S57. The UV spectrum of compound **8.**

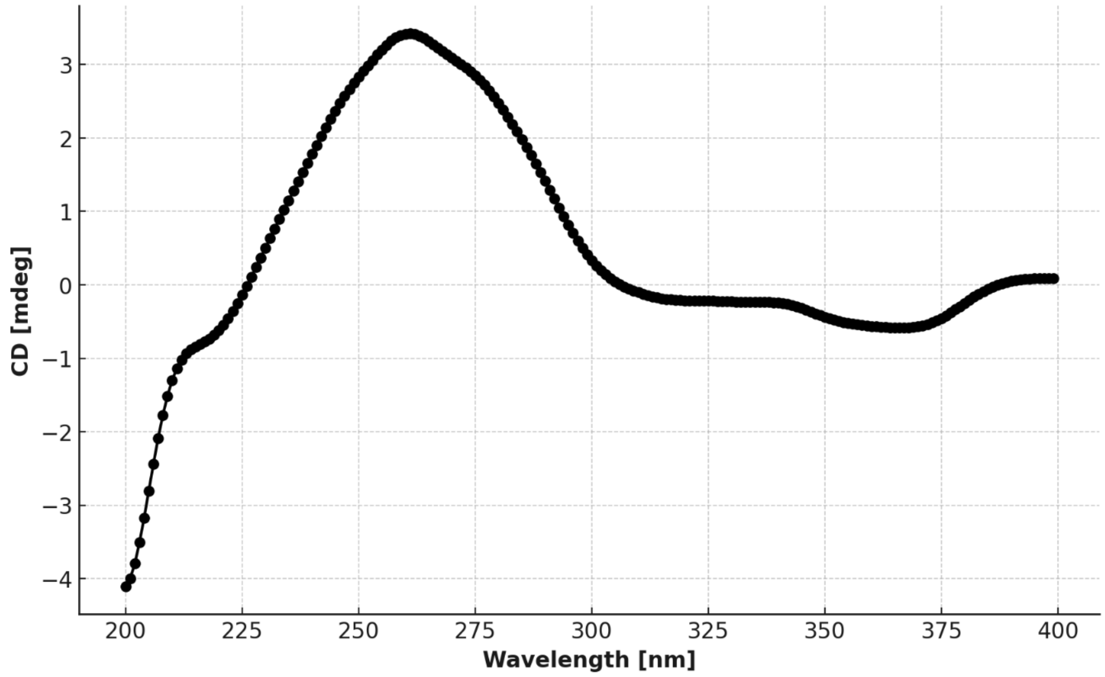

Figure S58. The CD spectrum of compound **8**.

The physicochemical data of the known compounds **3-4**, and **11-16**

Gamahorin (**3**): ^{1}H NMR (500 MHz, Methanol-*d*$_4$) *δ* 7.62 (1H, d, *J* = 7.7 Hz, H-6), 6.88 (1H, d, *J* = 7.7 Hz, H-5), 4.65 (2H, s, H-11), 4.53 (1H, p, *J* = 6.5 Hz, H-3), 2.92 (1H, p, *J* =6.8 Hz, H-4), 1.42 (3H, d, *J* =6.5 Hz, H-9), 1.34 (3H, d, *J* =7.0 Hz, H-10); ^{13}C NMR (125 MHz, Methanol-*d*$_4$) *δ* 170.9 (C, C-1), 160.5 (C, C-8), 144.6 (C, C-4a), 136.0 (CH, C-6), 129.3 (C, C-7), 117.8 (CH, C-5), 108.0 (C, C-8a), 82.6 (CH, C-3), 59.4 (CH_2, C-11), 38.4 (CH, C-4), 19.9 (CH_3, C-9), 17.9 (CH_3, C-10).

Pestalotiopisorin B (**4**): ^{1}H NMR (500 MHz, Methanol-*d*$_4$) *δ* 7.44 (1H, d, *J* = 7.7, H-6), 7.05 (1H, d, *J* = 7.7 Hz, H-5), 4.55 (1H, q, *J* = 6.5 Hz, H-3), 2.22 (3H, s, H-13), 1.43 (3H, d, *J* = 6.6 Hz, H-12), 1.35 (3H, s, H-11); ^{13}C NMR (125 MHz, Methanol-*d*$_4$) *δ* 171.1 (C, C-1), 161.0 (C, C-8), 147.7 (C, C-10), 138.6 (CH, C-6), 126.6 (C, C-7), 114.9 (CH, C-5), 106.8 (C, C-9), 82.9 (CH, C-3), 70.5 (C, C-4), 22.5 (CH_3, C-11), 15.4 (CH_3, C-13), 14.5 (CH_3, C-12).

Butyrolactone-V (**9**): ^{1}H NMR (500 MHz, Methanol-*d*$_4$) *δ* 7.57 (2H, d, *J* = 8.7 Hz, H-3, 5), 6.87 (2H, d, *J* = 8.7 Hz, H-2, 6), 6.55 (1H, dd, *J* = 8.4, 2.2 Hz, H-15, 16), 6.47 (2H, dd, *J* = 5.2, 3.0 Hz, H-19), 3.79 (3H, s, H-12), 3.66 (1H, dd, *J* = 7.5, 5.4 Hz, H-21), 3.45 (2H, s, H-13), 2.77 (1H, dd, *J* = 16.6, 5.4 Hz, H-20), 2.53 (1H, dd, *J* = 16.6, 7.6 Hz, H-20), 1.27 (3H, s, H-24), 1.17 (3H, s, H-23); ^{13}C NMR (125 MHz, Methanol-*d*$_4$) *δ* 171.6 (C-11), 170.5 (C-9), 159.4 (C-17), 153.4 (C-1), 140.1 (C-8), 132.9 (C-19), 130.5 (C-15), 130.3 (C-5, 3), 126.2 (C-18), 123.2 (C-4), 120.5 (C-7), 117.3 (C-16), 116.6 (C-2, 6), 86.8 (C-10), 80.0 (C-22), 70.4 (C-21), 53.9 (C-14), 39.5 (C-13), 32.0 (C-20), 25.8 (C-24), 20.9 (C-23).

Butyrolactone I (**10**):^{1}H NMR (500 MHz, MeOD) δ_H 7.59 (d, *J* = 8.8 Hz, 2H, H-3, 5), 6.87 (d, *J* = 8.7 Hz, 2H, H-2, 6), 6.54 (dd, *J* = 8.2, 2.2 Hz, 1H, H-15), 6.50 (d, *J* = 8.1 Hz, 1H, H-16), 6.42 (d, *J* = 2.2 Hz, 1H, H-19), 5.07 (t, *J* = 3.7 Hz, 1H, H-21), 3.78 (s, 3H, H-12), 3.44 (d, *J* = 8.9 Hz, 2H, H-13), 3.08 (dd, *J* = 7.4, 4.4 Hz, 2H, H-20), 1.67 (s, 3H, H-24), 1.58 (s, 3H, H-23); ^{13}C NMR (125 MHz, MeOD) δ_C 171.6 (C-11), 170.4 (C-9), 159.3 (C-1), 155.1 (C-17), 139.7 (C-8), 133.0 (C-22), 132.4 (C-19), 130.4 (C-3, 5), 129.8 (C-15), 129.2 (C-7), 128.4 (C-18), 125.1 (C-14), 123.5 (C-

21), 123.1 (C-4), 116.6 (C-2, 6), 115.0 (C-16), 86.8 (C-10), 53.8 (C-12), 39.6 (C-13), 28.7 (C-20), 25.9 (C-24), 17.8 (C-23).

aspergillol A (**11**): ^{1}H NMR (500 MHz, Methanol-d_4) δ 7.09 (1H, m, H-3), 7.06 (1H, m, H-5), 6.97 (2H, d, J = 8.5 Hz, H-12, 16), 6.78 (1H, m, H-2), 6.77 (1H, m, H-4), 6.67 (2H, d, J = 8.4 Hz, H-13, 15), 4.20 (2H, t, J = 7.0 Hz, H-9), 3.58 (2H, s, H-7), 2.79 (2H, t, J = 6.9 Hz, H-10); ^{13}C NMR (125 MHz, Methanol-d_4) δ 174.2 (C, C-8), 156.9 (C, C-1), 156.7 (C, C-14), 132.1 (CH, C-5), 131.0 (CH, C-12), 131.0 (CH, C-16), 130.1 (C, C-11), 129.3 (CH, C-3), 122.5 (C, C-6), 120.4 (CH, C-4), 116.2 (CH, C-13), 116.2 (CH, C-15), 115.8 (CH, C-2), 66.9 (CH_2, C-9), 36.8 (CH_2, C-7), 35.2 (CH_2, C-10).

4-hydroxyphenethyl 3-hydroxybenzoate (**12**): ^{1}H NMR (500 MHz, Methanol-d_4) δ 7.08 (1H, t, J = 7.8 Hz, H-5'), 7.01 (2H, d, J = 8.4 Hz, H-2, 6), 6.69 (2H, d, J = 6.3 Hz, H-3, 5), 6.68 (1H, s, H-6'), 6.65 (2H, m, H-2', 4'), 3.68 (2H, t, J = 7.3 Hz, H-α), 2.71 (2H, t, J = 7.2 Hz, H-β); ^{13}C NMR (125 MHz, Methanol-d_4) δ 174.2 (C-7), 160.0 (C-3'), 157.5 (C-4), 136.7 (C-1'), 130.8 (C-2), 130.8 (C-6), 130.5 (C-5'), 130.4 (C-1), 120.5 (C-6'), 117.6 (C-4'), 116.4 (C-3), 116.4 (C-5), 115.5 (C-2'), 64.6 (C-α), 39.4 (C, C-β).

Pestalotiopyrones J (**13**): ^{1}H NMR (500 MHz, Methanol-d_4) δ 6.63 (1H, q, J = 7.1 Hz, H-2'), 6.12 (1H, s, H-5), 5.57 (1H, s, H-3), 3.86 (3H, s, 4-OCH_3), 1.88 (3H, s, 1'-CH_3), 1.86 (3H, d, J = 6.9 Hz, H-3'); ^{13}C NMR (125 MHz, Methanol-d_4) δ 174.1 (C, C-4), 166.9 (C, C-2), 162.7 (C, C-6), 131.0 (CH, C-2'), 128.3 (CH, C-1'), 98.8 (CH, C-5), 88.6 (CH, C-3), 56.9 (CH_3, 4-OCH_3), 14.3 (CH_3, C-3'), 12.1 (CH_3, 1'-CH_3).

Pestalotiopyrones C (**14**): ^{1}H NMR (500 MHz, Methanol-d_4) δ 6.41 (1H, s, H-5), 3.94 (3H, s, 4-OCH_3), 2.58 (2H, q, J = 7.5 Hz, H-1'), 1.85 (3H, s, 3-CH_3), 1.25 (3H, t, J = 7.5 Hz, H-2'); ^{13}C NMR (125 MHz, Methanol-d_4) δ 169.2 (C, C-4), 168.3 (C, C-6), 167.5 (C, C-2), 101.0 (C, C-3), 95.5 (CH, C-5), 57.3 (CH_3, 4-OCH_3), 28.1 (CH, C-1'), 11.6 (CH_3, C-2'), 8.3 (CH_3, 3-CH_3).

Vermopyrone (**15**): ^{1}H NMR (500 MHz, DMSO-d_6) δ 7.26 (1H, s, H-5), 4.00 (3H, s, 4-OMe), 3.32 (3H, s, H-8), 1.87 (3H, s, H-9); ^{13}C NMR (125 MHz, DMSO-d_6) δ

190.7 (C, C-7), 164.4 (C, C-2), 162.4 (C, C-6), 152.4 (C, C-4), 107.2 (C, C-3), 100.1 (CH, C-5), 57.2 (CH_3, 4-OMe), 25.7 (CH_3, C-8), 9.2 (CH_3, C-9).

4-Methoxy-3,6-dimethylpyran-2-one (**16**):^{1}H NMR (500 MHz, DMSO-d_6) δ 6.48 (1H, s, H-5), 3.86 (3H, s, 4-OCH_3), 2.21 (3H, s, 6-CH_3), 1.74 (3H, s, 3-CH_3); ^{13}C NMR (125 MHz, DMSO-d_6) δ 166.2 (C, C-2), 164.5 (C, C-4), 160.8 (C, C-6), 98.4 (C, C-3), 95.4 (CH, C-5), 56.7 (C, 4-OCH_3), 19.6 (C, 6-CH_3), 8.3 (C, 3-CH_3).

The ITS gene sequences data of *Neopestalotiopsis sp.* SCSIO 41422

ATTcCTACCTGATCCGAGGTCAaCCACaAAAAATTGGGGGTTTAGCGGCTGGGAGTTATA
GCACCTAACAAAAGCGAGAAAAAAATTACTACGCTCAGAGGATACTACAAATCCGCCG
TTGTATTTCAGGAACTACAACTATAAAGAAGTAGATTCCCAACACTAAGCTAGGCTTAA
GGGTTGAAATGACGCTCGAACAGGCATGCCCACTAGAATACTAATGGGCGCAATGTGC
GTTCAAAGATTCGATGATTCACTGAATTCTGCAATTCACATTACTTATCGCATTTCGCTG
CGTTCTTCATCGATGCCAGAACCAAGAGATCCGTTGTTGAAAGTTTTGACTTATTAAAA
TAAGACGCTCAGATTACATAAAATAACAAGAGTTTAATGGTCCACCGGCAGCAGCTATA
AGAAGACCTATAACTTCTGCCGAGGCAACAAAAGGTAAGTTCACATGGGTTGG
